# Supplementary material for: Global prevalence and correlates of mpox vaccine acceptance and uptake: a systematic review and meta-analysis
Source: Commun Med (Lond). 2024 Jul 9;4:136. doi: 10.1038/s43856-024-00564-1 (PMC11231226; doi:10.1038/s43856-024-00564-1)
Supplement: Supplementary file 2 — Supplementary Information [file 43856_2024_564_MOESM2_ESM.pdf]

Global prevalence and correlates of mpox vaccine acceptance and uptake: a systematic review and meta-analysis

Sahabi Kabir Sulaiman,[1] Fatimah Isma'il Tsiga-Ahmed,[2] Muhammad Sale Musa,[1] Bello Tijjani Makama,[3] Abdulwahab Kabir Sulaiman,[4,5] Abdulaziz Tijjani Bako[6]

1. Department of Medicine, Yobe State University Teaching Hospital, Damaturu, Nigeria.
2. Department of Community Medicine, Bayero University Kano/Aminu Kano Teaching 9 Hospital, Kano, Nigeria.
3. St Helens and Knowsley Teaching Hospital, NHS Trust, Prescot, UK.
4. Department of Medicine, Murtala Muhammad Specialist Hospital, Kano, Nigeria.
5. Kwanar Dawaki COVID-19 Isolation Center, Kano, Nigeria
6. Department of Neurosurgery, Houston Methodist, Houston, Texas, United States of 14 America.

*Corresponding author:

Dr Sahabi Kabir Sulaiman,

Department of Medicine, Yobe State University Teaching Hospital,

P.M.B. 1072 KM, Potiskum Road, Damaturu, Yobe State, Nigeria

Email: sahabikabir25@gmail.com

CONTENTS:

1. Supplementary Table 1: Results of the consensus critical appraisal of the included cohort studies using the Newcastle-Ottawa scale for cohort studies……………………………………………………………………………..2
2. Publication bias assessment (Supplementary Figures 1 – 28)……………………………………………………….…3
3. Supplementary Table 2: Sensitivity Analysis for LFK Index…………………………………………………………….....31
4. Results of a meta-analysis of mpox vaccine acceptance and uptake (Supplementary Figures 29 - 57)…………….………….32
5. Supplementary References (Arranged serially as contained within the main manuscript file……………………………………73

Supplementary Table 1: Results of the consensus critical appraisal of the included cohort studies using the Newcastle-Ottawa scale for cohort studies

Author (first)	Representativeness of the exposed cohort	Selection of the non-exposed cohort	Ascertainment of exposure	Outcome of interest not present at the start	Comparability of cohorts	Assessment of outcome	Follow-up long enough	Adequacy of follow	TCSotal score	Quality grade
Sagy et al.	*	*	*	*	**	*	*	*	9	High
Salih et al	*	*	*	*	*	*	*	*	8	High
Van Ewijk et al	*	*	*	*	*	*	*	*	8	High
Zucker et al	*	*	*	*	**	*	*		8	High

Publication bias assessment (Supplementary Figures 1 – 28 and Table 2)

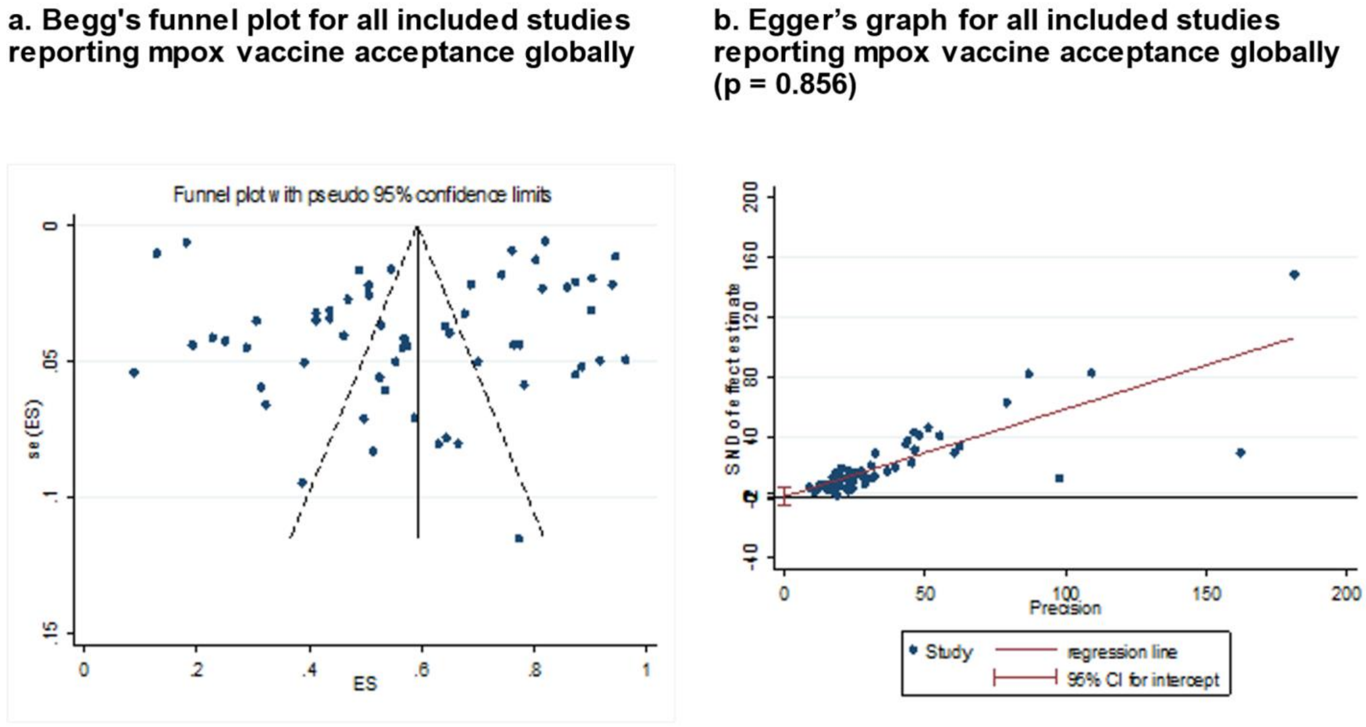

Supplementary Figure 1: Assessment of publication bias among studies reporting acceptance rate across all population groups globally. All the statistical tests performed were two-sided. a, Begg's funnel plot of included studies reporting acceptance rate (n = 59 studies). b, Egger graph of included studies reporting acceptance rate (p = 0.856) (n = 59 studies). ES, Effect Size. SE, Standard Error. SND, Standard Normal Distribution.

a. Begg's funnel plot for all included studies reporting intention to vaccinate against mpox across different population groups globally

b. Egger's graph for all included studies reporting intention vaccinate against mpox across different population group globally (p = 0.993)

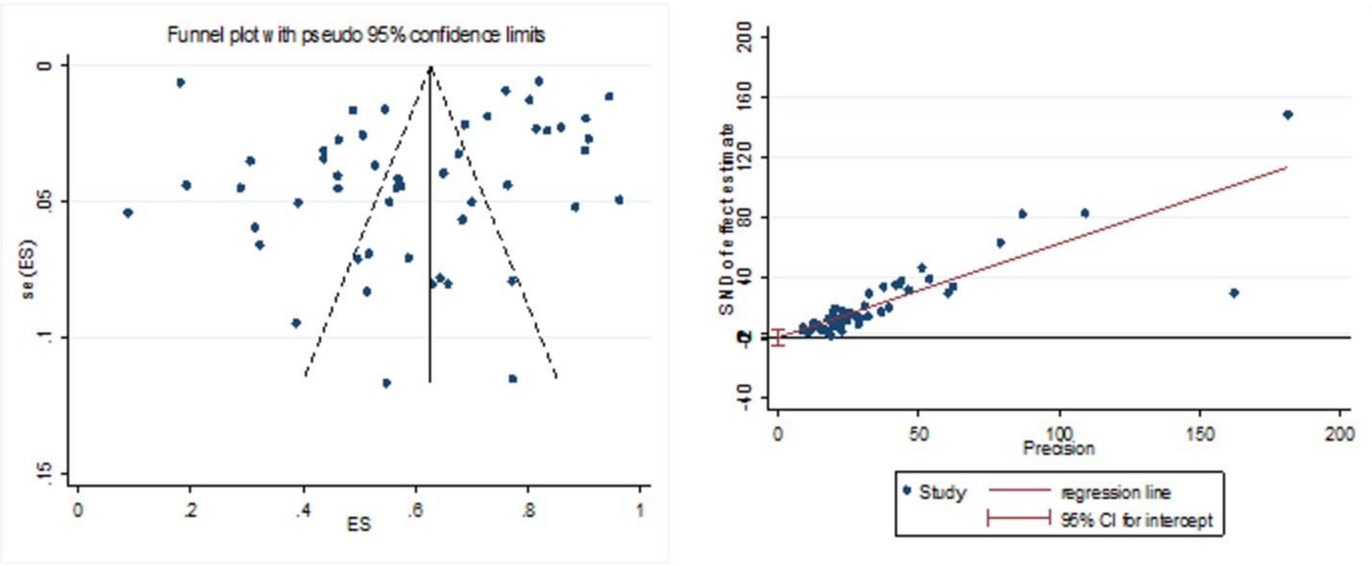

Supplementary Figure 2: Assessment of publication bias among studies reporting the rate of intention to vaccinate against mpox across all population groups globally. All the statistical tests performed were two-sided. a, Begg's funnel plot of included studies reporting the rate of intention to vaccinate globally (n = 51 studies). b, Egger graph of included studies reporting the rate of intention to vaccinate globally (p = 0.993) (n = 51 studies). ES, Effect Size. SE, Standard Error. SND, Standard Normal Distribution.

a. Begg's funnel plot for all included studies reporting mpox vaccine uptake globally

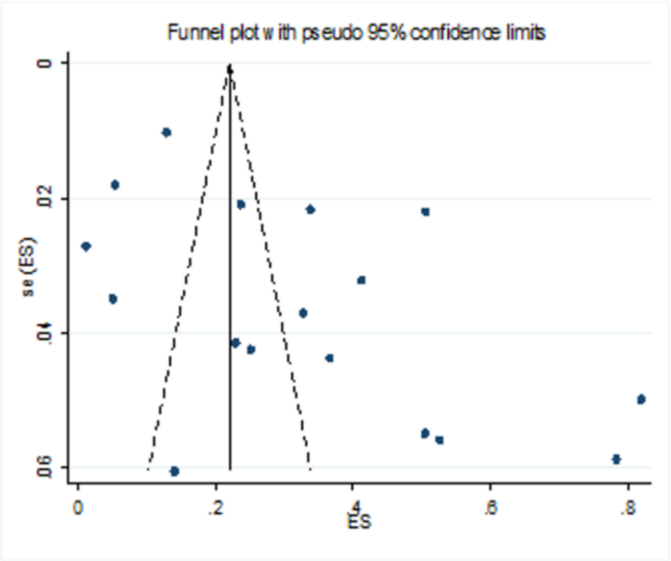

b. Egger's graph for all included studies reporting mpox vaccine uptake globally (p = 0.022)

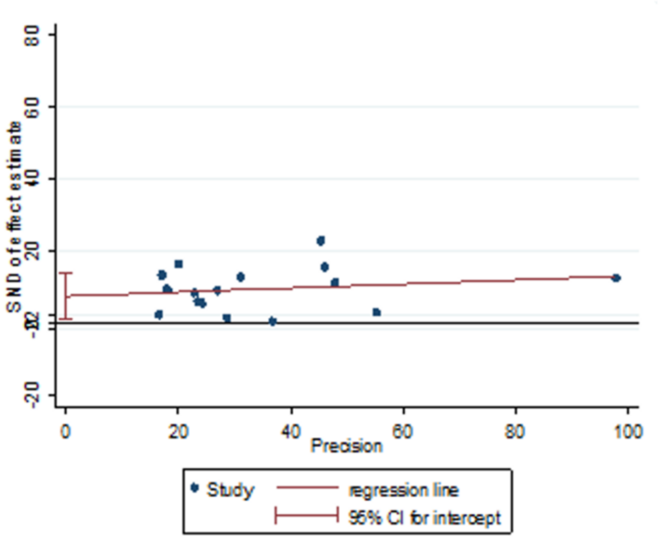

Supplementary Figure 3: Assessment of publication bias among studies reporting the rate of uptake of the mpox vaccine across all population groups globally. All the statistical tests performed were two-sided. a, Begg's funnel plot of included studies reporting the mpox vaccine uptake rate globally (n = 17 studies). b, Egger graph of included studies reporting the mpox vaccine uptake rate globally (p = 0.022) (n = 17 studies). ES, Effect Size. SE, Standard Error. SND, Standard Normal Distribution.

a. Begg's funnel plot for all included studies reporting mpox vaccine uptake among accepting globally

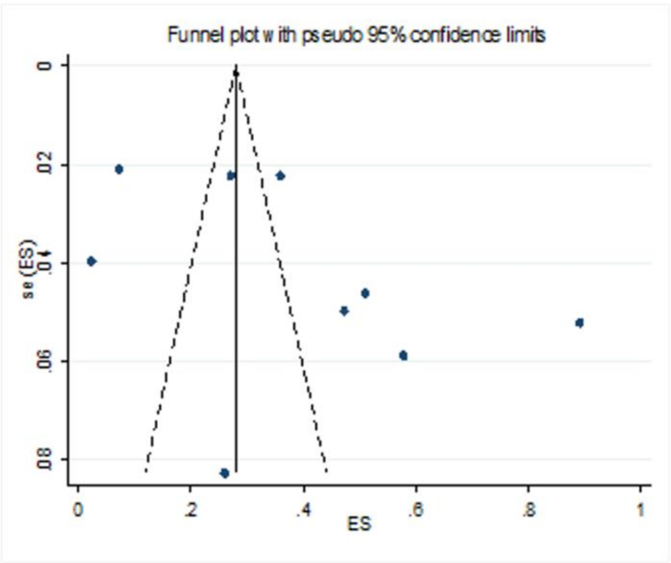

b. Egger's graph for all included studies reporting mpox vaccine uptake among accepting group globally (p = 0.156)

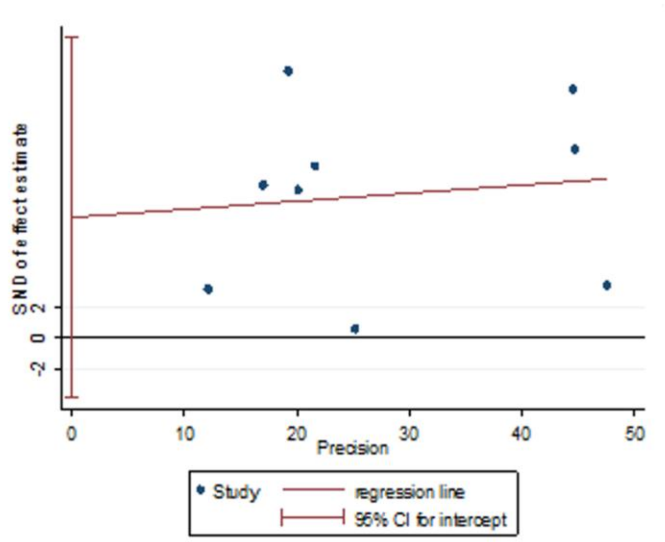

Supplementary Figure 4: Assessment of publication bias among studies reporting the rate of uptake of the mpox vaccine among accepting group across all population groups globally. All the statistical tests performed were two-sided. a, Begg's funnel plot of included studies reporting the mpox vaccine uptake rate among the accepting group globally (p = 0.156) (n = 9 studies). b, Egger graph of included studies reporting the mpox vaccine uptake rate among the accepting group globally (n = 9 studies). ES, Effect Size. SE, Standard Error. SND, Standard Normal Distribution.

a. Begg's funnel plot for all included studies reporting mpox vaccine acceptance among PLHIV globally

b. Egger's graph for all included studies reporting mpox vaccine acceptance among PLHIV globally (p = 0.168)

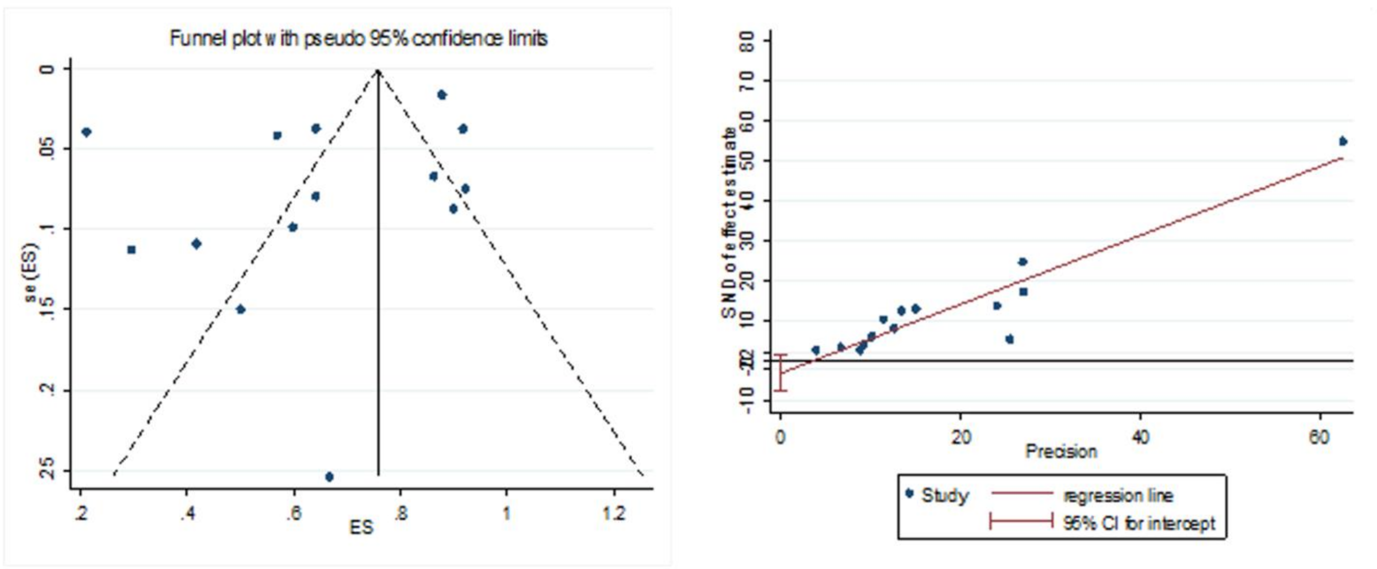

Supplementary Figure 5: Assessment of publication bias among studies reporting acceptance rate PLHIV globally. All the statistical tests performed were two-sided. a, Begg's funnel plot of included studies reporting acceptance rate among PLHIV (n = 14 studies). b, Egger graph of included studies reporting acceptance rate among PLHIV (p = 0.168) (n = 14 studies). ES, Effect Size. SE, Standard Error. SND, Standard Normal Distribution.

a. Begg's funnel plot for all included studies reporting intention to vaccinate against mpox among PLHIV globally

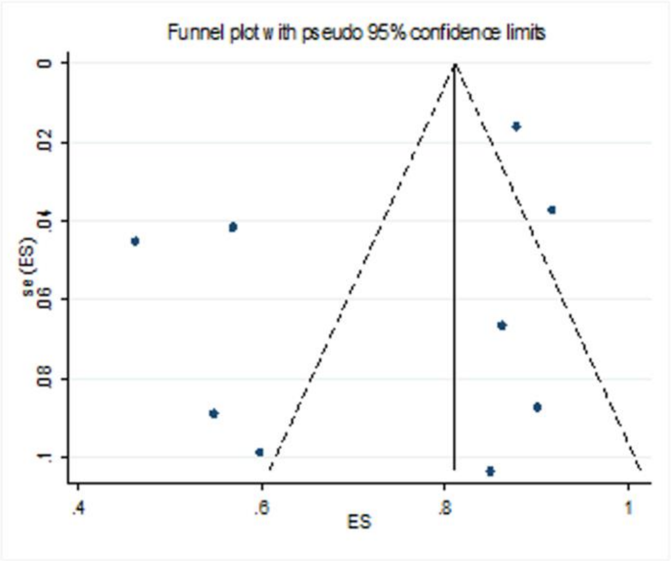

b. Egger's graph for all included studies reporting intention to vaccinate against among PLHIV globally (p = 0.215)

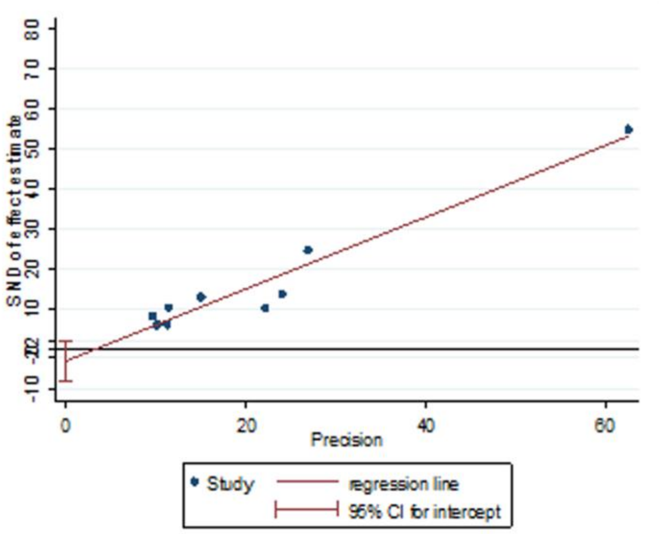

Supplementary Figure 6: Assessment of publication bias among studies reporting rate of intention to vaccinate against the mpox among PLHIV globally. All the statistical tests performed were two-sided. a, Begg's funnel plot of included studies reporting mpox vaccine intention rate among PLHIV (n = 9 studies). b, Egger graph of included studies reporting mpox vaccine intention rate among PLHIV (p = 0.215) (n = 9 studies). ES, Effect Size. SE, Standard Error. SND, Standard Normal Distribution.

a. Begg's funnel plot for all included studies reporting mpox vaccine uptake among PLHIV globally

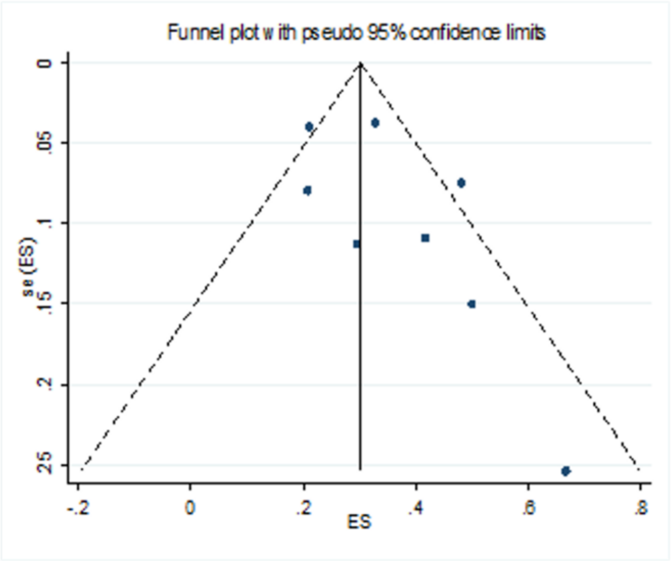

b. Egger's graph for all included studies reporting mpox vaccine uptake among PLHIV globally (p = 0.165)

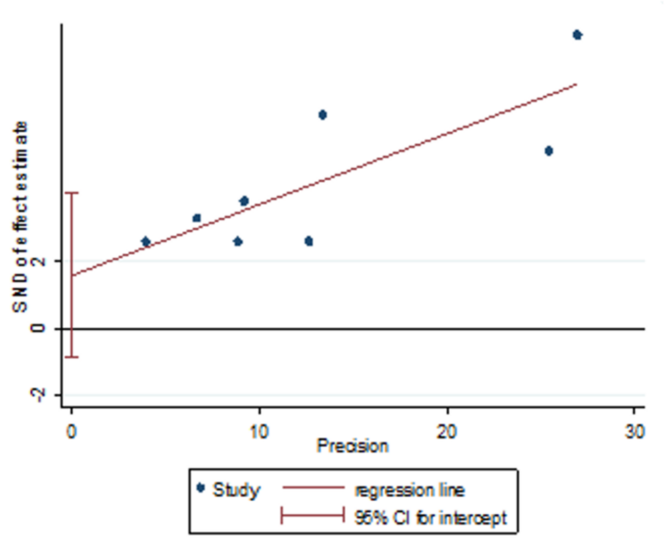

Supplementary Figure 7: Assessment of publication bias among studies reporting rate of uptake of the mpox among PLHIV globally. All the statistical tests performed were two-sided. a, Begg's funnel plot of included studies reporting mpox vaccine uptake rate among PLHIV (n = 9 studies). b, Egger graph of included studies reporting mpox vaccine uptake rate among PLHIV (p = 0.165) (n = 8 studies). ES, Effect Size. SE, Standard Error. SND, Standard Normal Distribution.

a. Begg's funnel plot for all included studies reporting mpox vaccine acceptance among the LGBTQI+ community globally

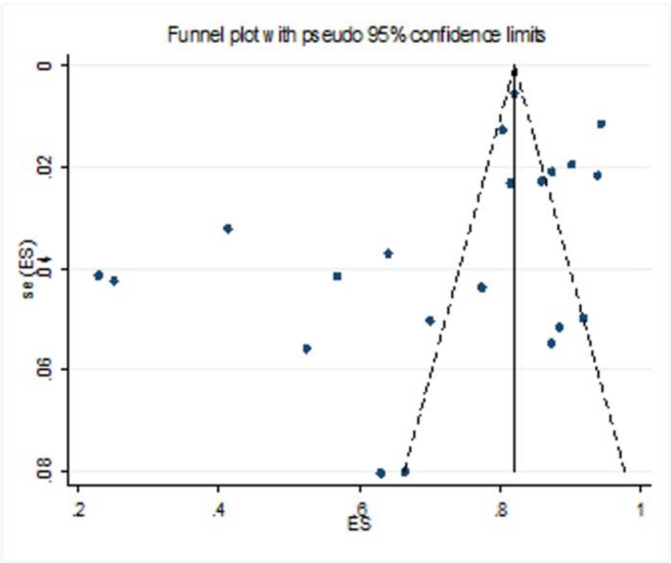

b. Egger's graph for all included studies reporting mpox vaccine acceptance among the LGBTQI+ community globally (p = 0.060)

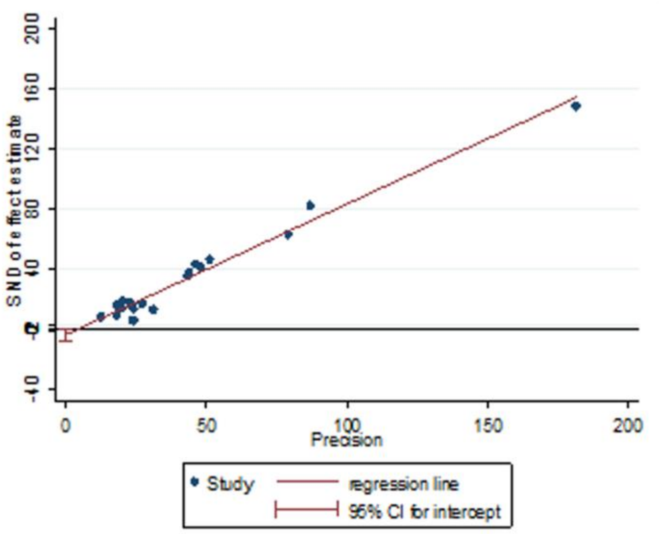

Supplementary Figure 8: Assessment of publication bias among studies reporting acceptance rate among the LGBTQI+ community globally. All the statistical tests performed were two-sided. a, Begg's funnel plot of included studies reporting acceptance rate among the LGBTQI+ community globally (n = 21 studies). b, Egger graph of included studies reporting acceptance rate among the LGBTQI+ community globally (p = 0.060) (n = 21 studies). ES, Effect Size. SE, Standard Error. SND, Standard Normal Distribution.

a. Begg's funnel plot for all included studies reporting intention to vaccinate against mpox among the LGBTQI+ community globally

b. Egger's graph for all included studies reporting intention to vaccinate against mpox among the LGBTQI+ community globally (p = 0.202)

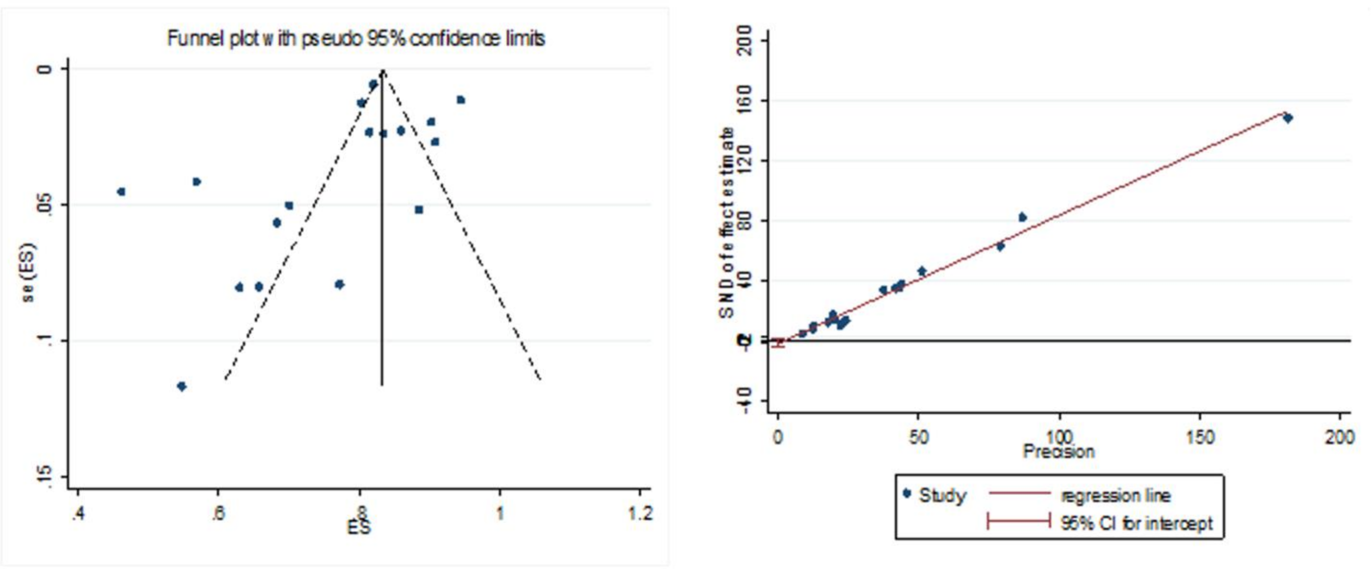

Supplementary Figure 9: Assessment of publication bias among studies reporting the rate of intention to vaccinate against the mpox among the LGBTQI+ community globally. All the statistical tests performed were two-sided. a, Begg's funnel plot of included studies reporting the rate of intention to vaccinate against the mpox among the LGBTQI+ community globally (n = 17 studies). b, Egger graph of included studies reporting the rate of intention to vaccinate against the mpox among the LGBTQI+ community globally (p = 0.202) (n = 17 studies). ES, Effect Size. SE, Standard Error. SND, Standard Normal Distribution.

a. Begg's funnel plot for all included studies reporting mpox vaccine uptake among the LGBTQI+ community globally

b. Egger's graph for all included studies reporting mpox vaccine uptake among the LGBTQI+ community globally (p = 0.081)

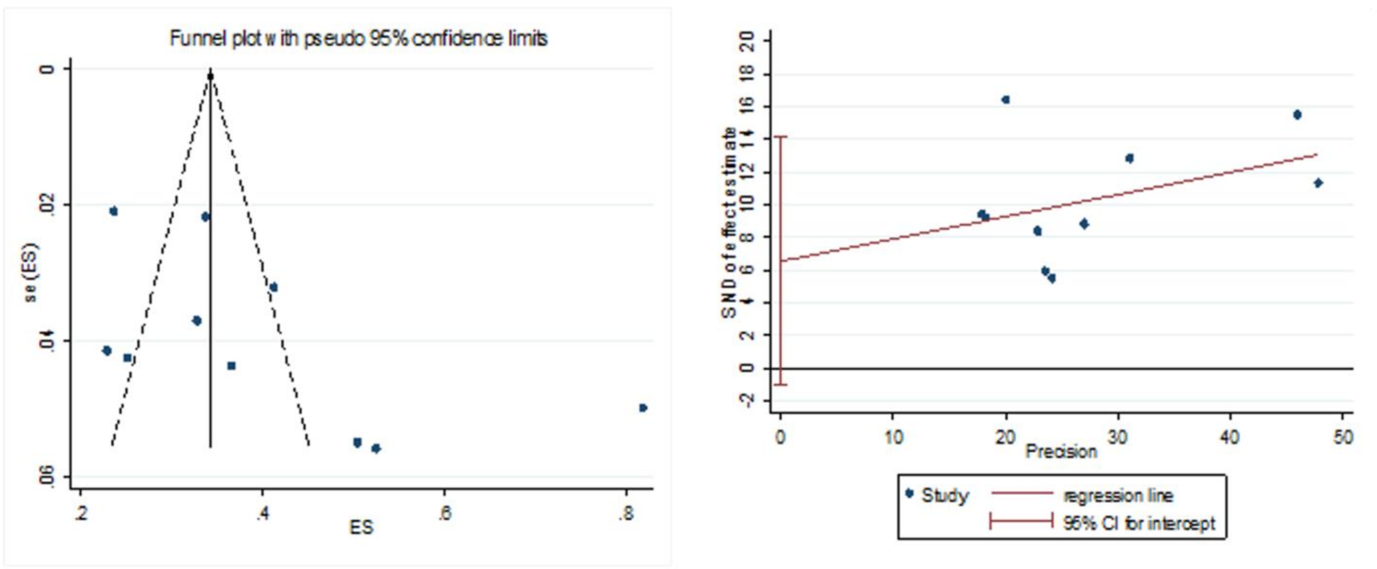

Supplementary Figure 10: Assessment of publication bias among studies reporting the rate of uptake of the mpox vaccine among the LGBTQI+ community globally. All the statistical tests performed were two-sided. a, Begg's funnel plot of included studies reporting the rate of uptake of the mpox vaccine among the LGBTQI+ community globally (n = 10 studies). b, Egger graph of included studies reporting the rate of uptake of the mpox vaccine among the LGBTQI+ community globally (p = 0.081) (n = 10 studies). ES, Effect Size. SE, Standard Error. SND, Standard Normal Distribution.

a. Begg's funnel plot for studies reporting intention to vaccinate against mpox among healthcare workers globally

b. Egger's graph for studies reporting intention to vaccinate against among healthcare workers globally (p = 0.443)

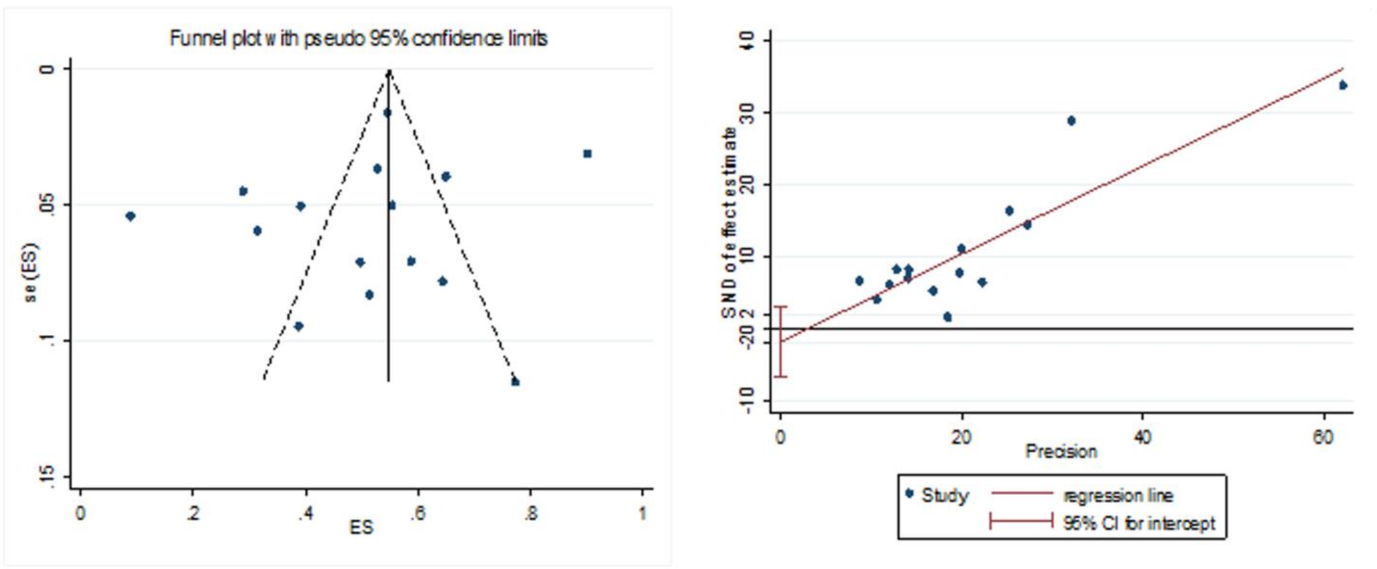

Supplementary Figure 11: Assessment of publication bias among studies reporting the rate of intention to vaccinate against the mpox among the healthcare workers globally. All the statistical tests performed were two-sided. a, Begg's funnel plot of included studies reporting the rate of intention to vaccinate against the mpox among healthcare globally (n = 15 studies). b, Egger graph of included studies reporting the rate of intention to vaccinate against the mpox among healthcare workers globally (p = 0.443) (n = 15 studies). ES, Effect Size. SE, Standard Error. SND, Standard Normal Distribution.

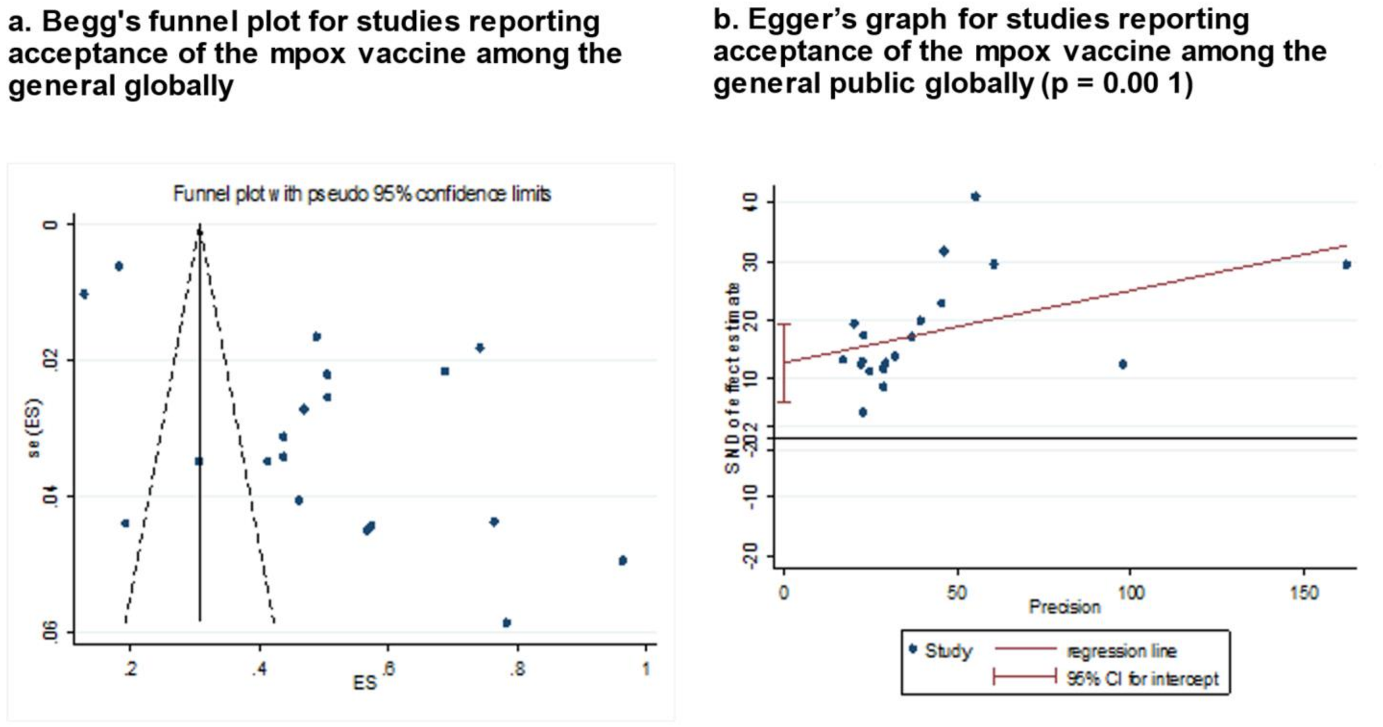

Supplementary Figure 12: Assessment of publication bias among studies reporting acceptance rate among the general public globally. All the statistical tests performed were two-sided. a, Begg's funnel plot of included studies reporting acceptance rate among the general public globally (n = 19 studies). b, Egger graph of included studies reporting acceptance rate among the general public globally (p = 0.001) (n = 19 studies). ES, Effect Size. SE, Standard Error. SND, Standard Normal Distribution.

a. Begg's funnel plot for studies reporting intention to vaccinate against the mpox among the general globally

b. Egger's graph for studies reporting intention to vaccinate against the mpox among the general public globally (p = 0.003)

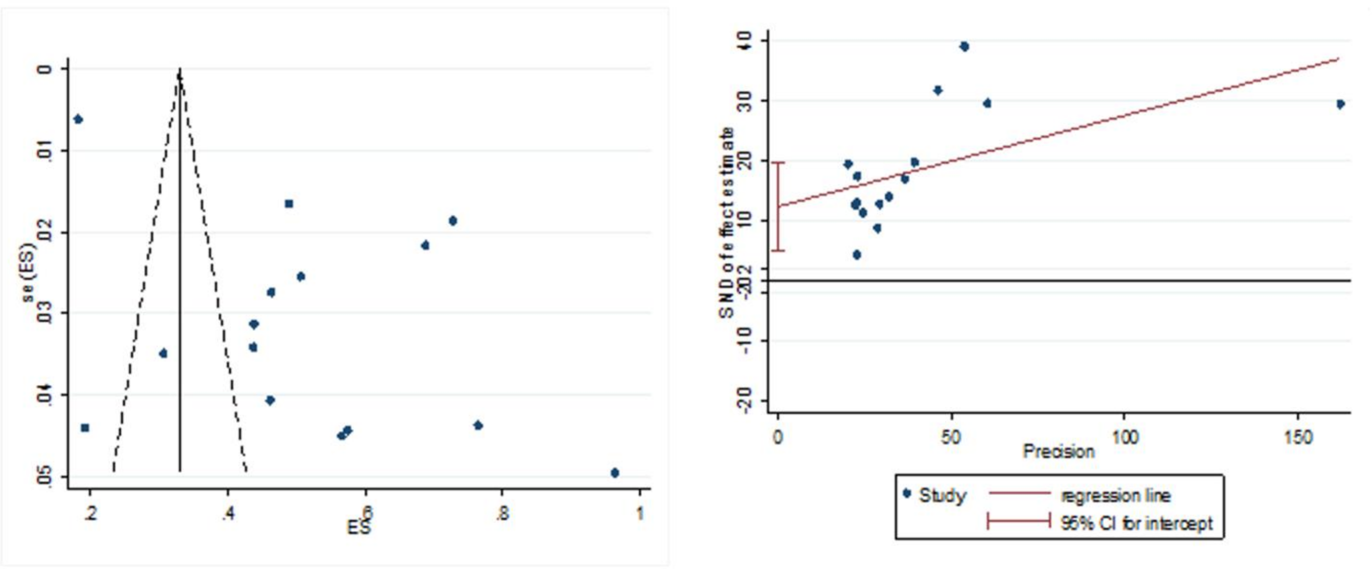

Supplementary Figure 13: Assessment of publication bias among studies reporting the rate of intention to vaccinate against the mpox among the general public globally. All the statistical tests performed were two-sided. a, Begg's funnel plot of included studies reporting acceptance rate among the general public globally (n = 15 studies). b, Egger graph of included studies reporting acceptance rate among the general public globally (p = 0.003) (n = 15 studies). ES, Effect Size. SE, Standard Error. SND, Standard Normal Distribution.

a. Begg's funnel plot for studies reporting uptake of the mpox vaccine among the general globally

b. Egger's graph for studies reporting uptake of the mpox vaccine among the general public globally (p = 0.473)

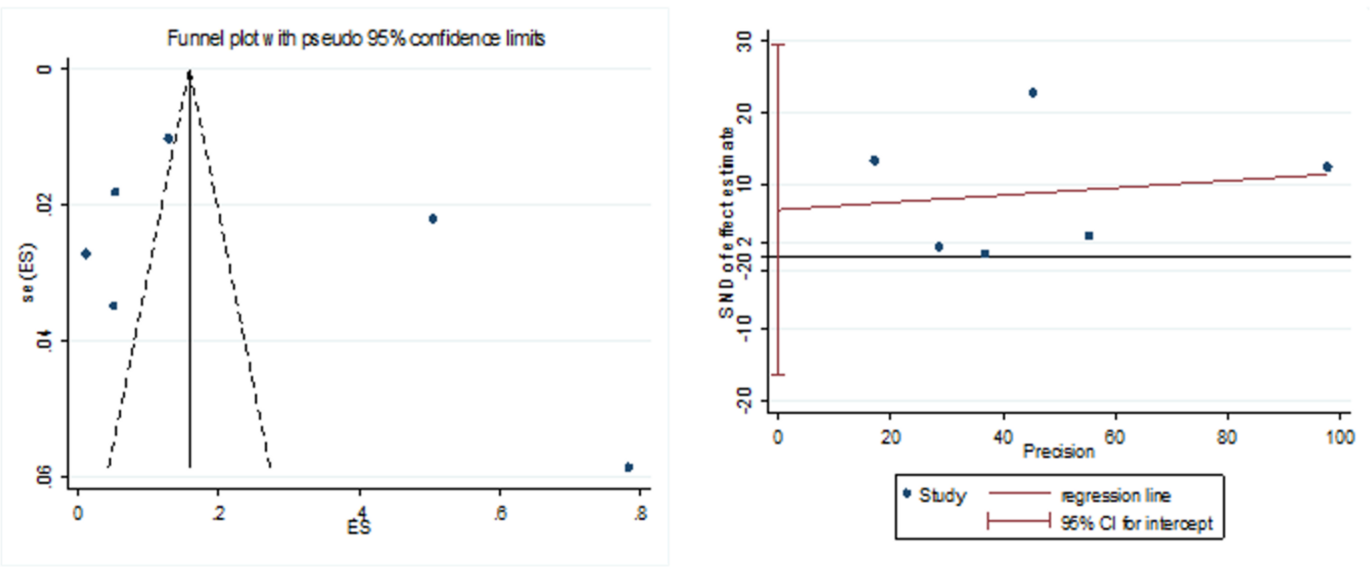

Supplementary Figure 14: Assessment of publication bias among studies reporting mpox vaccinee uptake rate among the general public globally. All the statistical tests performed were two-sided. a, Begg's funnel plot of included studies reporting uptake rate among the general public globally (n = 6 studies). b, Egger graph of included studies reporting uptake rate among the general public globally (p = 0.473) (n = 6 studies). ES, Effect Size. SE, Standard Error. SND, Standard Normal Distribution.

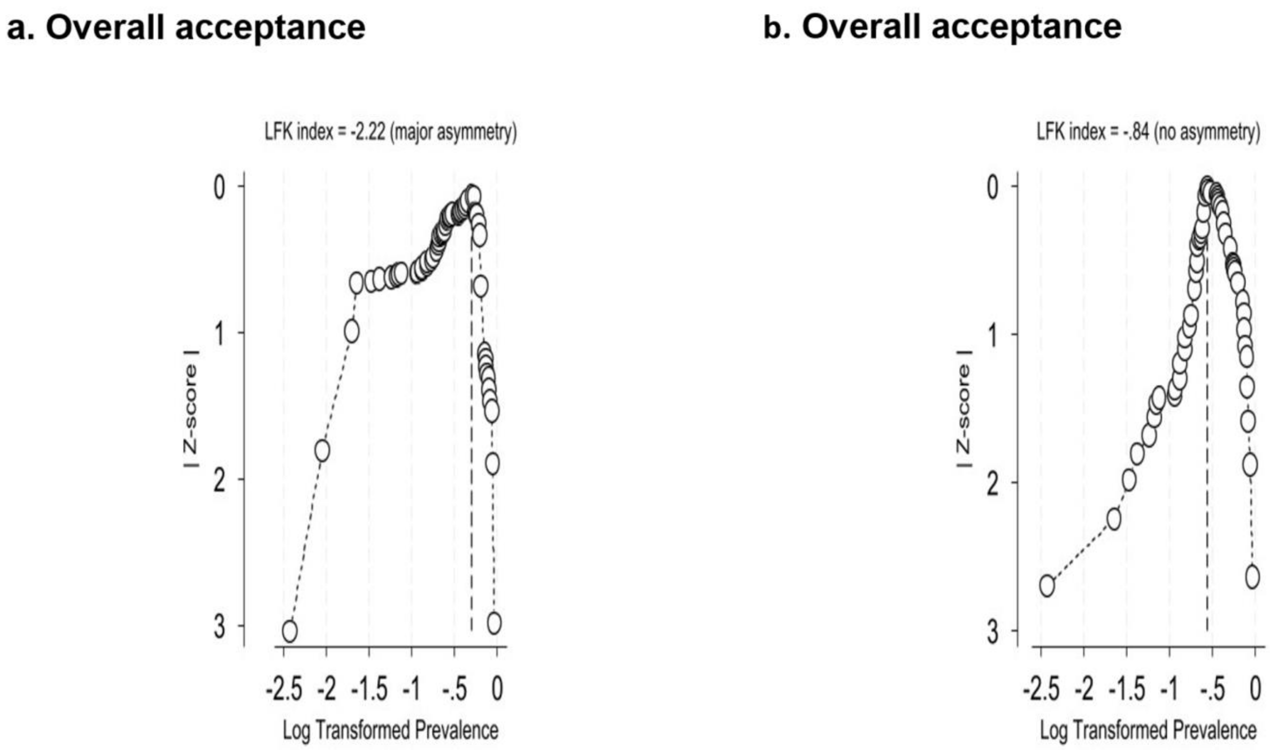

Supplementary Figure 15: Assessment of publication bias among studies reporting mpox vaccine acceptance rate globally using the Doi plot. All the statistical tests performed were two-sided. a, Doi plot of all studies reporting acceptance rate globally (n = 59 studies). b, Doi of included studies reporting acceptance globally after trimming 5 studies (Reyes-Urueña et al, Payne et al, Abd Elhafeez et al, Torres et al, Zheng b et al, Hori et al) (n = 54 studies). Each circle represents a study, while the vertical dotted lines denote the corresponding log transformed prevalence provided in the adjoining X-axis.

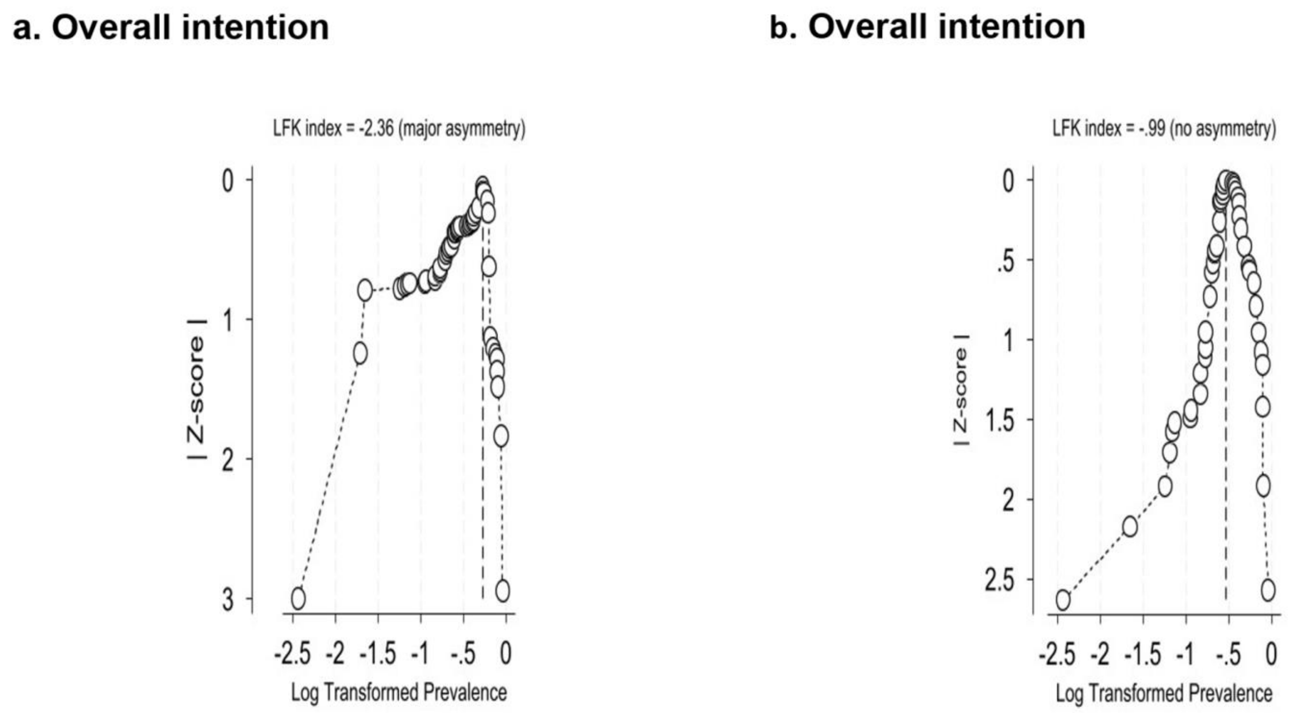

Supplementary Figure 16: Assessment of publication bias among studies reporting mpox vaccine intention rate globally using the Doi plot. All the statistical tests performed were two-sided. a, Doi plot of all studies reporting intention rate globally (n = 51 studies). b, Doi of included studies reporting intention rate globally after trimming 5 studies (Reyes-Urueña et al, Payne et al, Abd Elhafeez et al, Torres et al, Zheng b et al, Hori et al) (n = 46 studies). Each circle represents a study, while the vertical dotted lines denote the corresponding log transformed prevalence provided in the adjoining X-axis.

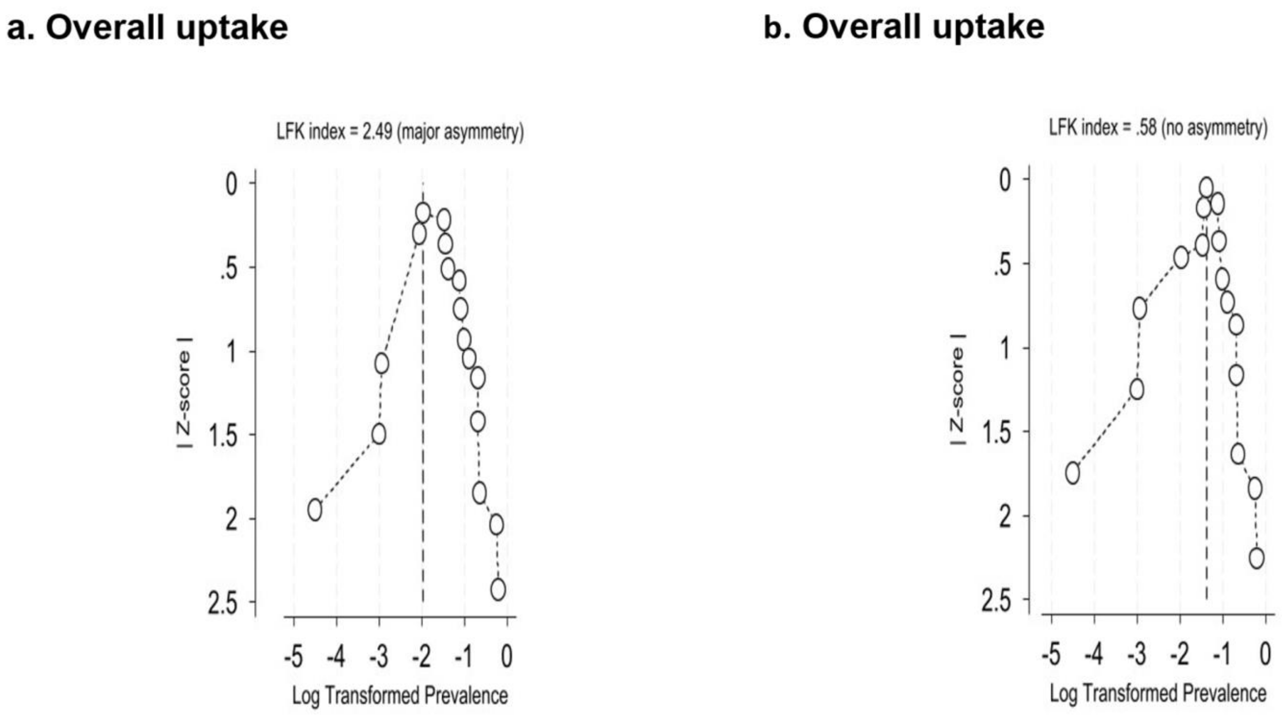

Supplementary Figure 17: Assessment of publication bias among studies reporting mpox vaccine uptake rate globally using the Doi plot. All the statistical tests performed were two-sided. a, Doi plot of all studies reporting uptake rate globally (n = 17 studies). b, Doi of included studies reporting uptake rate globally after trimming 1 study (Payne et al) (n = 16 studies). Each circle represents a study, while the vertical dotted lines denote the corresponding log transformed prevalence provided in the adjoining X-axis.

a. Uptake among the accepting group

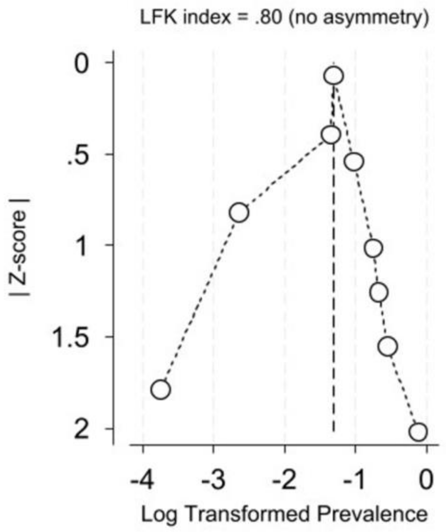

Supplementary Figure 18: Assessment of publication bias among studies reporting mpox vaccine uptake rate among accepting group rate globally using the Doi plot. All the statistical tests performed were two-sided. a, Doi plot of all studies reporting intention rate globally (n = 9 studies). Each circle represents a study, while the vertical dotted lines denote the corresponding log transformed prevalence provided in the adjoining X-axis.

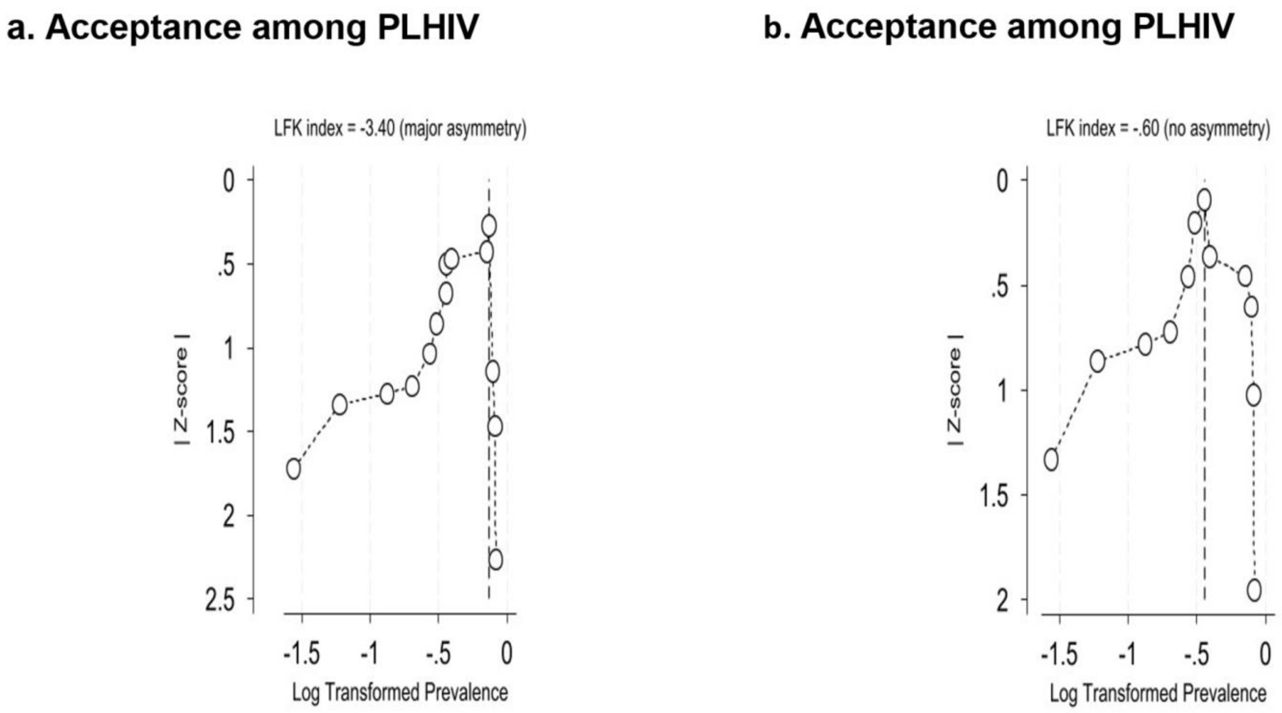

Supplementary Figure 19: Assessment of publication bias among studies reporting mpox vaccine acceptance rate among PLHIV globally using the Doi plot. All the statistical tests performed were two-sided. a, Doi plot of all studies reporting acceptance rate among PLHIV globally (n = 14 studies). b, Doi of included studies reporting acceptance among PLHIV globally after trimming 1 study (Reyes-Urueña) (n = 13 studies). Each circle represents a study, while the vertical dotted lines denote the corresponding log transformed prevalence provided in the adjoining X-axis.

a. Intention among PLHIV

b. Intention among PLHIV

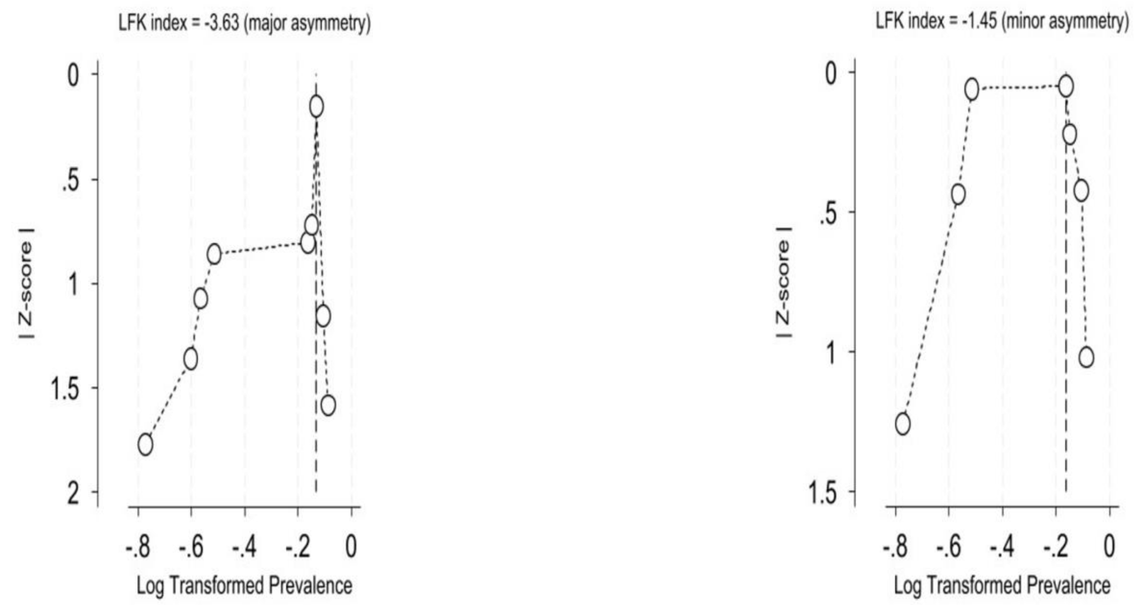

Supplementary Figure 20: Assessment of publication bias among studies reporting mpox vaccine intention rate among PLHIV globally using the Doi plot. All the statistical tests performed were two-sided. a, Doi plot of all studies reporting intention rate among PLHIV globally (n = 10 studies). b, Doi of included studies reporting intention among PLHIV globally after trimming 1 study (Reyes-Urueña) (n = 9 studies). Each circle represents a study, while the vertical dotted lines denote the corresponding log transformed prevalence provided in the adjoining X-axis.

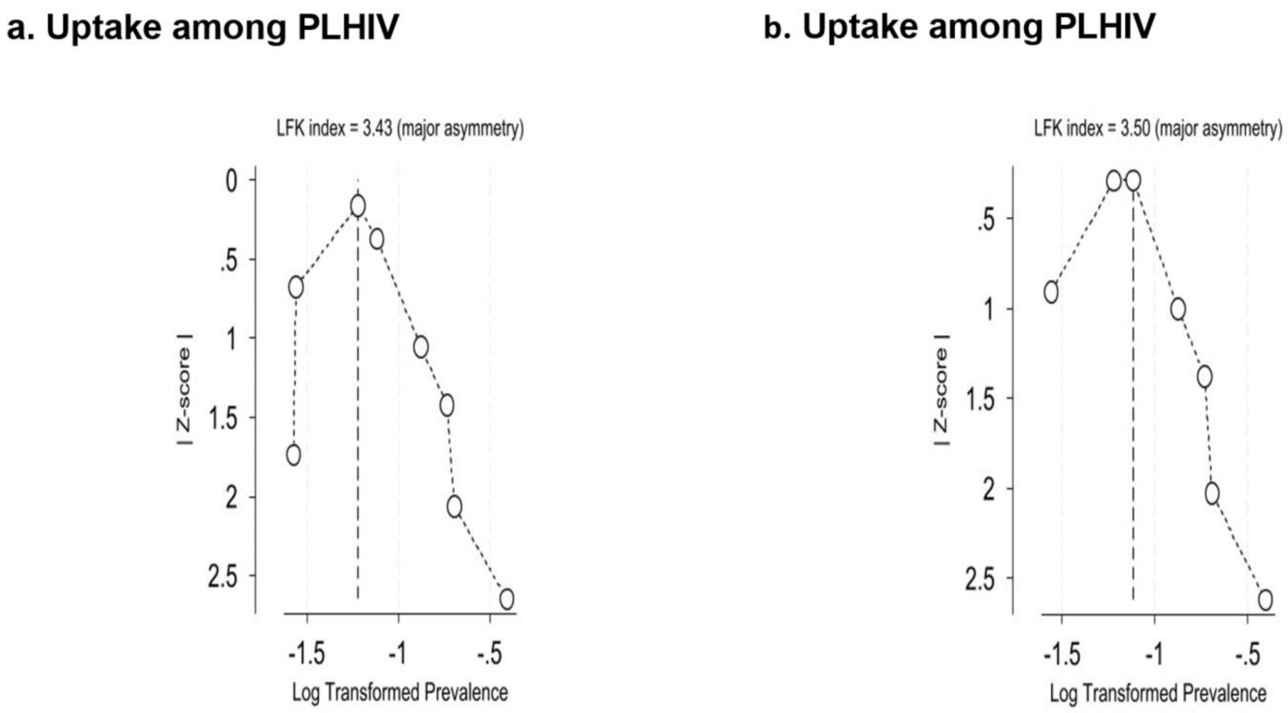

Supplementary Figure 21: Assessment of publication bias among studies reporting mpox vaccine uptake rate among PLHIV globally using the Doi plot. All the statistical tests performed were two-sided. a, Doi plot of all studies reporting uptake rate among PLHIV globally (n = 8 studies). b, Doi of included studies reporting uptake rate among PLHIV globally after trimming 1 study (Reyes-Urueña) (n = 7 studies). Each circle represents a study, while the vertical dotted lines denote the corresponding log transformed prevalence provided in the adjoining X-axis.

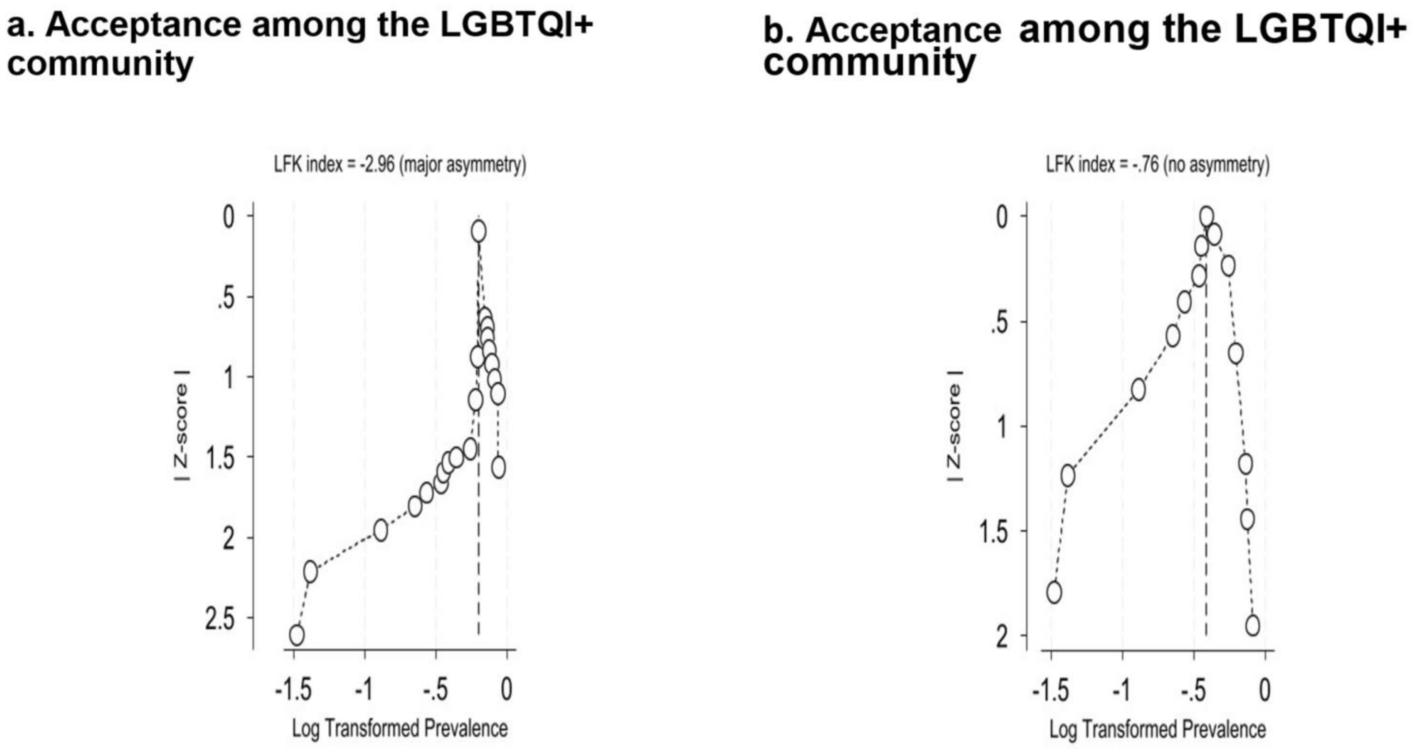

Supplementary Figure 22: Assessment of publication bias among studies reporting mpox vaccine acceptance rate among the LGBTQI+ community globally using the Doi plot. All the statistical tests performed were two-sided. a, Doi plot of all studies reporting acceptance rate among the LGBTQI+ community globally (n = 21 studies). b, Doi of included studies reporting acceptance among the LGBTQI+ community globally after trimming 7 studies (Reyes-Urueña et al, Zheng et al, MacGibbon et al, Smith et al, Paparini et al, Torres et al, Zheng b et al) (n = 14 studies). Each circle represents a study, while the vertical dotted lines denote the corresponding log transformed prevalence provided in the adjoining X-axis.

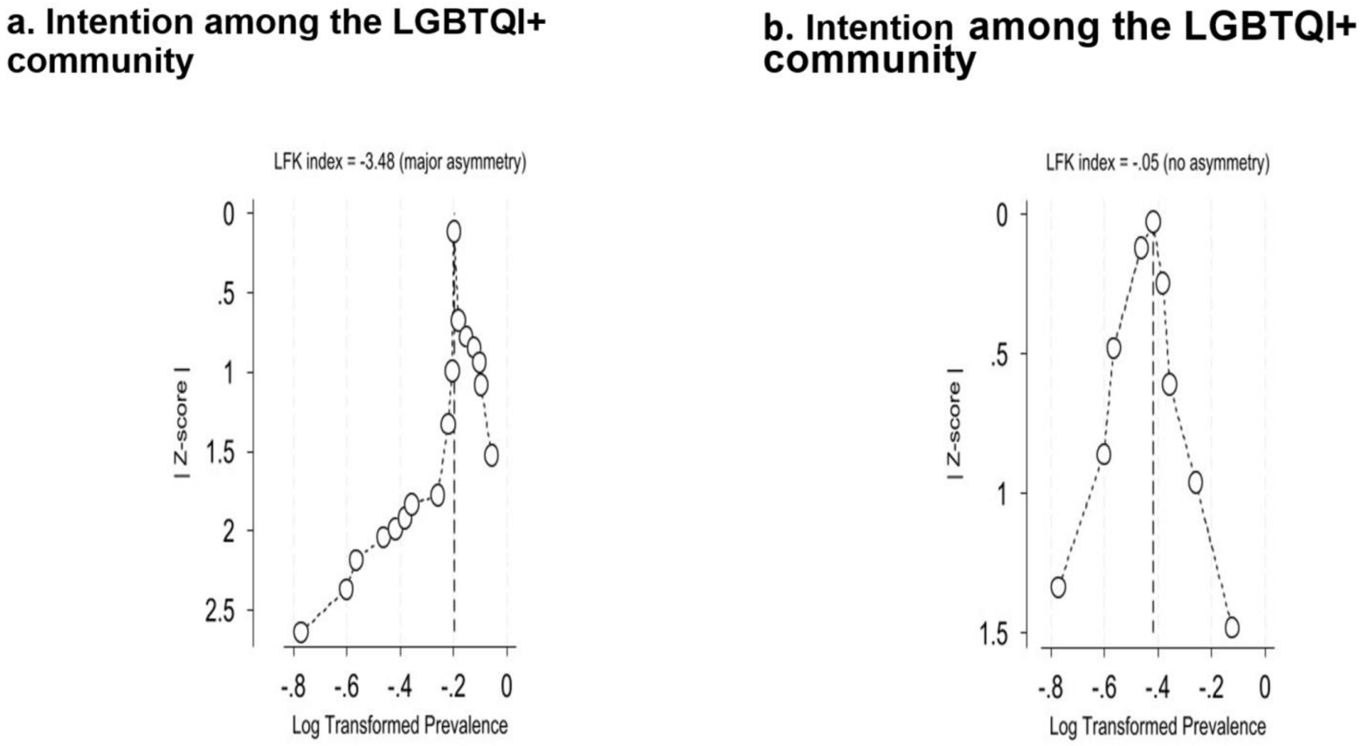

Supplementary Figure 23: Assessment of publication bias among studies reporting mpox vaccine intention rate among the LGBTQI+ community globally using the Doi plot. All the statistical tests performed were two-sided. a, Doi plot of all studies reporting intention rate among the LGBTQI+ community globally (n = 17 studies). b, Doi of included studies reporting intention among the LGBTQI+ community globally after trimming 8 studies (Reyes-Urueña et al, Zheng et al, MacGibbon et al, Smith et al, Dukers-Muijirers et al, Paparini et al, Torres et al, Zheng b et al) (n = 9 studies). Each circle represents a study, while the vertical dotted lines denote the corresponding log transformed prevalence provided in the adjoining X-axis.

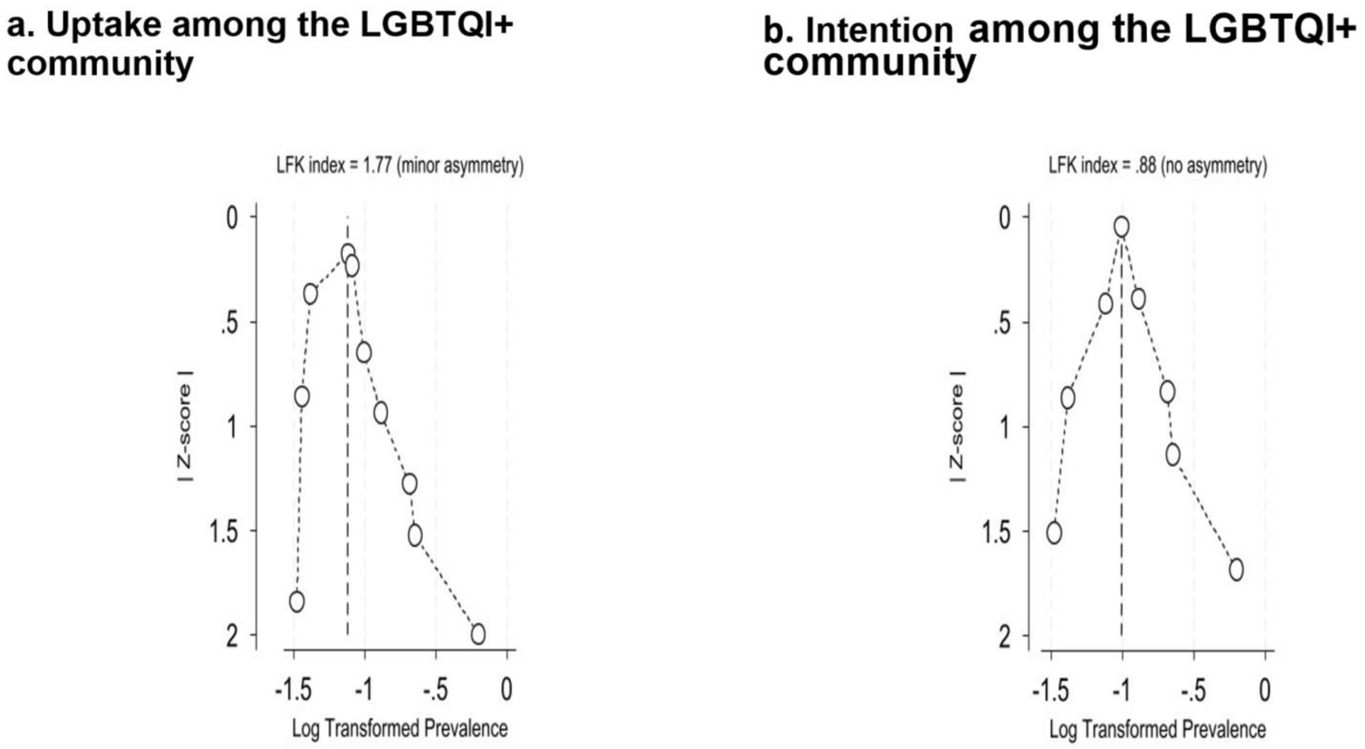

Supplementary Figure 24: Assessment of publication bias among studies reporting mpox vaccine uptake rate among the LGBTQI+ community globally using the Doi plot. All the statistical tests performed were two-sided. a, Doi plot of all studies reporting uptake rate among the LGBTQI+ community globally (n = 10 studies). b, Doi of included studies reporting uptake among the LGBTQI+ community globally after trimming 2 studies (MacGibbon et al, Smith et al) (n = 7 studies). Each circle represents a study, while the vertical dotted lines denote the corresponding log transformed prevalence provided in the adjoining X-axis.

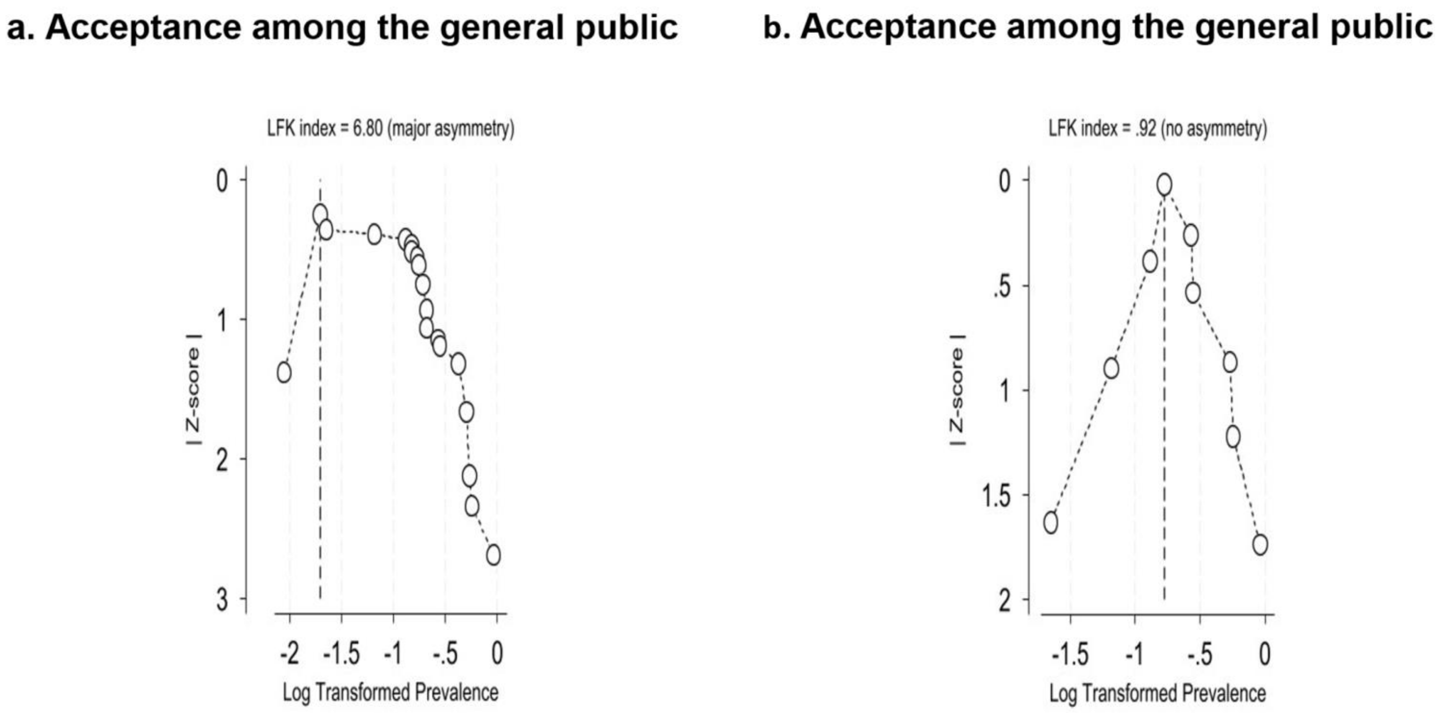

Supplementary Figure 25: Assessment of publication bias among studies reporting mpox vaccine acceptance rate among the general public globally using the Doi plot. All the statistical tests performed were two-sided. a, Doi plot of all studies reporting acceptance rate among the general public globally (n = 20 studies). b, Doi of included studies reporting acceptance among the general public globally after trimming 10 studies (Gallè et al, Winters et al, Payne et al, Temsah et al, Swed et al, Smith et al, Wang et al, Meo et al, Sagy et al, Hori et al) (n = 10 studies). Each circle represents a study, while the vertical dotted lines denote the corresponding log transformed prevalence provided in the adjoining X-axis.

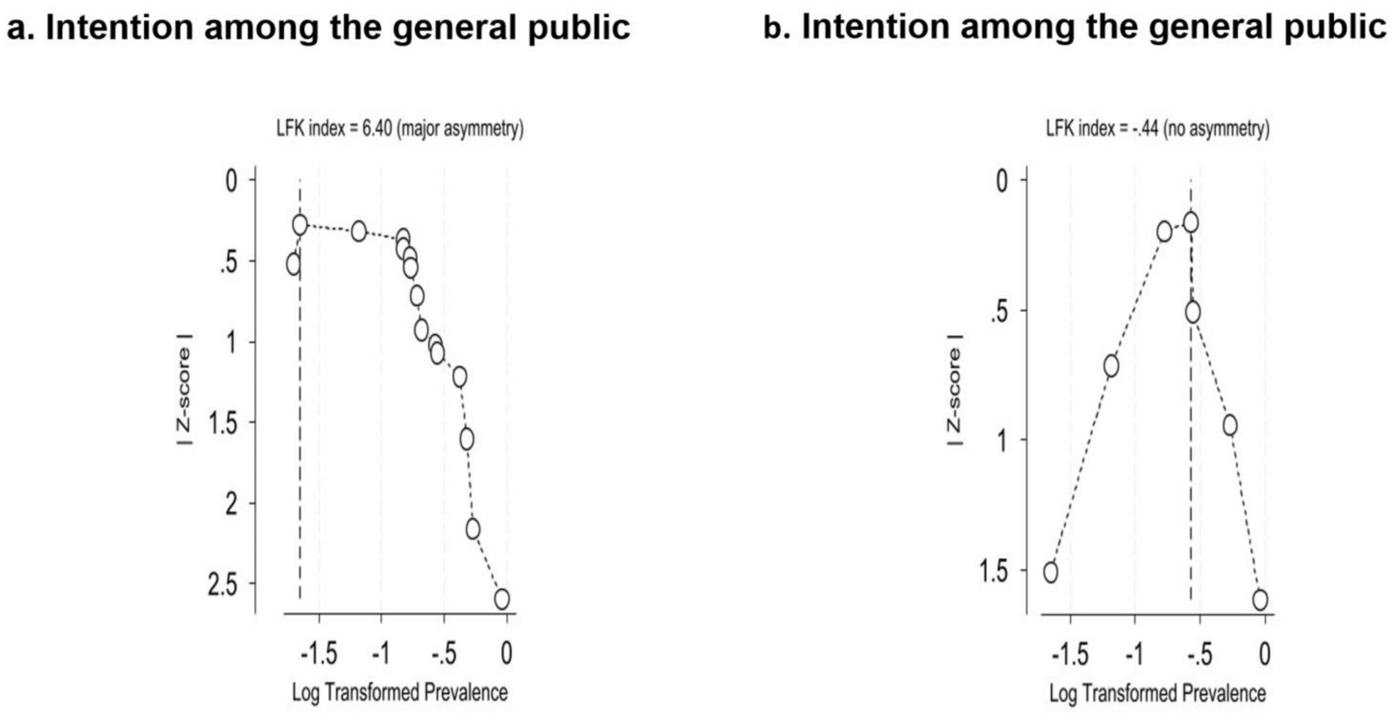

Supplementary Figure 26: Assessment of publication bias among studies reporting mpox vaccine intention rate among the general public globally using the Doi plot. All the statistical tests performed were two-sided. a, Doi plot of all studies reporting intention rate among the general public globally (n = 15 studies). b, Doi of included studies reporting intention among the general public globally after trimming 7 studies (Gallè et al, Temsah et al, Swed et al, Smith et al, Wang et al, Meo et al, Hori et al) (n = 8 studies). Each circle represents a study, while the vertical dotted lines denote the corresponding log transformed prevalence provided in the adjoining X-axis.

a. Uptake among the general public

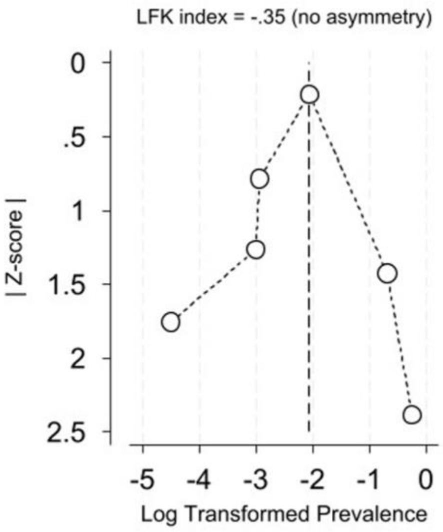

Supplementary Figure 27: Assessment of publication bias among studies reporting mpox vaccine uptake rate among the general public globally using the Doi plot. All the statistical tests performed were two-sided. a, Doi plot of all studies reporting uptake rate among the general public globally (n = 6 studies). Each circle represents a study, while the vertical dotted lines denote the corresponding log transformed prevalence provided in the adjoining X-axis.

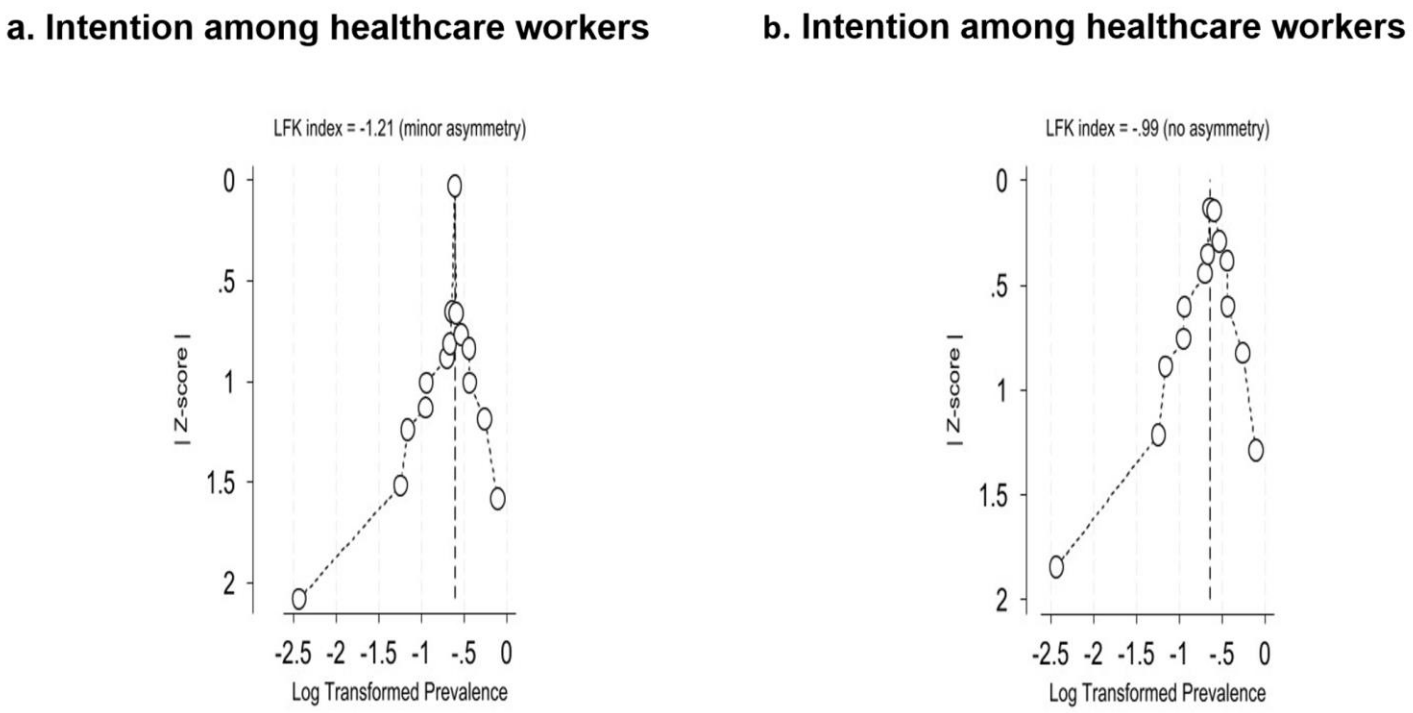

Supplementary Figure 28: Assessment of publication bias among studies reporting mpox vaccine intention rate among healthcare workers globally using the Doi plot. All the statistical tests performed were two-sided. a, Doi plot of all studies reporting intention rate among healthcare workers globally (n = 15 studies). b, Doi of included studies reporting intention among healthcare workers globally after trimming 1 study (Swed et al) (n = 14 studies). Each circle represents a study, while the vertical dotted lines denote the corresponding log transformed prevalence provided in the adjoining X-axis.

Supplementary Table 2: Sensitivity Analysis for LFK Index

Outcome	LFK Index Before Trimming	LFK index After Trimming	Studies Trimmed	Prevalence Before Trimming (%)	Prevalence After Trimming (%)	Absolute Difference in Prevalence
**Overall**						
Acceptance	-2.22	-0.84	Reyes-Urueña, Payne, Abd Elhafeez, Torres, Zheng b, Hori	59.7	59.5	0.2
Uptake	2.49	0.58	Payne	30.9	32.2	1.3
Intention	-2.36	-0.99	Reyes-Urueña, Abd Elhafeez, Torres, Zheng b, Hori	60.9	59.6	1.3
Uptake in accepting group	0.8			36.1		
**LGBTQI+ Community**						
Acceptance	-2.96	-0.76	Reyes-Urueña, Zheng, MacGibbon, Smith, Paparini, Torres, Zheng b	73.6	64.7	8.9
Uptake	1.77	0.88	MacGibbon, Smith	39.8	42.8	-3.0
Intention	-3.48	-0.05	Reyes-Urueña, Zheng, MacGibbon, Smith, Dukers-Muijirers, Paparini, Torres, Zheng b	77.1	66.3	10.8
**PLHIV**						
Acceptance	-3.4	-0.6	Reyes-Urueña	66.4	64.4	2.0
Uptake	3.43	3.5	Reyes-Urueña	35.7	38.4	2.7
Intention	-3.63	-1.45	Reyes-Urueña	75.0	75.5	0.5
**Healthcare Workers**						
Acceptance	-1.21	-0.99	Swed	51.0	50.8	0.2
Uptake¶						
Intention	-1.21	-0.99	Swed	51.0	50.8	0.2
**General Public**						
Acceptance	6.8	0.92	Gallè, Winters, Payne, Temsah, Swed, Smith, Wang, Meo, Sagy, Hori	50.9	57.2	6.3
Uptake	-0.35			20.2		
Intention	6.4	-0.44	Gallè, Temsah, Swed, Smith, Wang, Meo, Hori	52.3	56.2	3.9

¶ - Not reported by any study

Results of a meta-analysis of mpox vaccine acceptance and uptake (Supplementary Figures 29 - 57)

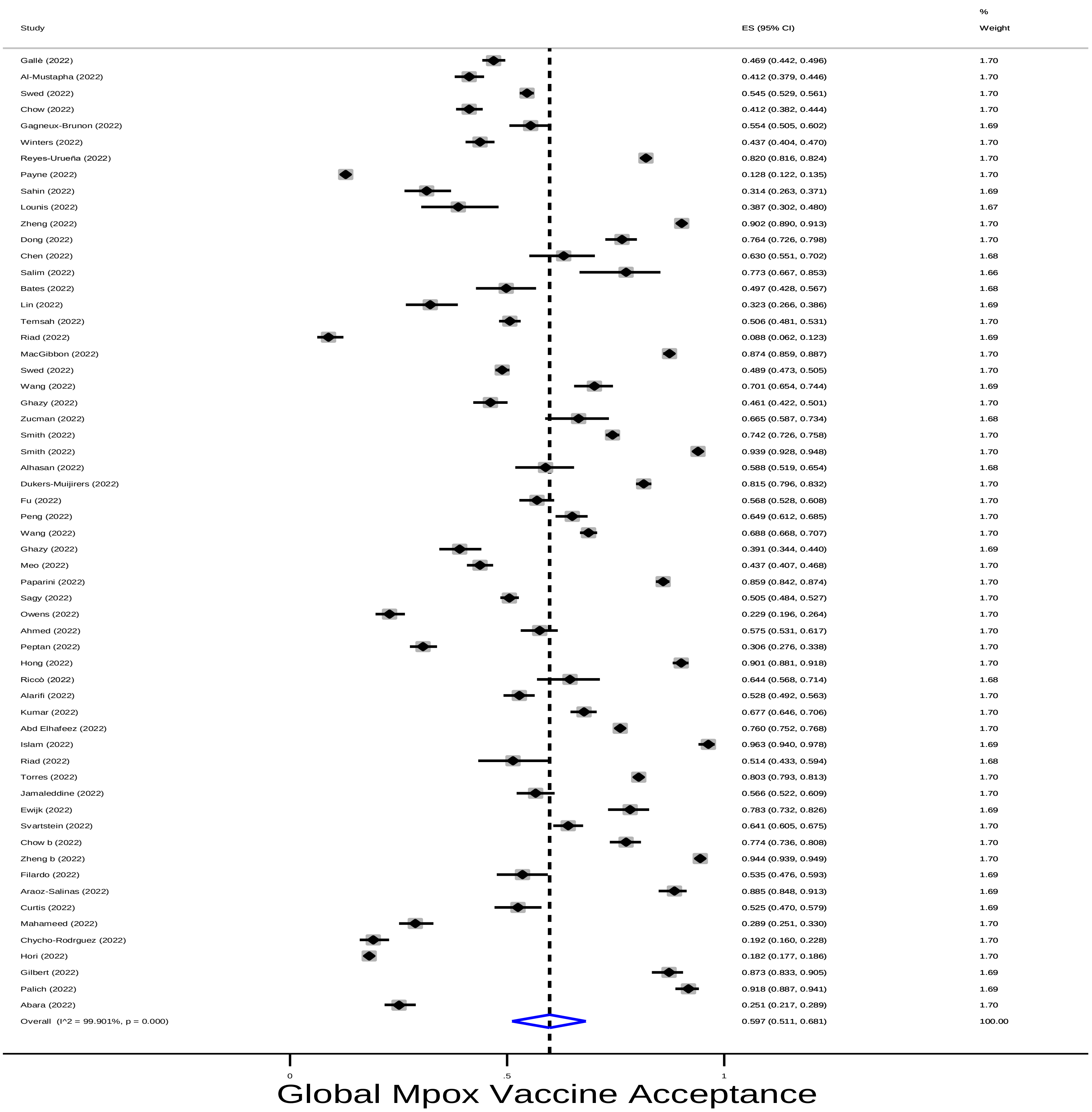

Supplementary Figure 29: Forest plots of the results of random-effects model meta-analysis of the prevalence (%) of mpox vaccine acceptance globally (n = 59 studies) pooled using inverse variance weights. Heterogeneity (I^2) = 99.90%. Each black-colored solid square represents the effect size of each characteristic, while the ends of the adjoining horizontal lines represent lower

(left) and upper (right) confidence intervals. The blue-colored hollow diamond at the bottom denotes the overall estimated effect size and the 95% CI. All statistics were based on a two-sided t-test. ES, Effect Size.

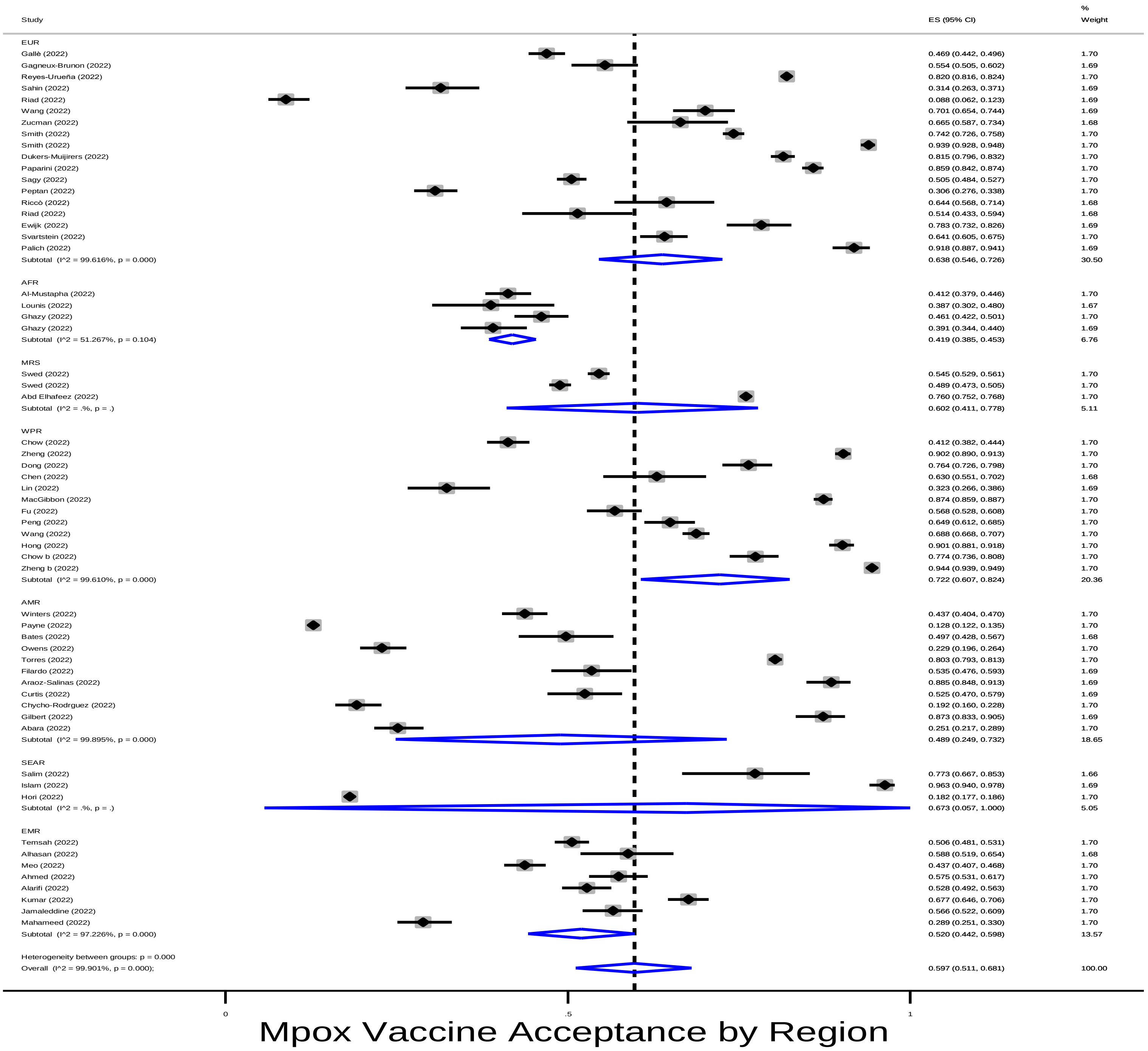

Supplementary Figure 30: Forest plots of the results of random-effects model meta-analysis of the prevalence (%) of mpox vaccine acceptance according to the six WHO regions (AFR, Africa [n = 4 studies]; AMR, Region of the Americas [n = 11 studies]; EMR, Eastern Mediterranean Region [n = 8 studies]; EUR, European Region [n = 18 studies]; SEAR South East Asia Region [n = 3 studies]; WPR, Western Pacific Region [n = 12 studies]; MRS, Multiregional Studies [n = 3 studies]) pooled using inverse variance weights. Heterogeneity (I^2) = AFR (51.23%), AMR (99.90%), EMR (97.23%), EUR (99.62), SEAR (.%), WPR (99.6%), MRS (.%) Each black-colored solid square represents the effect size of each characteristic, while the ends of the adjoining

horizontal lines represent lower (left) and upper (right) confidence intervals. The blue-colored hollow diamond at the bottom denotes the overall estimated effect size and the 95% CI. All statistics were based on a two-sided t-test. ES, Effect Size.

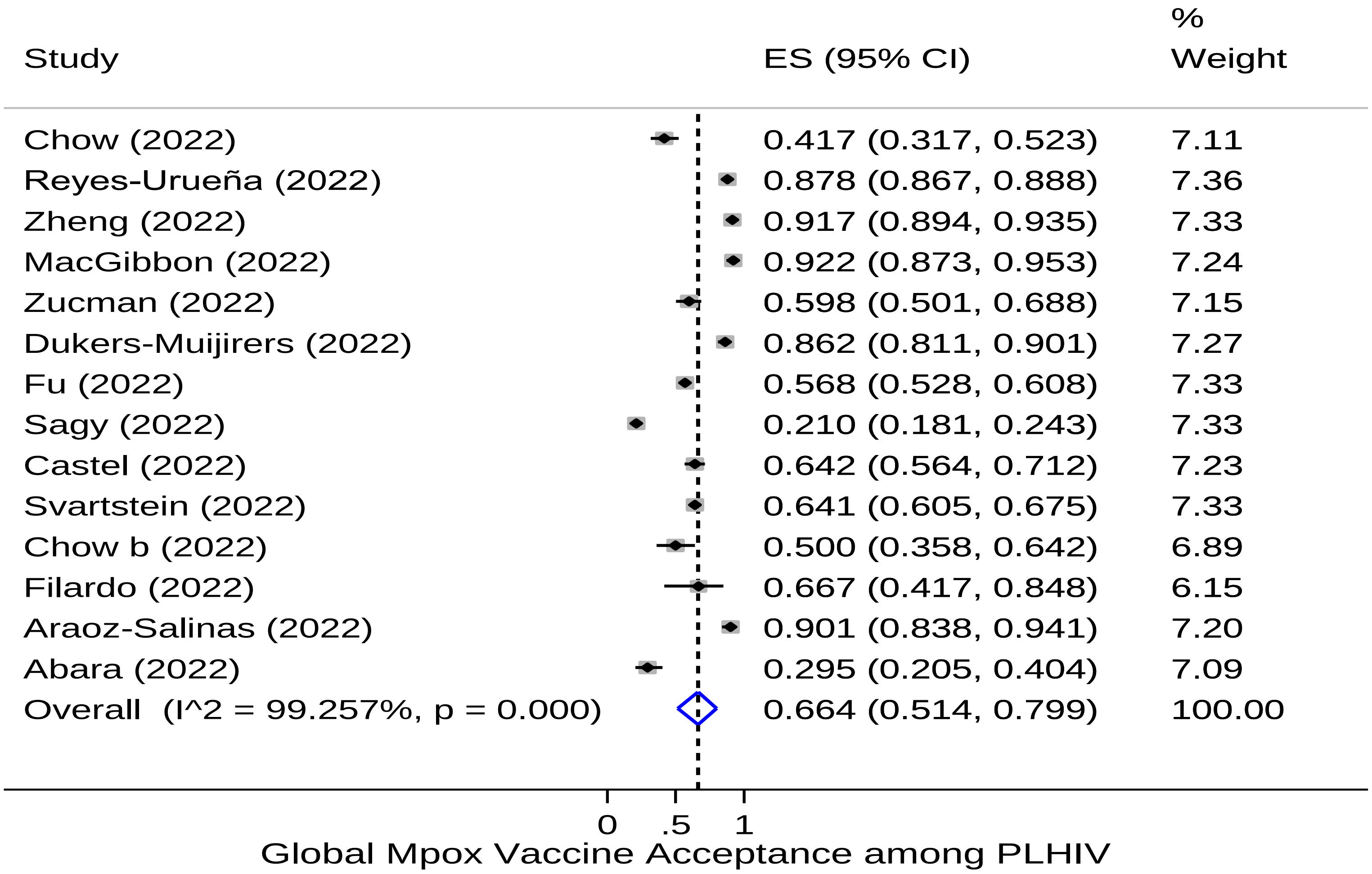

Supplementary Figure 31: Forest plots of the results of random-effects model meta-analysis of the prevalence (%) of mpox vaccine acceptance among PLHIV globally (n = 14 studies) pooled using inverse variance weights. Heterogeneity (I^2) = 99.26%. Each black-colored solid square represents the effect size of each characteristic, while the ends of the adjoining horizontal lines represent lower (left) and upper (right) confidence intervals. The blue-colored hollow diamond at the bottom denotes the overall estimated effect size and the 95% CI. All statistics were based on a two-sided t-test. ES, Effect Size.

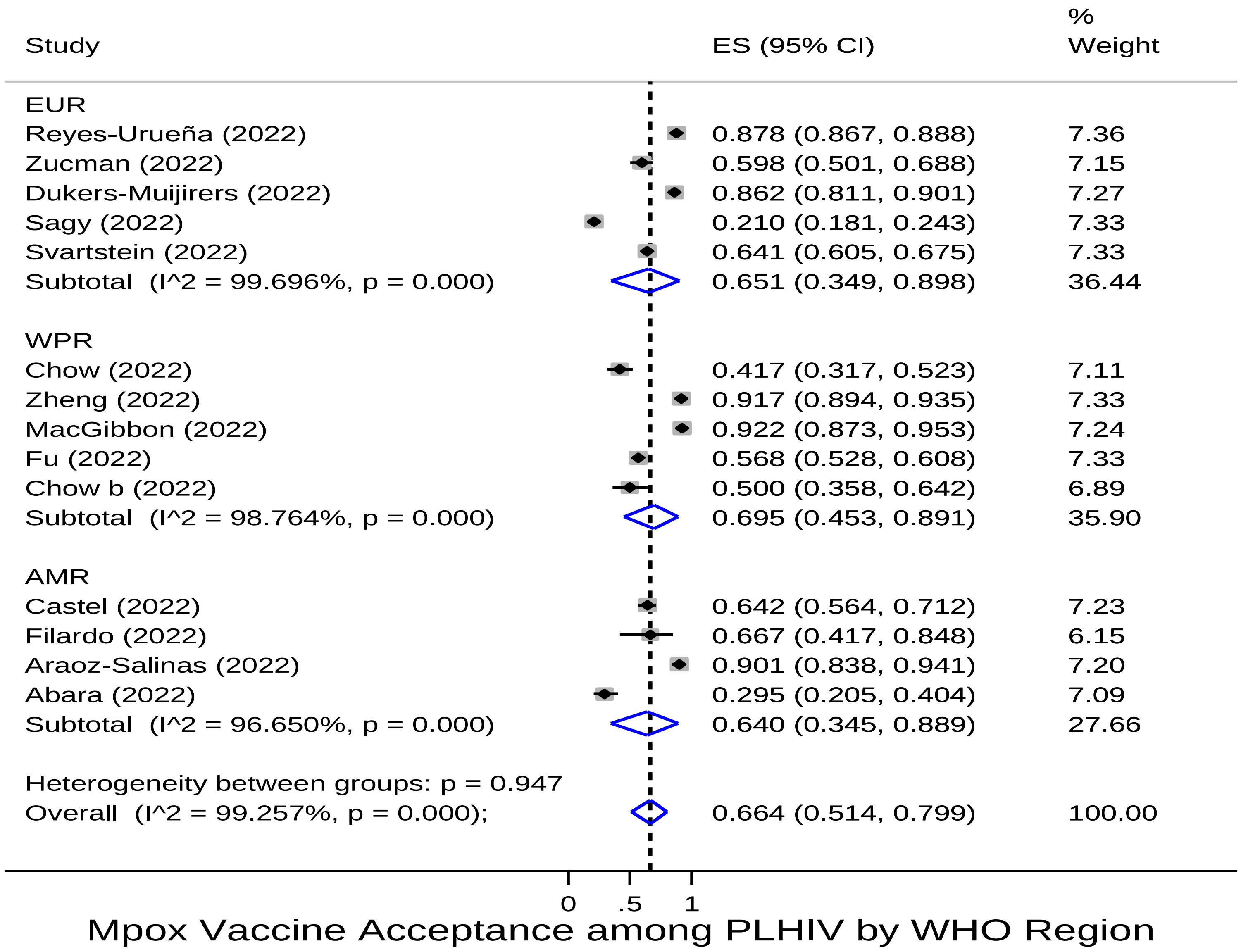

Supplementary Figure 32: Forest plots of the results of random-effects model meta-analysis of the prevalence (%) of mpox vaccine acceptance according to WHO region (AMR, Region of the Americas [n = 4 studies]; EMR, EUR, European Region [n = 5 studies]; WPR, Western Pacific Region [n = 5 studies]) pooled using inverse variance weights. Heterogeneity (I^2) = AMR (96.65%), EUR (99.70%), WPR (98.76%). Each black-colored solid square represents the effect size of each characteristic, while the ends of the adjoining horizontal lines represent lower (left) and upper (right) confidence intervals. The blue-colored hollow diamond at the bottom denotes the overall estimated effect size and the 95% CI. All statistics were based on a two-sided t-test. ES, Effect Size.

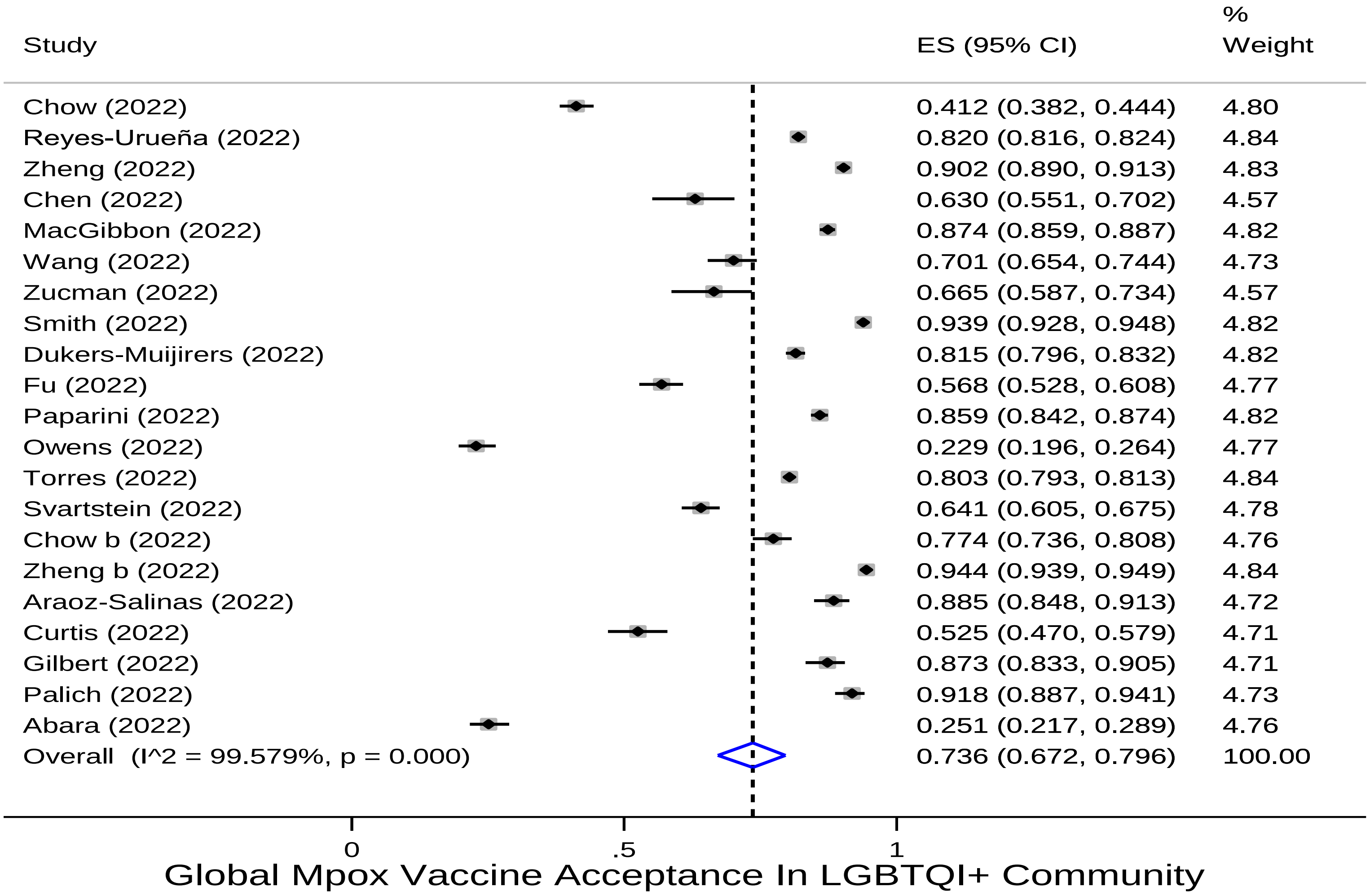

Supplementary Figure 33: Forest plots of the results of random-effects model meta-analysis of the prevalence (%) of mpox vaccine acceptance among the LGBTQI+ community globally (n = 21 studies) pooled using inverse variance weights. Heterogeneity (I^2) = 99.58%. Each black-colored solid square represents the effect size of each characteristic, while the ends of the adjoining horizontal lines represent lower (left) and upper (right) confidence intervals. The blue-colored hollow diamond at the bottom denotes the overall estimated effect size and the 95% CI. All statistics were based on a two-sided t-test. ES, Effect Size.

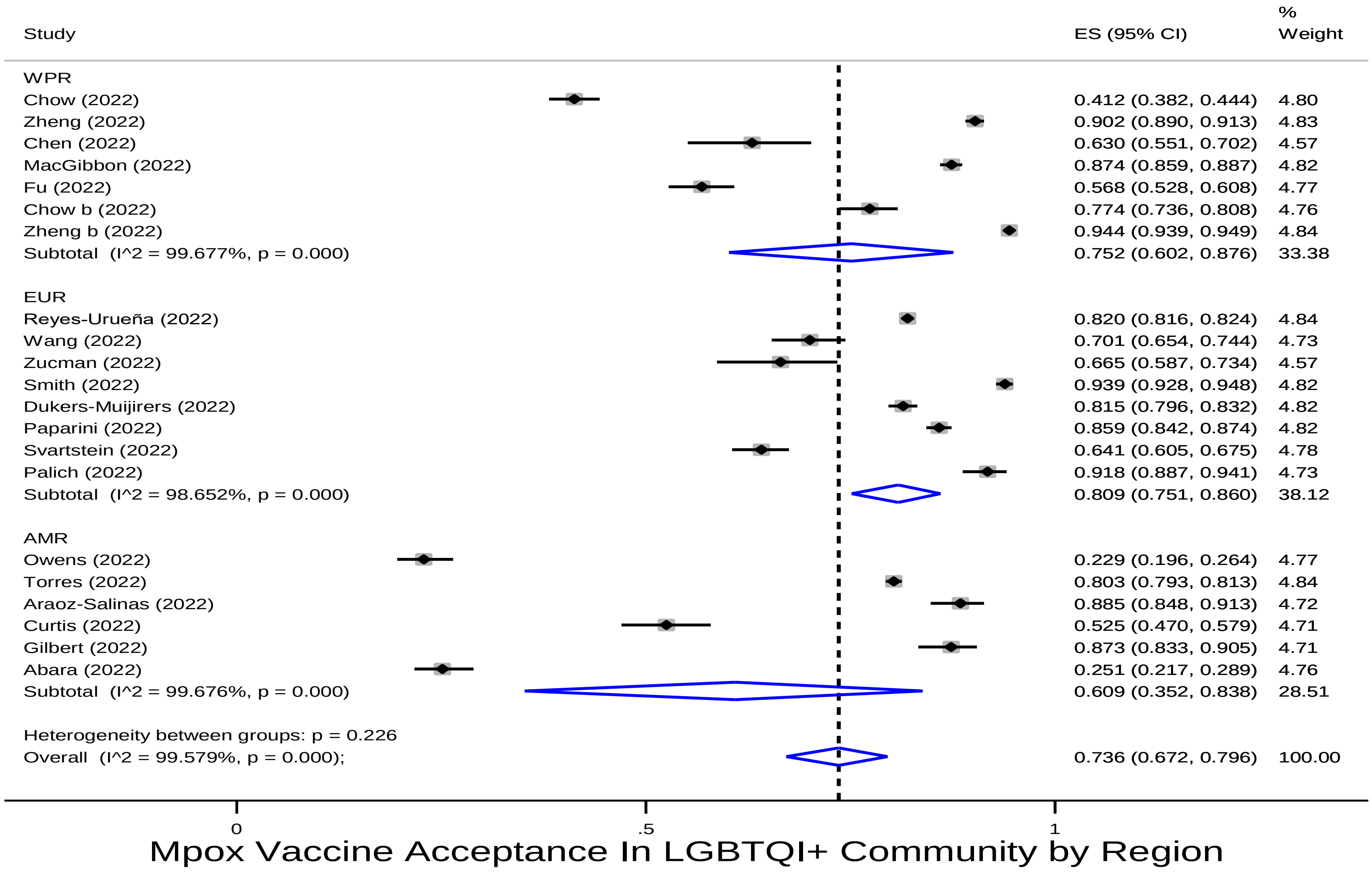

Supplementary Figure 34: Forest plots of the results of random-effects model meta-analysis of the prevalence (%) of mpox vaccine uptake according to WHO region (AMR, Region of the Americas [n = 6 studies]; EMR, EUR, European Region [n = 8 studies]; WPR, Western Pacific Region [n = 7 studies]) pooled using inverse variance weights. Heterogeneity (I^2) = AMR (99.68%), EUR (98.65%), WPR (99.68%). Each black-colored solid square represents the effect size of each characteristic, while the ends of the adjoining horizontal lines represent lower (left) and upper (right) confidence intervals. The blue-colored hollow diamond at the bottom denotes the overall estimated effect size and the 95% CI. All statistics were based on a two-sided t-test. ES, Effect Size.

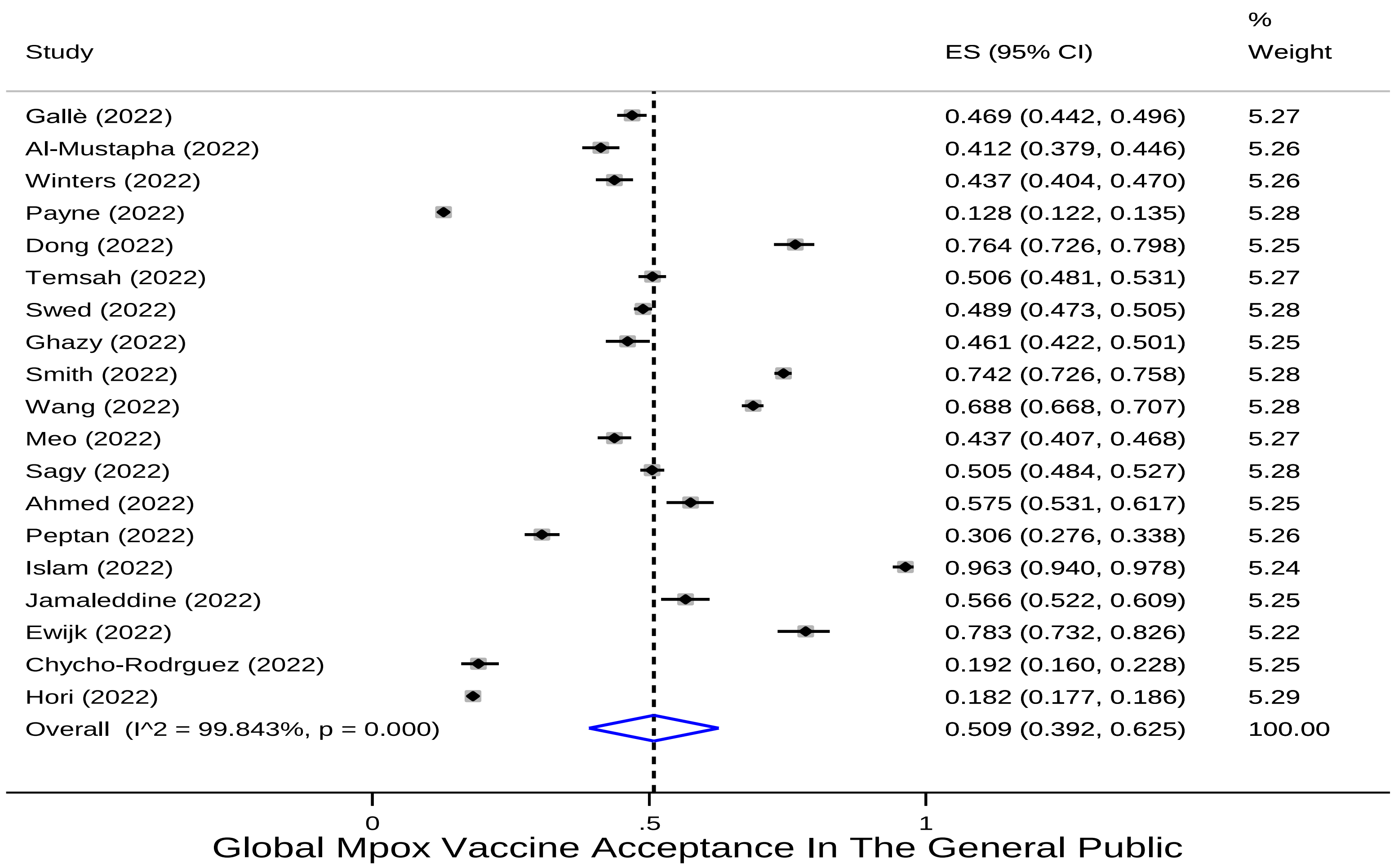

Supplementary Figure 35: Forest plots of the results of random-effects model meta-analysis of the prevalence (%) of mpox vaccine acceptance among the general public globally (n = 15 studies) pooled using inverse variance weights. Heterogeneity (I^2) = 99.01%. Each black-colored solid square represents the effect size of each characteristic, while the ends of the adjoining horizontal lines represent lower (left) and upper (right) confidence intervals. The blue-colored hollow diamond at the bottom denotes the overall estimated effect size and the 95% CI. All statistics were based on a two-sided t-test. ES, Effect Size.

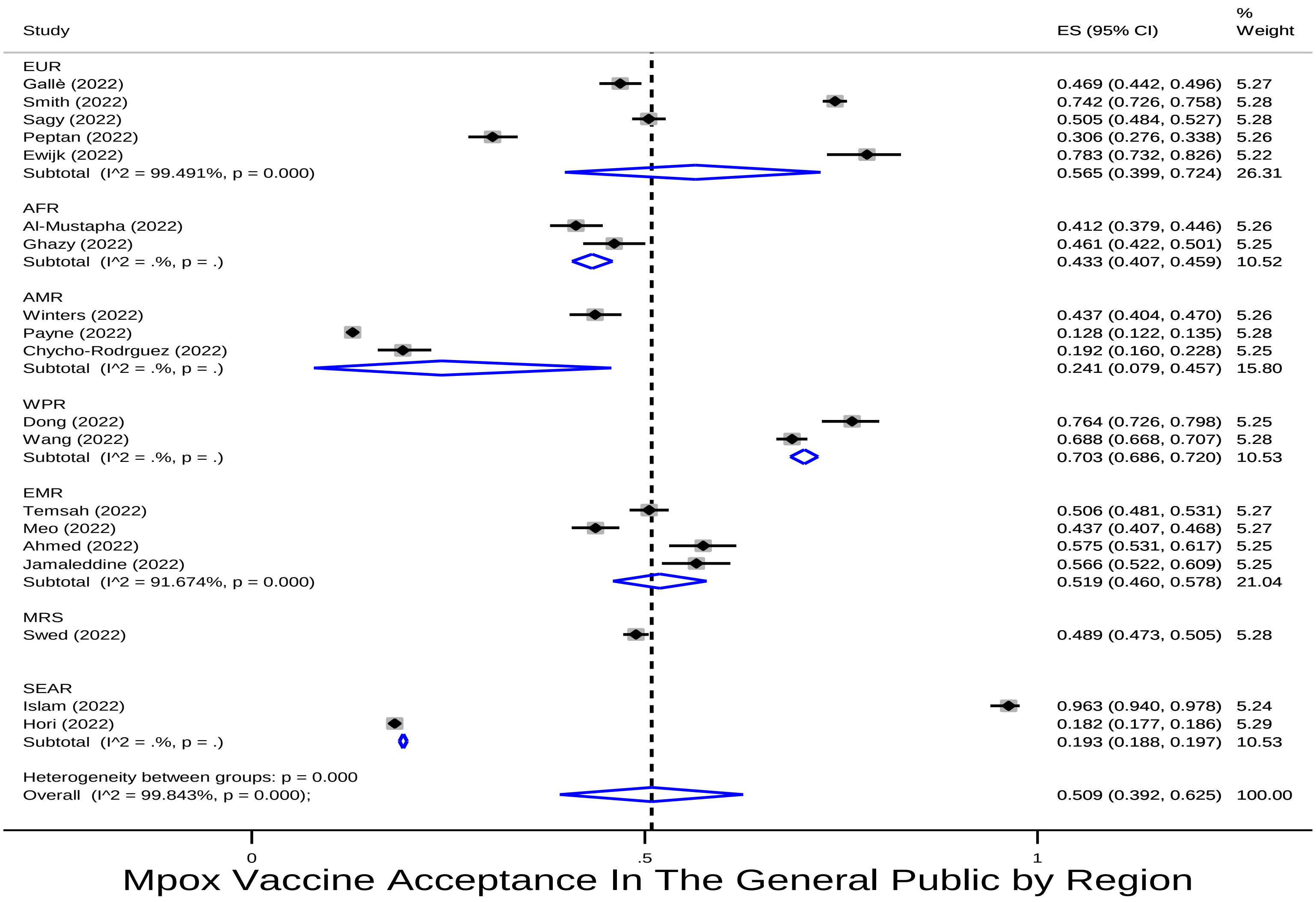

Supplementary Figure 36: Forest plots of the results of random-effects model meta-analysis of the prevalence (%) of mpox vaccine acceptance among the general public according to WHO region (AFR, Africa [n = 2 studies]; AMR, Region of the Americas [n = 3 studies]; EMR, Eastern Mediterranean Region [n = 4 studies]; EUR, European Region [n = 5 studies]; SEAR South East Asia Region [n = 2 studies]; WPR, Western Pacific Region [n = 2 studies]; MRS, Multiregional Studies [n = 1 study]) pooled using inverse variance weights. Heterogeneity (I^2) = AFR (.%), AMR (.%), EMR (96.74%), EUR (99.49%), SEAR (.%), WPR (.%), and MRS (.%). Each black-colored solid square represents the effect size of each characteristic, while the ends of the adjoining horizontal lines represent lower (left) and upper (right) confidence intervals. The blue-colored hollow diamond at the bottom denotes the overall estimated effect size and the 95% CI. All statistics were based on a two-sided t-test. ES, Effect Size.

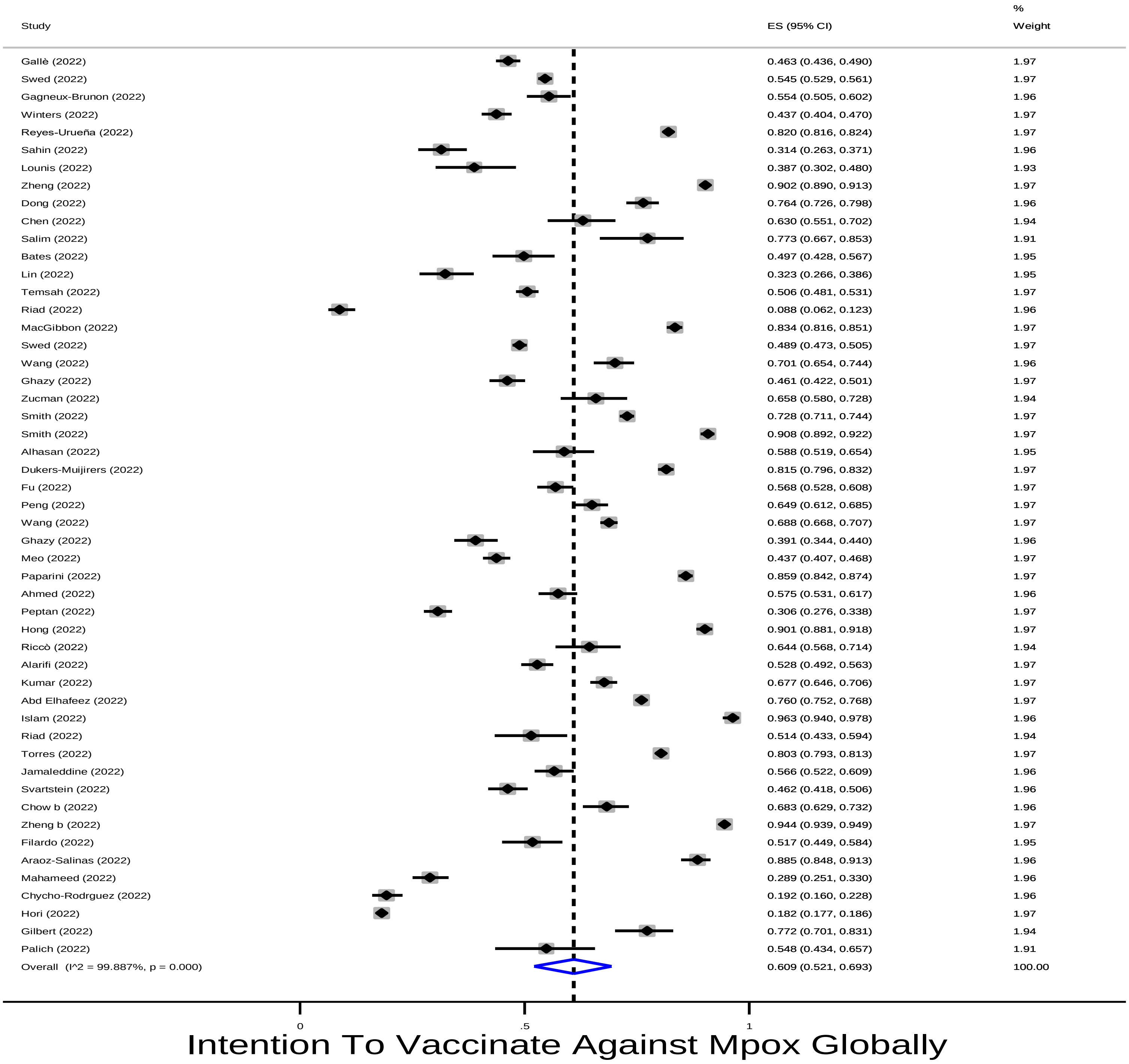

Supplementary Figure 37: Forest plots of the results of random-effects model meta-analysis of the prevalence (%) of intention to vaccinate against mpox globally (n = 51 studies) pooled using inverse variance weights. Heterogeneity (I^2) = 99.89%. Each black-colored solid square represents the effect size of each characteristic, while the ends of the adjoining horizontal lines represent lower (left) and upper (right) confidence intervals. The blue-colored hollow diamond at the bottom denotes the overall estimated effect size and the 95% CI. All statistics were based on a two-sided t-test. ES, Effect Size.

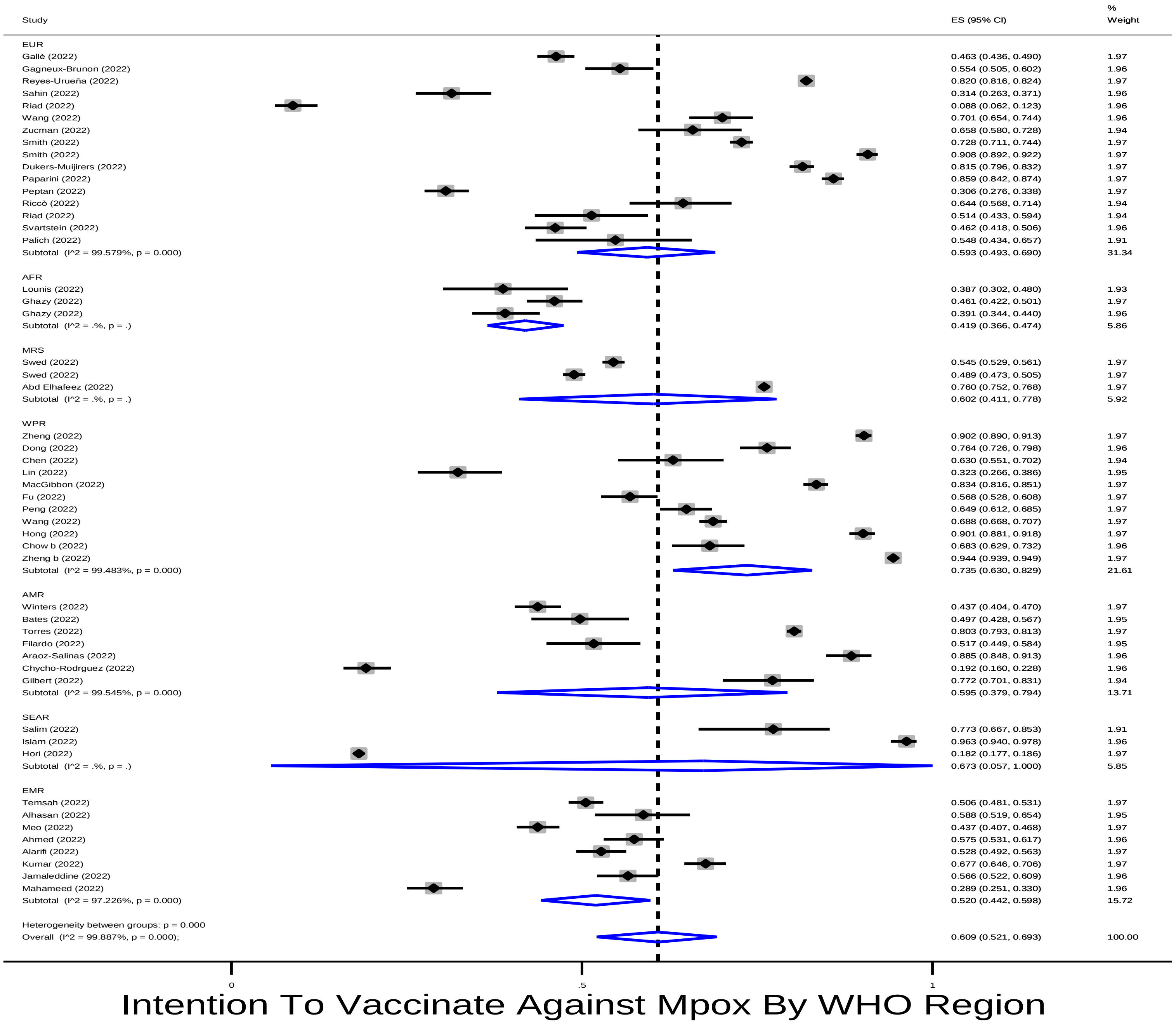

Supplementary Figure 38: Forest plots of the results of random-effects model meta-analysis of the prevalence (%) of mpox vaccine intention according to the six WHO regions (AFR, Africa [n = 3 studies]; AMR, Region of the Americas [n = 7 studies]; EMR, Eastern Mediterranean Region [n = 8 studies]; EUR, European Region [n = 15 studies]; SEAR South East Asia Region [n = 3 studies]; WPR, Western Pacific Region [n = 11 studies]; MRS, Multiregional Studies [n = 3 studies]) pooled using inverse variance weights. Heterogeneity (I^2) = AFR (.%), AMR (99.55%), EMR (97.23%), EUR (99.58%), SEAR (.%), WPR (99.48%), MRS (.%) Each black-colored solid square represents the effect size of each characteristic, while the ends of the adjoining horizontal lines represent lower (left) and upper (right) confidence intervals. The blue-colored hollow diamond at the bottom denotes the overall estimated effect size and the 95% CI. All statistics were based on a two-sided t-test. ES, Effect Size.

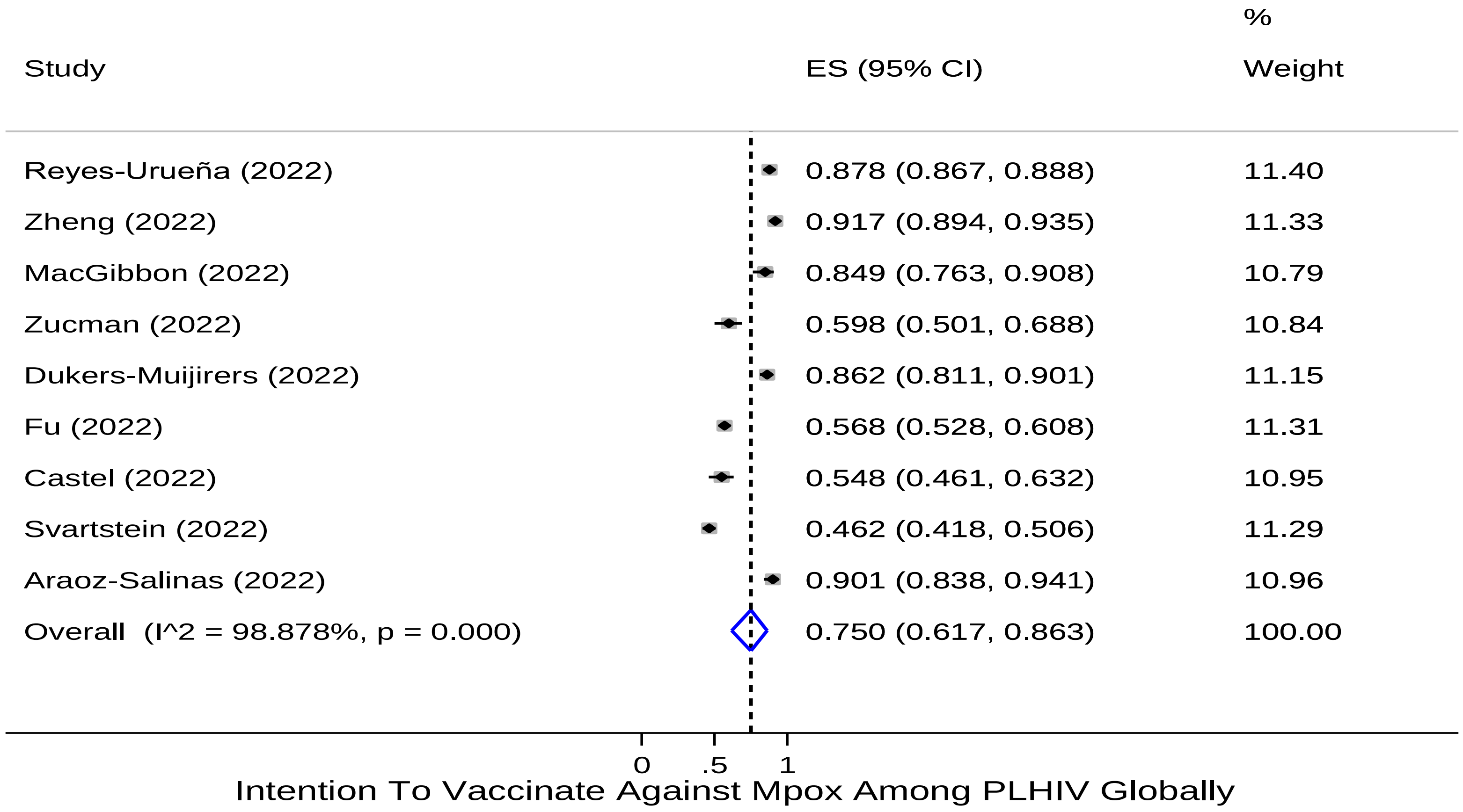

Supplementary Figure 39: Forest plots of the results of random-effects model meta-analysis of the prevalence (%) of mpox vaccine intention among PLHIV globally (n = 9 studies) pooled using inverse variance weights. Heterogeneity (I^2) = 98.88%. Each black-colored solid square represents the effect size of each characteristic, while the ends of the adjoining horizontal lines represent lower (left) and upper (right) confidence intervals. The blue-colored hollow diamond at the bottom denotes the overall estimated effect size and the 95% CI. All statistics were based on a two-sided t-test. ES, Effect Size.

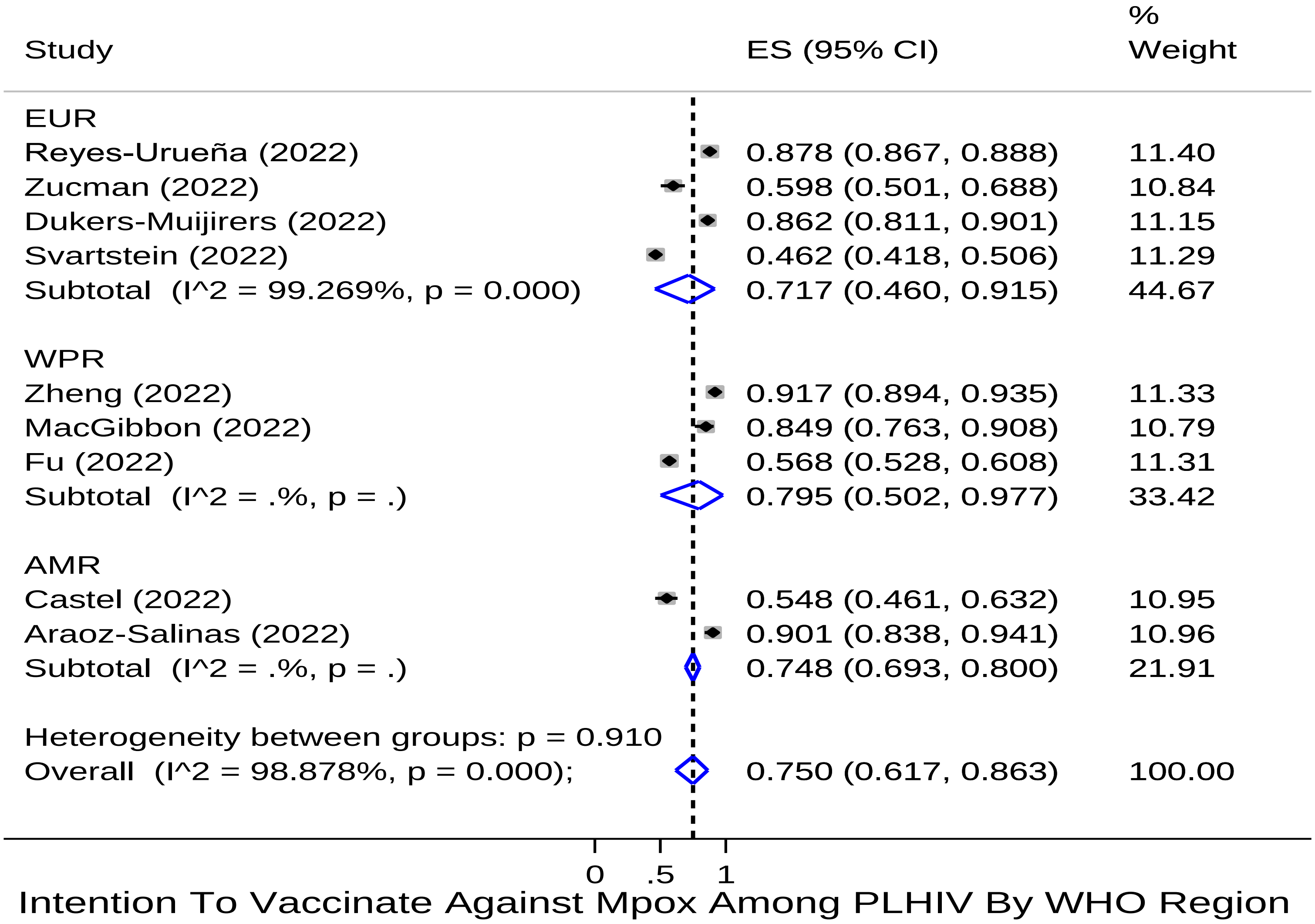

Supplementary Figure 40: Forest plots of the results of random-effects model meta-analysis of the prevalence (%) of mpox vaccine acceptance according to WHO region (AMR, Region of the Americas [n = 3 studies]; EMR, EUR, European Region [n = 5 studies]; WPR, Western Pacific Region [n = 3 studies]) pooled using inverse variance weights. Heterogeneity (I^2) = AMR (.%), EUR (99.26%), WPR (.%). Each black-colored solid square represents the effect size of each characteristic, while the ends of the adjoining horizontal lines represent lower (left) and upper (right) confidence intervals. The blue-colored hollow diamond at the bottom denotes the overall estimated effect size and the 95% CI. All statistics were based on a two-sided t-test. ES, Effect Size.

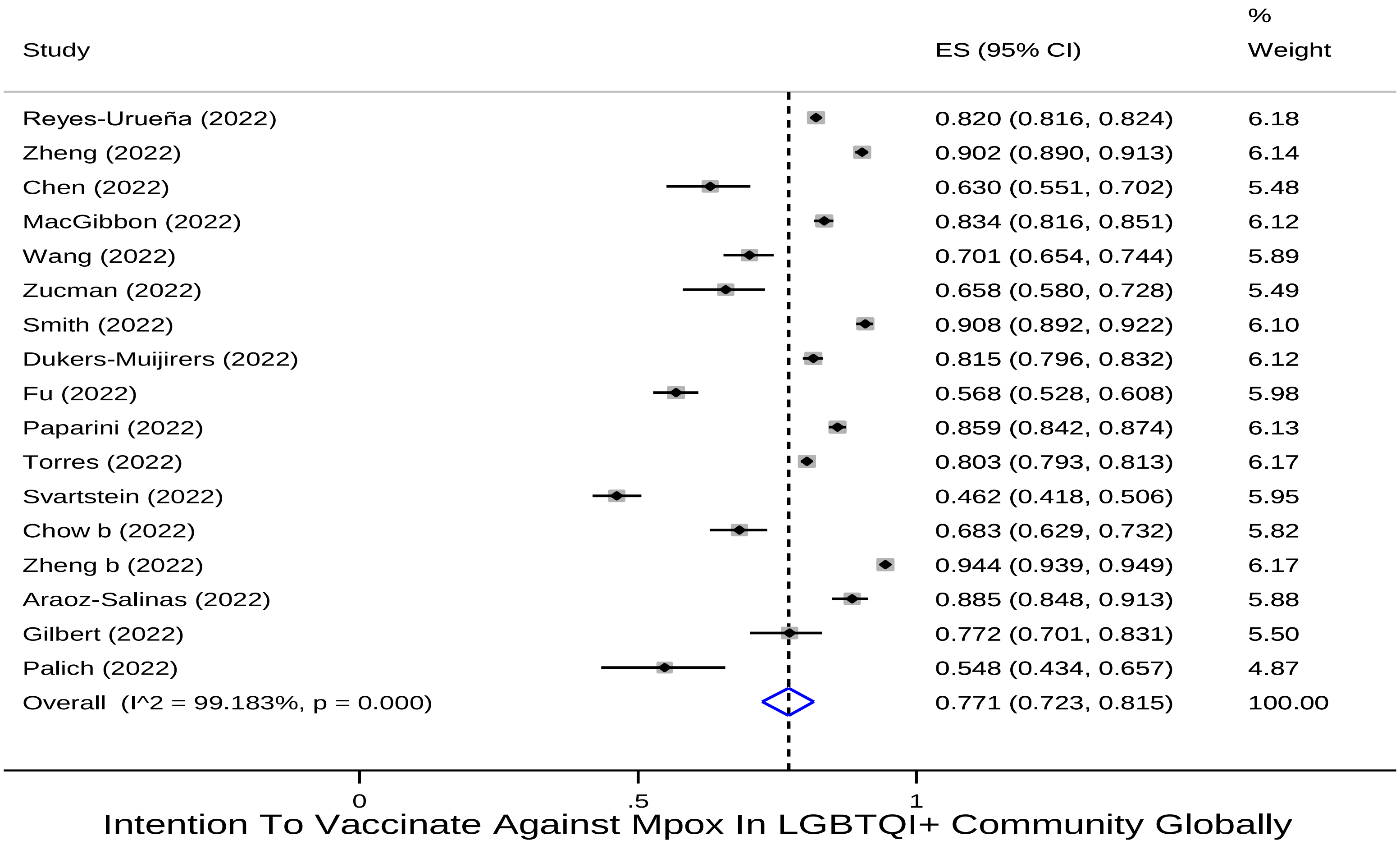

Supplementary Figure 41: Forest plots of the results of random-effects model meta-analysis of the prevalence (%) of intention to vaccinate against mpox among the LGBTQI+ community globally (n = 17 studies) pooled using inverse variance weights. Heterogeneity (I^2) = 99.18%. Each black-colored solid square represents the effect size of each characteristic, while the ends of the adjoining horizontal lines represent lower (left) and upper (right) confidence intervals. The blue-colored hollow diamond at the bottom denotes the overall estimated effect size and the 95% CI. All statistics were based on a two-sided t-test. ES, Effect Size.

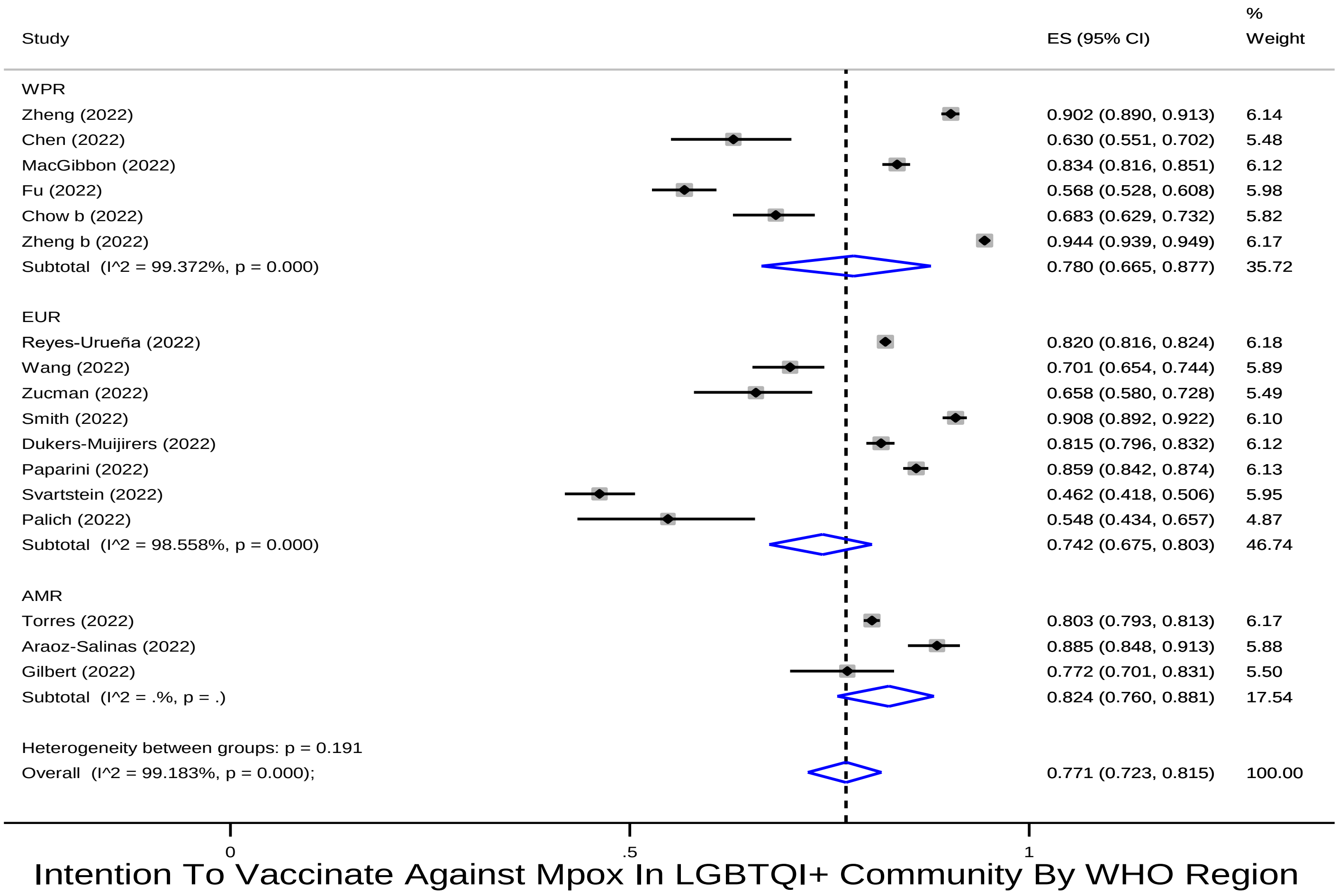

Supplementary Figure 42: Forest plots of the results of random-effects model meta-analysis of the prevalence (%) of mpox vaccine uptake according to WHO region (AMR, Region of the Americas [n = 3 studies]; EMR, EUR, European Region [n = 8 studies]; WPR, Western Pacific Region [n = 6 studies]) pooled using inverse variance weights. Heterogeneity (I^2) = AMR (.%), EUR (98.58%), WPR (99.37%). Each black-colored solid square represents the effect size of each characteristic, while the ends of the adjoining horizontal lines represent lower (left) and upper (right) confidence intervals. The blue-colored hollow diamond at the bottom denotes the overall estimated effect size and the 95% CI. All statistics were based on a two-sided t-test. ES, Effect Size.

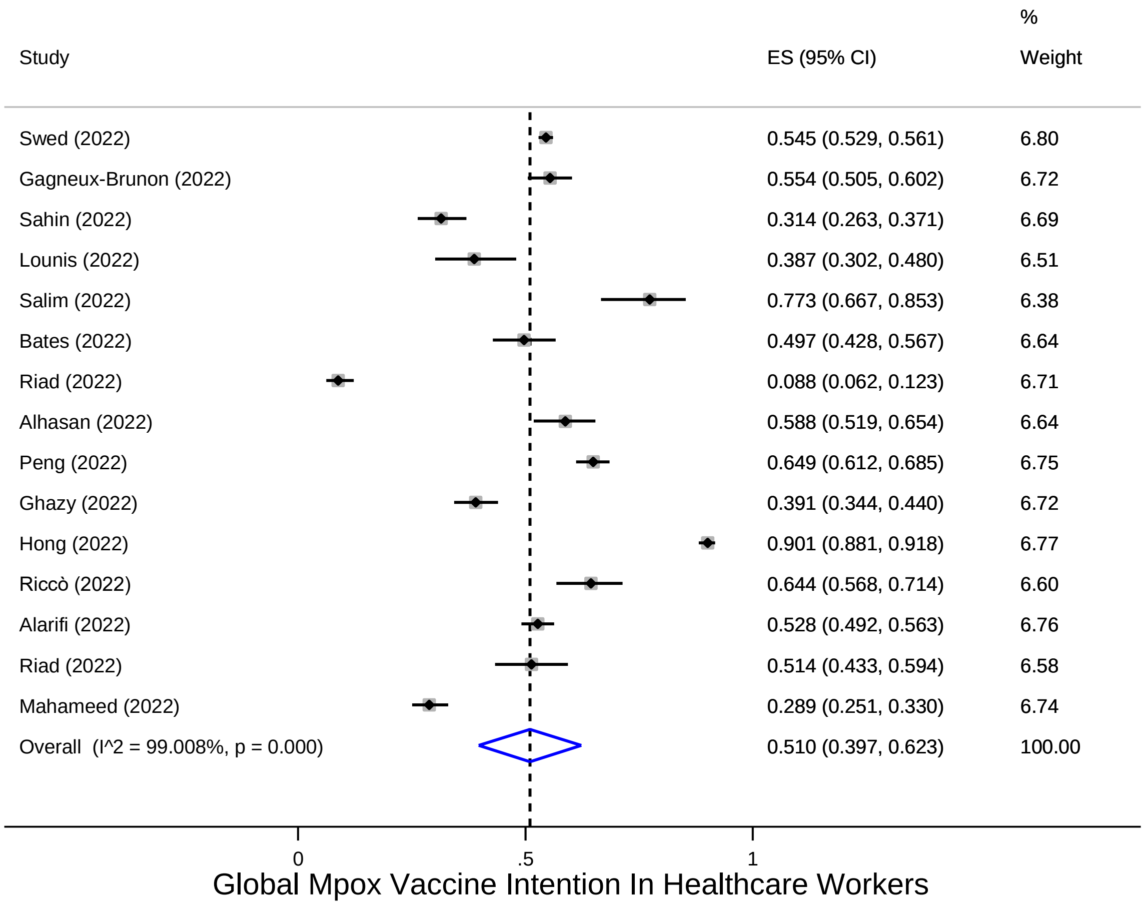

Supplementary Figure 43: Forest plots of the results of random-effects model meta-analysis of the prevalence (%) of the intention to vaccinate against mpox among healthcare workers globally (n = 15 studies) pooled using inverse variance weights. Heterogeneity (I^2) = 99.01%. Each black-colored solid square represents the effect size of each characteristic, while the ends of the adjoining horizontal lines represent lower (left) and upper (right) confidence intervals. The blue-colored hollow diamond at the bottom denotes the overall estimated effect size and the 95% CI. All statistics were based on a two-sided t-test. ES, Effect Size.

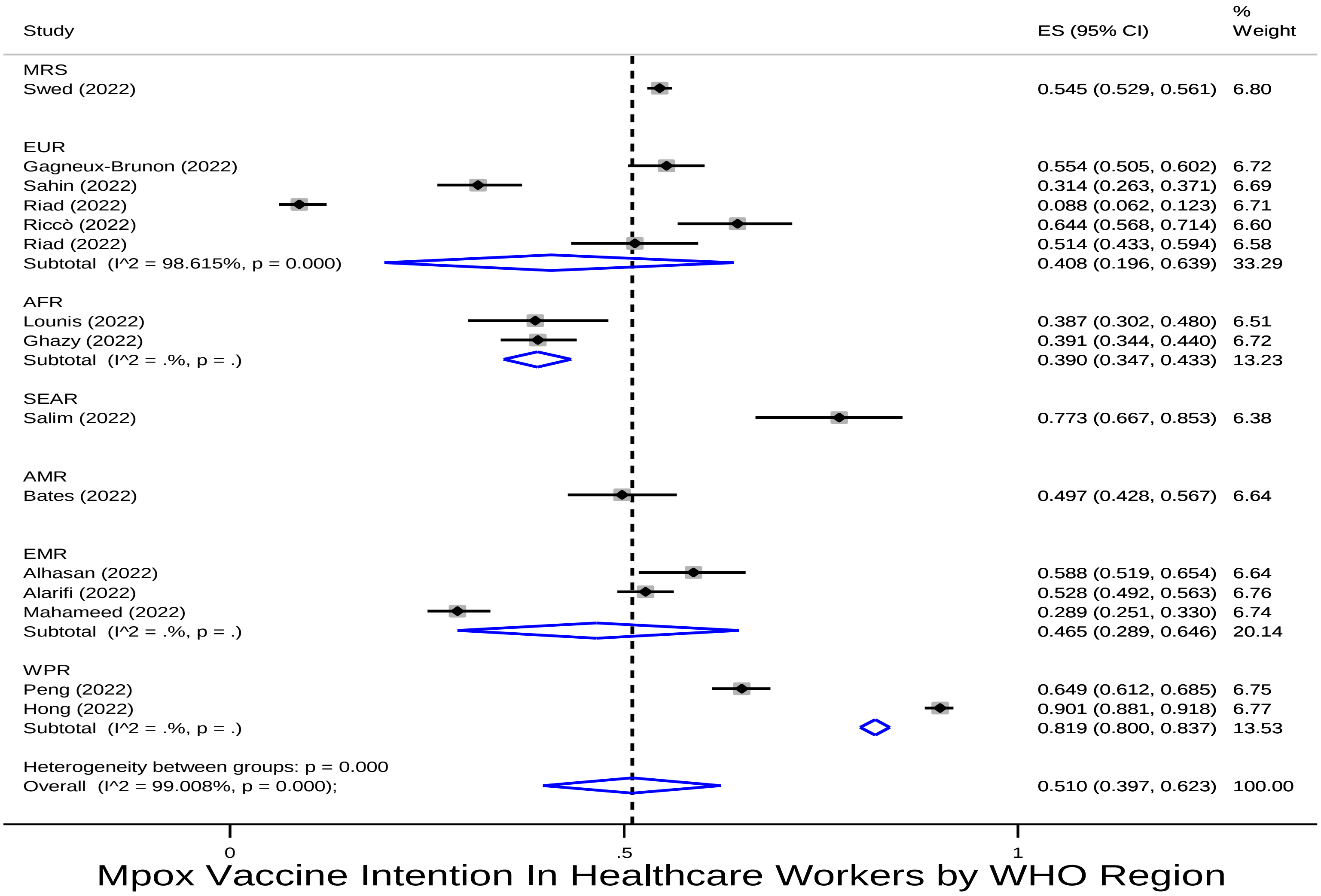

Supplementary Figure 44: Forest plots of the results of random-effects model meta-analysis of the prevalence (%) intention to vaccinate against mpox among healthcare workers according to WHO region (AFR, Africa [n = 2 studies]; AMR, Region of the Americas [n = 1 studies]; EMR, Eastern Mediterranean Region [n = 3 studies]; EUR, European Region [n = 5 studies]; SEAR South East Asia Region [n = 1 studies]; WPR, Western Pacific Region [n = 2 studies]; MRS, Multiregional Studies [n = 1 studies]) pooled using inverse variance weights. Heterogeneity (I^2) = AFR (.%), AMR (.%), EMR (.%), EUR (98.62%), SEAR (.%), WPR (.%), and MRS (.%). Each black-colored solid square represents the effect size of each characteristic, while the ends of the adjoining horizontal lines represent lower (left) and upper (right) confidence intervals. The blue-colored hollow diamond at the bottom denotes the overall estimated effect size and the 95% CI. All statistics were based on a two-sided t-test. ES, Effect Size.

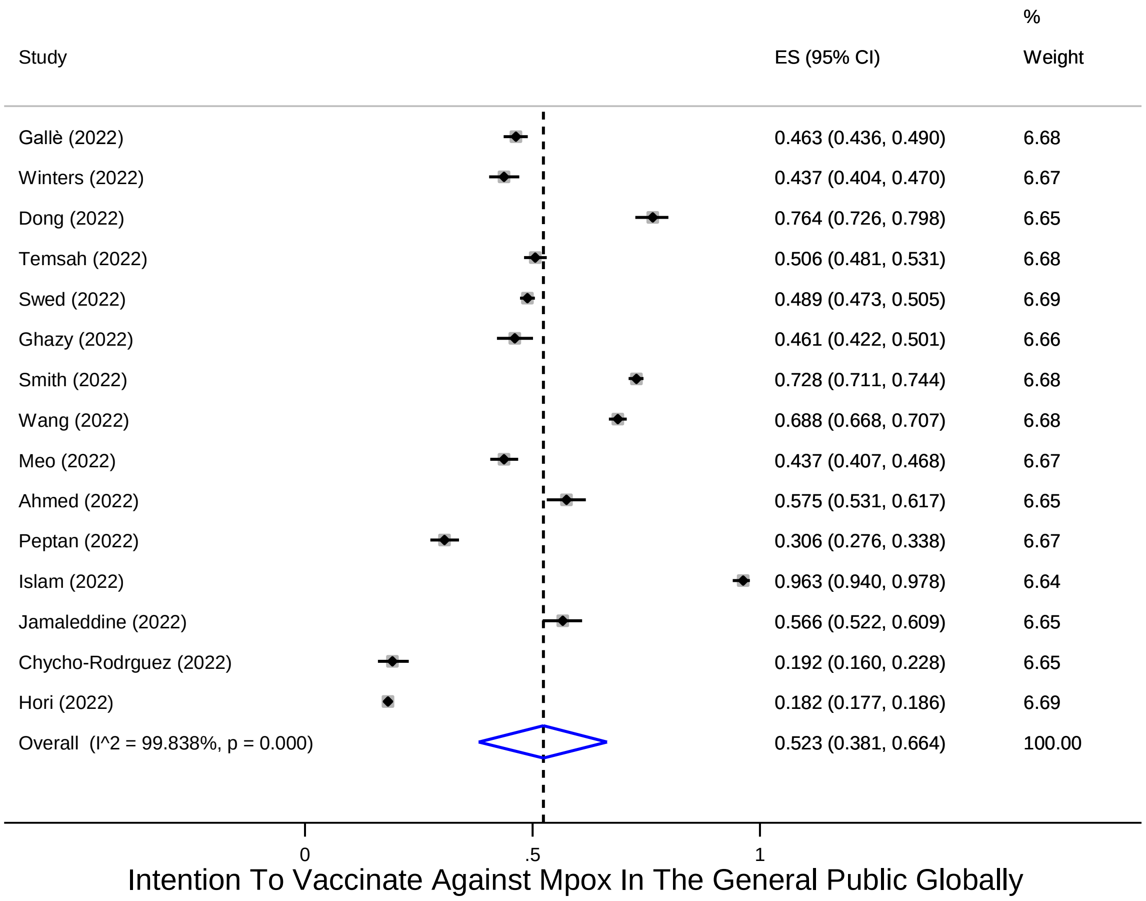

Supplementary Figure 45: Forest plots of the results of random-effects model meta-analysis of the prevalence (%) of intention to vaccinate against mpox among the general public globally (n = 15 studies) pooled using inverse variance weights. Heterogeneity (I^2) = 99.84%. Each black-colored solid square represents the effect size of each characteristic, while the ends of the adjoining horizontal lines represent lower (left) and upper (right) confidence intervals. The blue-colored hollow diamond at the bottom denotes the overall estimated effect size and the 95% CI. All statistics were based on a two-sided t-test. ES, Effect Size.

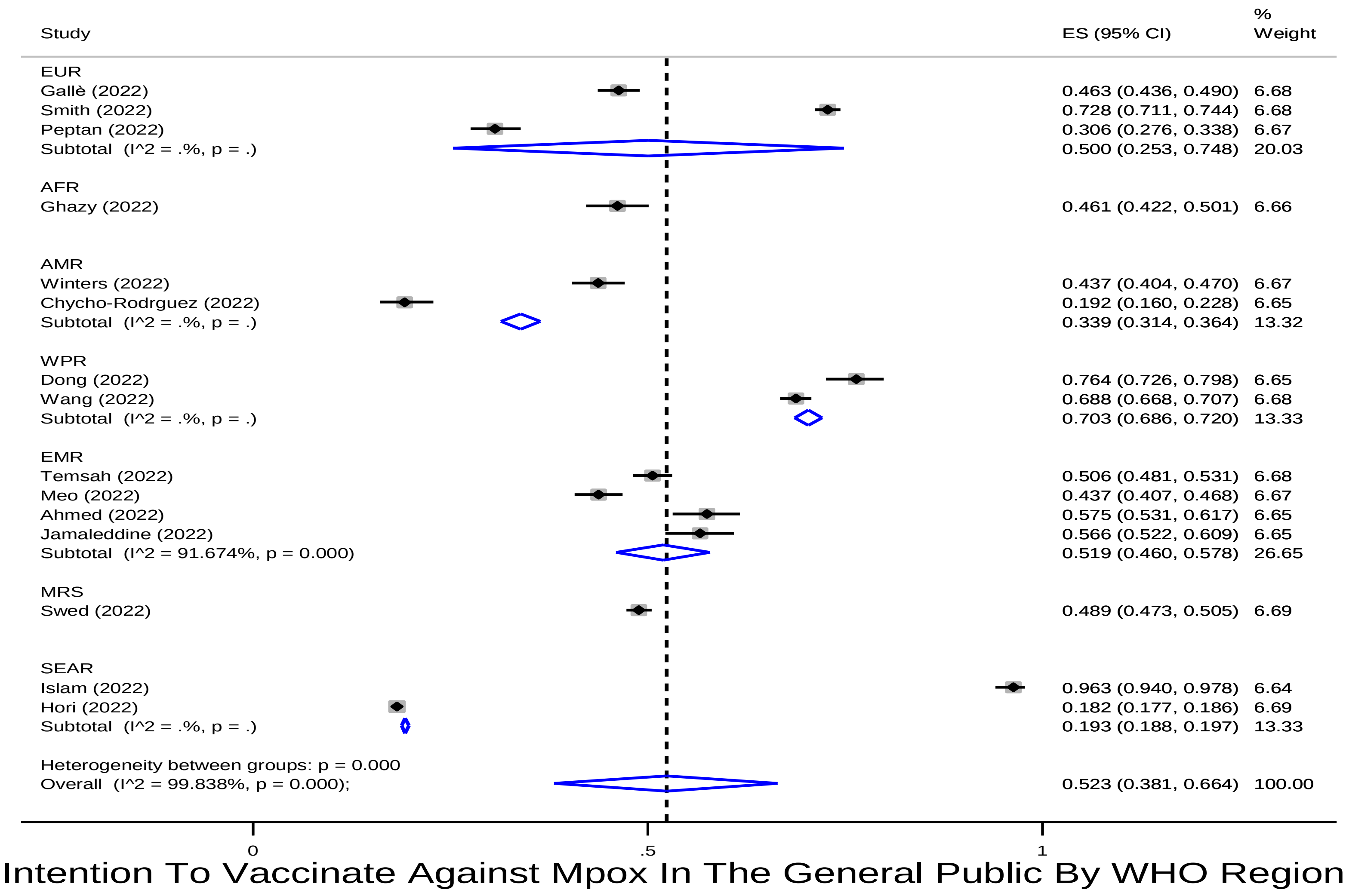

Supplementary Figure 46: Forest plots of the results of random-effects model meta-analysis of the prevalence (%) intention to vaccinate against mpox among the general public according to WHO region (AFR, Africa [n = 1 study]; AMR, Region of the Americas [n = 2 studies]; EMR, Eastern Mediterranean Region [n = 4 studies]; EUR, European Region [n = 3 studies]; SEAR South East Asia Region [n = 2 studies]; WPR, Western Pacific Region [n = 2 studies]; MRS, Multiregional Studies [n = 1 studies]) pooled using inverse variance weights. Heterogeneity (I^2) = AFR (.%), AMR (.%), EMR (91.67.%), EUR (98.62%), SEAR (.%), WPR (.%), and MRS (.%). Each black-colored solid square represents the effect size of each characteristic, while the ends of the adjoining horizontal lines represent lower (left) and upper (right) confidence intervals. The blue-colored hollow diamond at the bottom denotes the overall estimated effect size and the 95% CI. All statistics were based on a two-sided t-test. ES, Effect Size.

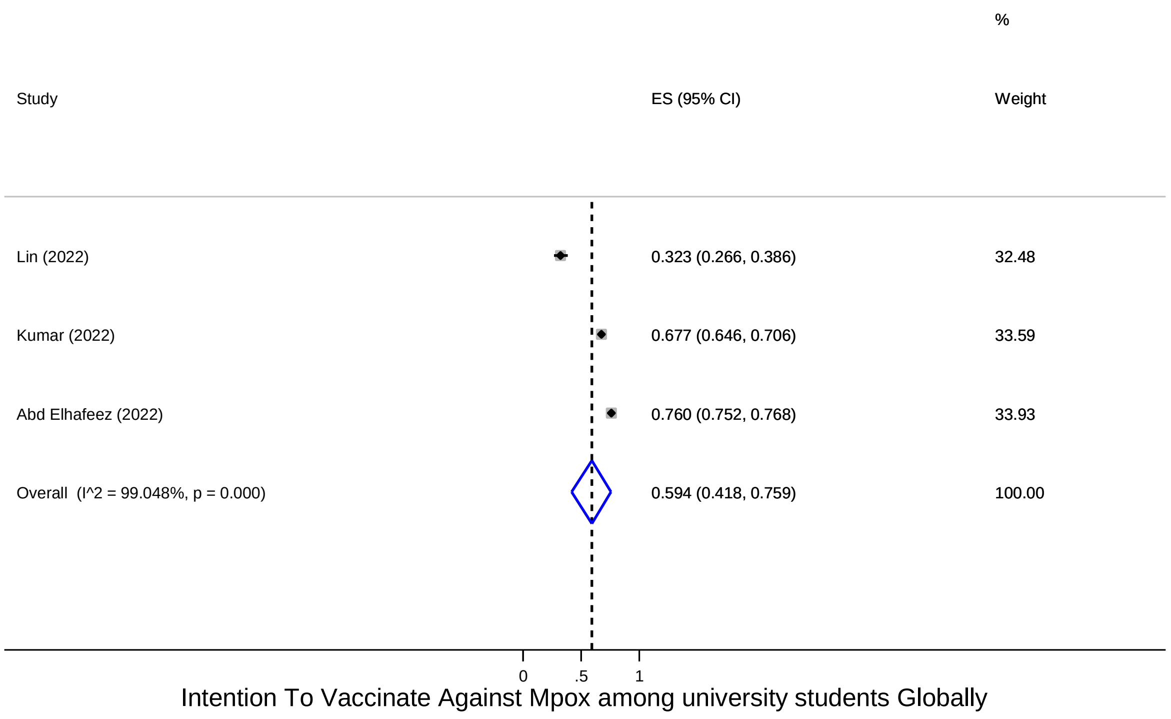

Supplementary Figure 47: Forest plots of the results of random-effects model meta-analysis of the prevalence (%) intention to vaccinate against mpox among university students globally (n = 3 studies) pooled using inverse variance weights. Heterogeneity (I^2) = 99.05%. Each black-colored solid square represents the effect size of each characteristic, while the ends of the adjoining horizontal lines represent lower (left) and upper (right) confidence intervals. The blue-colored hollow diamond at the bottom denotes the overall estimated effect size and the 95% CI. All statistics were based on a two-sided t-test. ES, Effect Size.

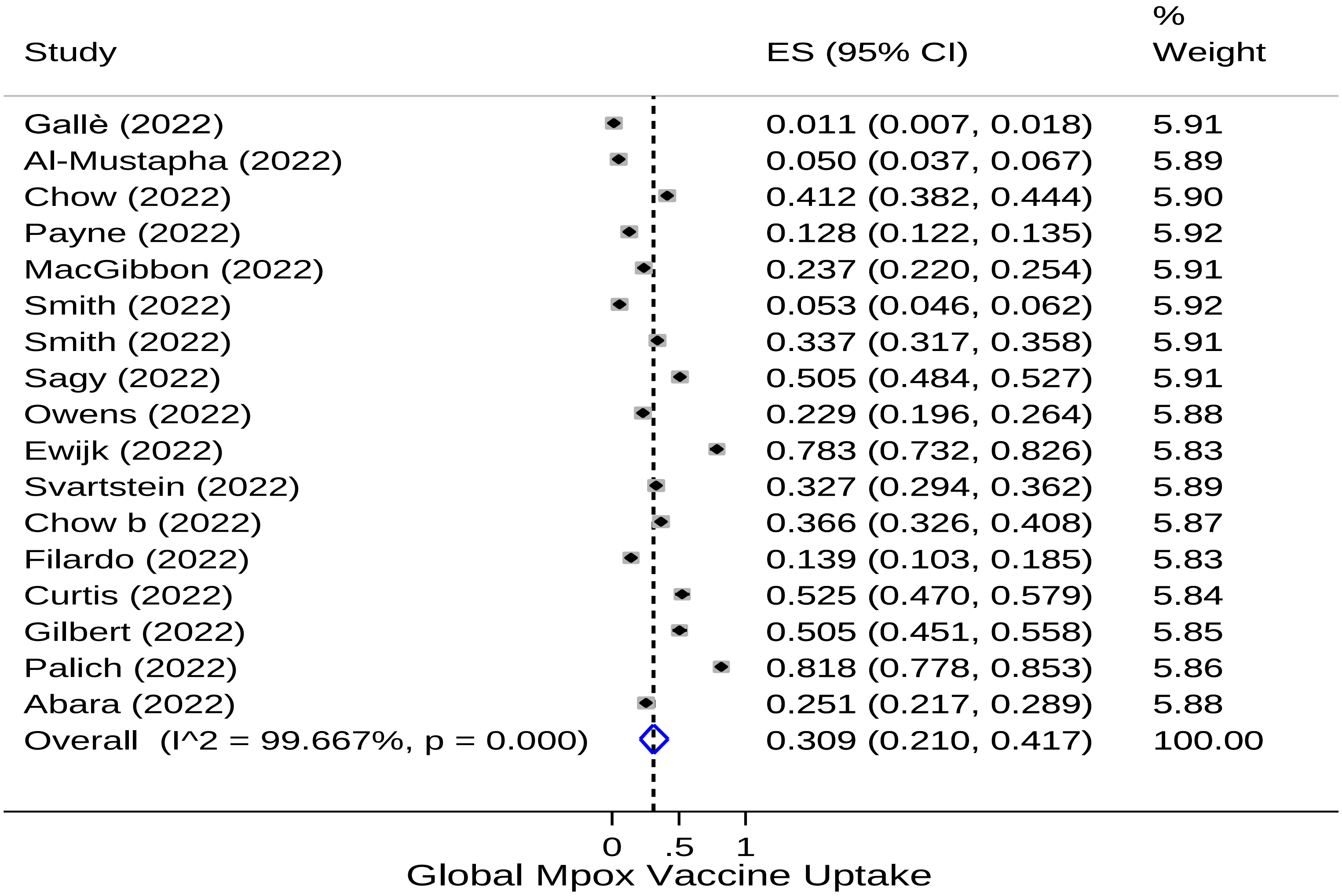

Supplementary Figure 48: Forest plots of the results of random-effects model meta-analysis of the prevalence (%) of uptake of the mpox vaccine globally (n = 17 studies) pooled using inverse variance weights. Heterogeneity (I^2) = 99.67%. Each black-colored solid square represents the effect size of each characteristic, while the ends of the adjoining horizontal lines represent lower (left) and upper (right) confidence intervals. The blue-colored hollow diamond at the bottom denotes the overall estimated effect size and the 95% CI. All statistics were based on a two-sided t-test. ES, Effect Size.

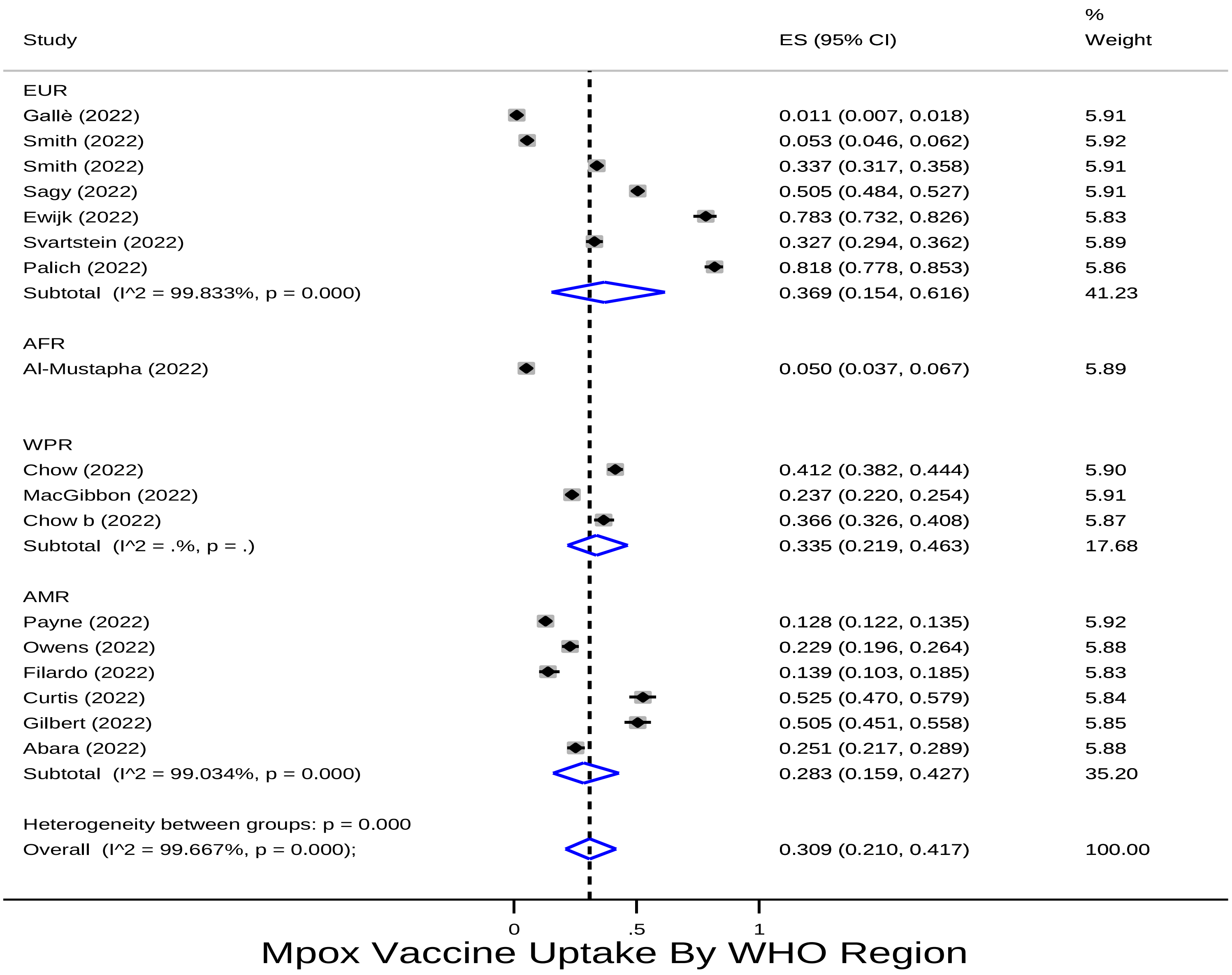

Supplementary Figure 49: Forest plots of the results of random-effects model meta-analysis of the prevalence (%) of mpox vaccine uptake according to WHO region (AFR, Africa [n = 1 studies); AMR, Region of the Americas [n = 6 studies]; EUR, European Region [n = 7 studies]; WPR, Western Pacific Region [n = 3 studies]; MRS, Multiregional Studies [n = 2 studies]) pooled using inverse variance weights. Heterogeneity (I^2) = AFR (.%), AMR (99.03%), EUR (99.83%), WPR (.%). Each black-colored solid square represents the effect size of each characteristic, while the ends of the adjoining horizontal lines represent lower (left) and upper (right) confidence intervals. The blue-colored hollow diamond at the bottom denotes the overall estimated effect size and the 95% CI. All statistics were based on a two-sided t-test. ES, Effect Size.

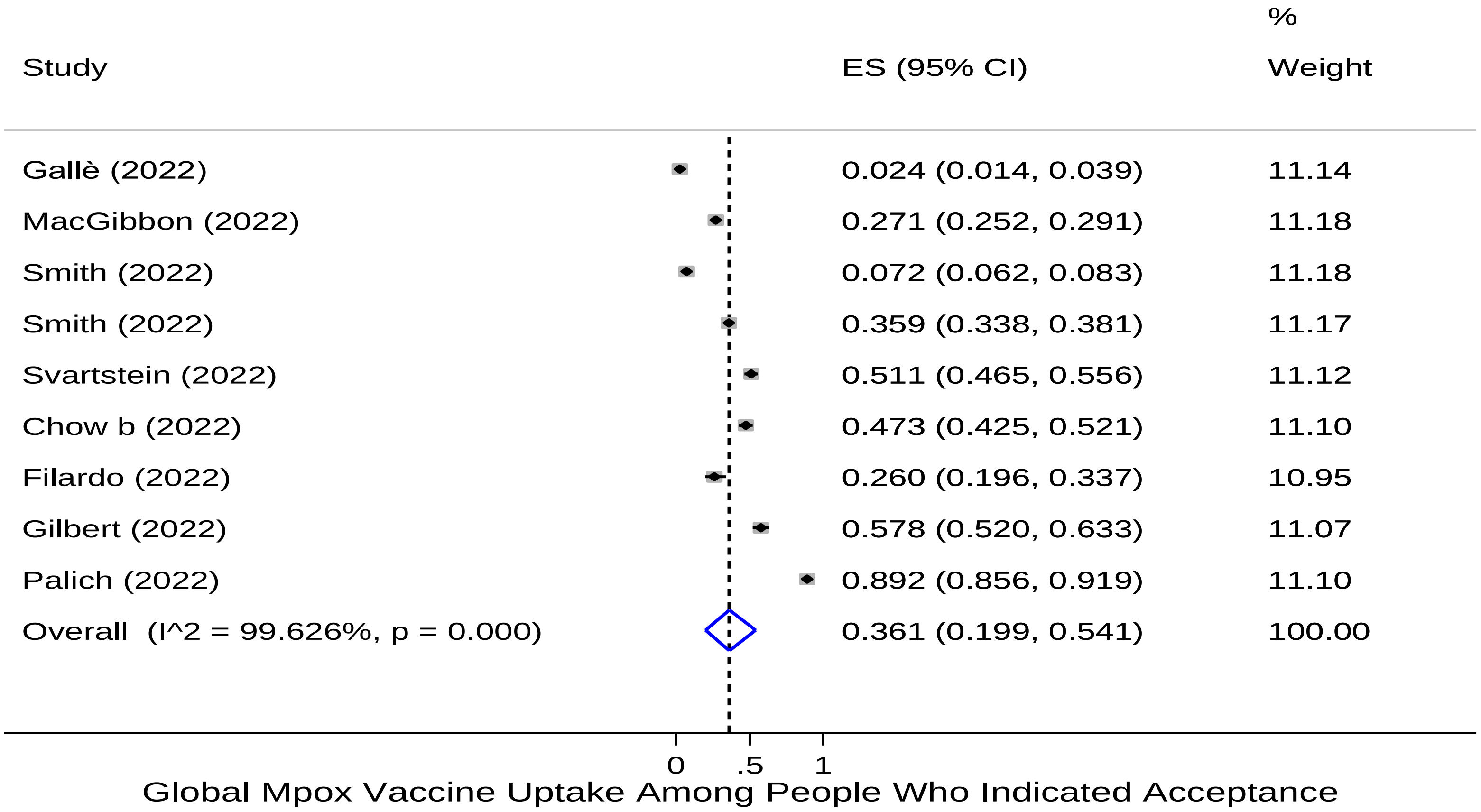

Supplementary Figure 50: Forest plots of the results of random-effects model meta-analysis of the prevalence (%) of mpox vaccine uptake among the accepting group globally (n = 9 studies) pooled using inverse variance weights. Heterogeneity (I^2) = 99.63%. Each black-colored solid square represents the effect size of each characteristic, while the ends of the adjoining horizontal lines represent lower (left) and upper (right) confidence intervals. The blue-colored hollow diamond at the bottom denotes the overall estimated effect size and the 95% CI. All statistics were based on a two-sided t-test. ES, Effect Size

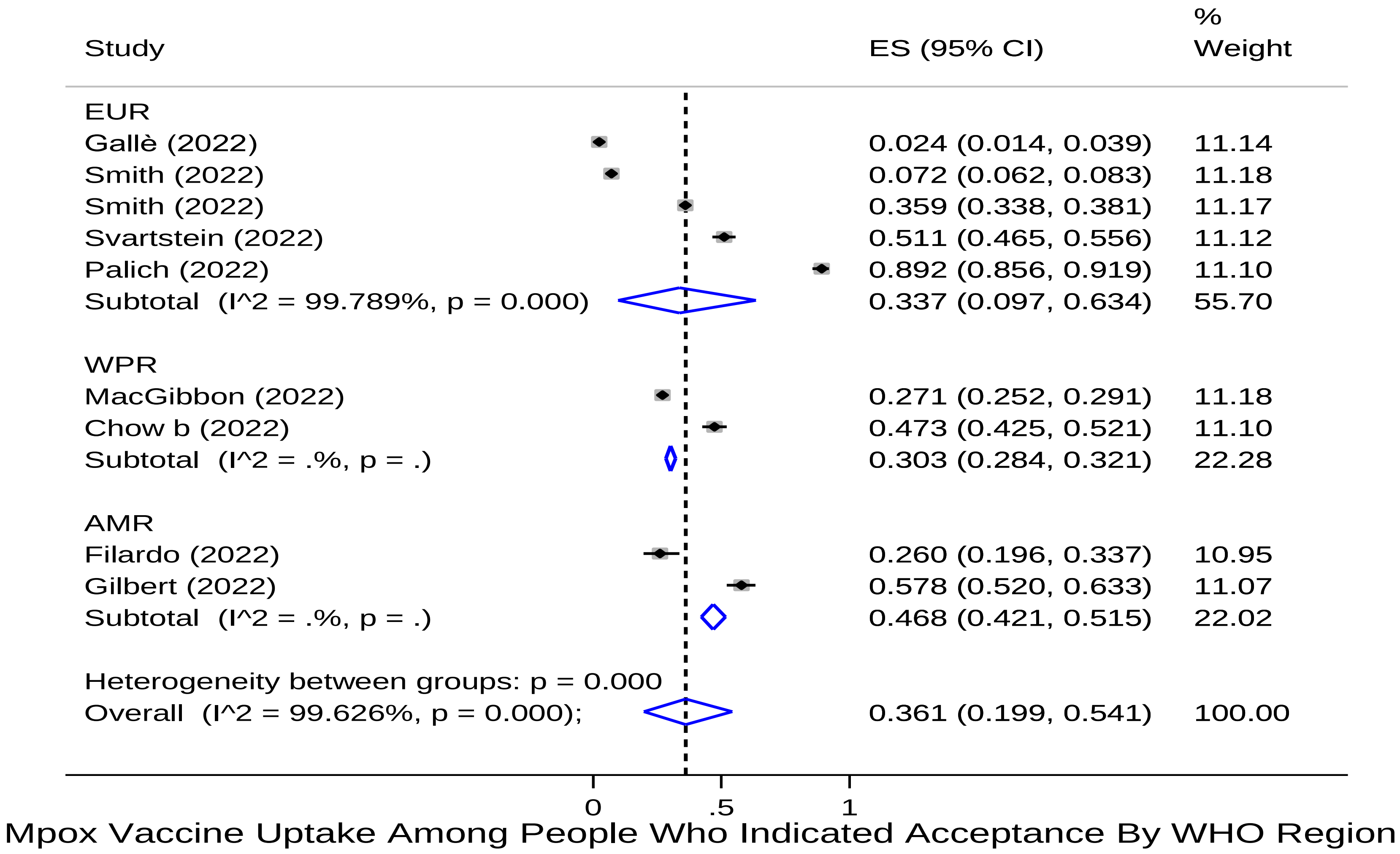

Supplementary Figure 51: Forest plots of the results of random-effects model meta-analysis of the prevalence (%) of mpox vaccine uptake among accepting group according to WHO region (AMR, Region of the Americas [n = 2 studies]; EUR, European Region [n = 5 studies]; WPR, Western Pacific Region [n = 2 studies]; MRS, Multiregional Studies [n = 2 studies]) pooled using inverse variance weights. Heterogeneity (I^2) = AMR (.%), EUR (99.79%), WPR (.%). Each black-colored solid square represents the effect size of each characteristic, while the ends of the adjoining horizontal lines represent lower (left) and upper (right) confidence intervals. The blue-colored hollow diamond at the bottom denotes the overall estimated effect size and the 95% CI. All statistics were based on a two-sided t-test. ES, Effect Size.

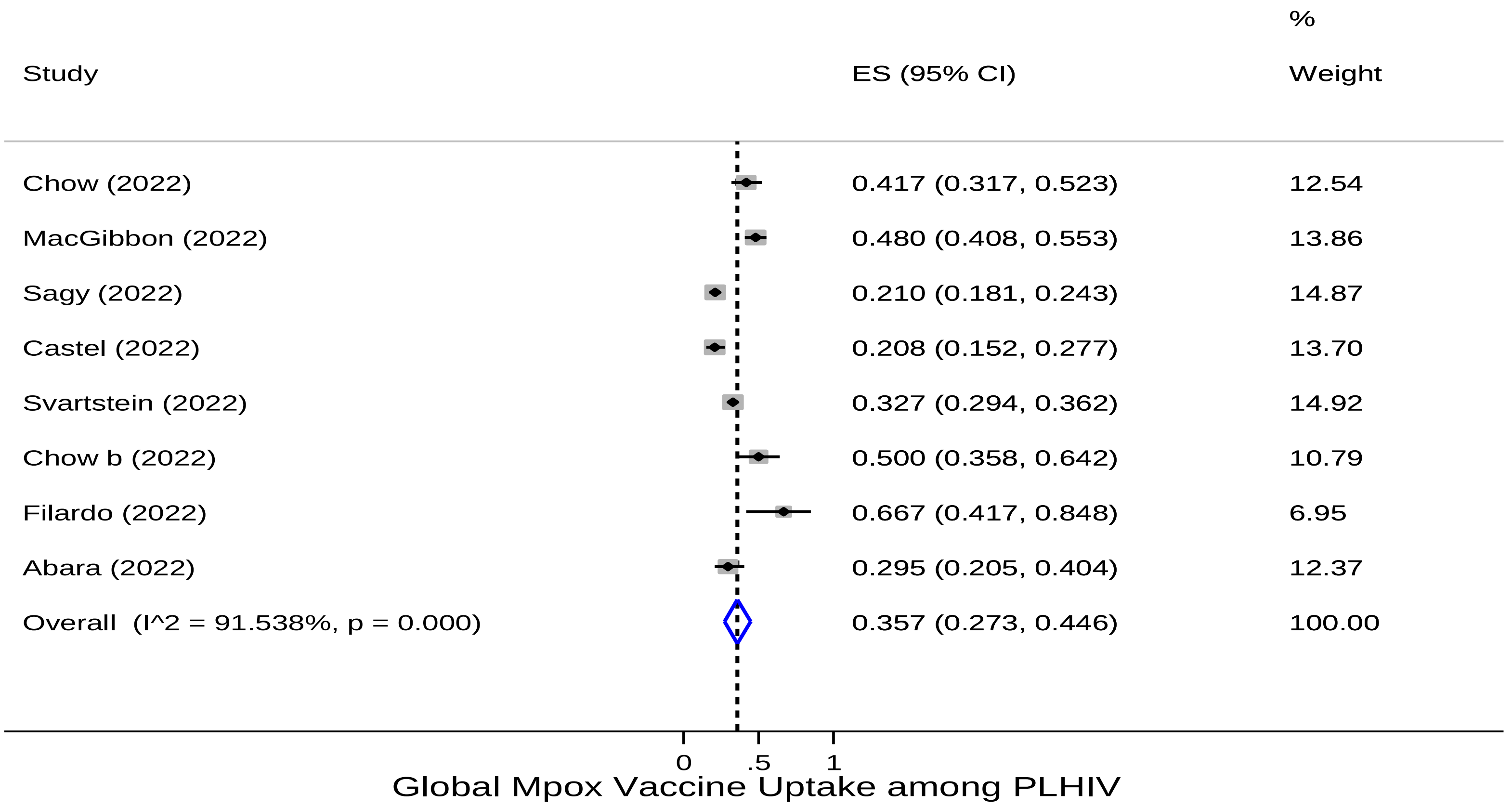

Supplementary Figure 52: Forest plots of the results of random-effects model meta-analysis of the prevalence (%) of mpox vaccine uptake among PLHIV globally (n = 8 studies) pooled using inverse variance weights. Heterogeneity (I^2) = 91.54%. Each black-colored solid square represents the effect size of each characteristic, while the ends of the adjoining horizontal lines represent lower (left) and upper (right) confidence intervals. The blue-colored hollow diamond at the bottom denotes the overall estimated effect size and the 95% CI. All statistics were based on a two-sided t-test. ES, Effect Size.

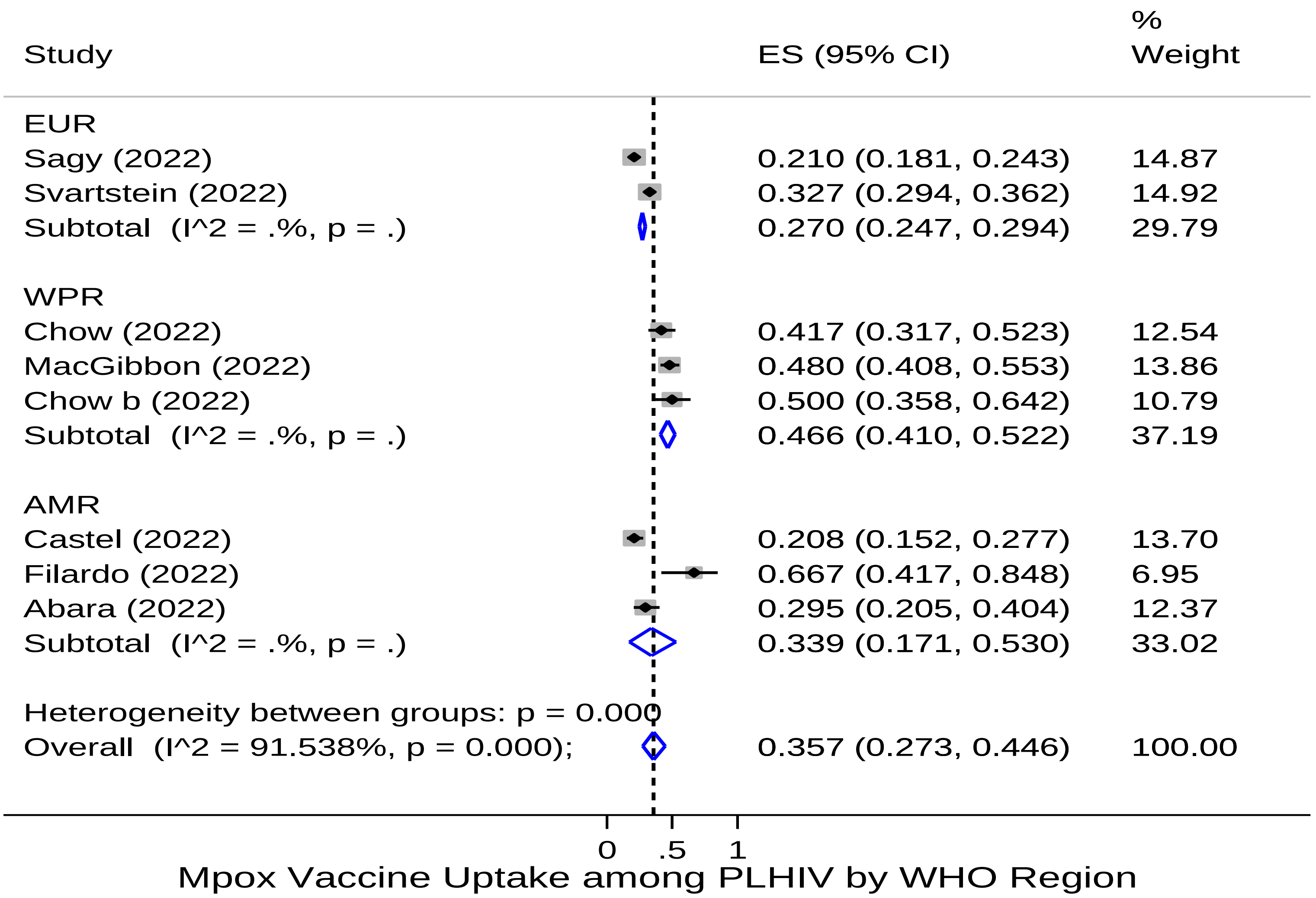

Supplementary Figure 53: Forest plots of the results of random-effects model meta-analysis of the prevalence (%) of mpox vaccine uptake according to WHO region (AMR, Region of the Americas [n = 3 studies]; EMR, EUR, European Region [n = 3 studies]; WPR, Western Pacific Region [n = 3 studies]) pooled using inverse variance weights. Heterogeneity (I^2) = AMR (.%), EUR (.%), WPR (.%). Each black-colored solid square represents the effect size of each characteristic, while the ends of the adjoining horizontal lines represent lower (left) and upper (right) confidence intervals. The blue-colored hollow diamond at the bottom denotes the overall estimated effect size and the 95% CI. All statistics were based on a two-sided t-test. ES, Effect Size.

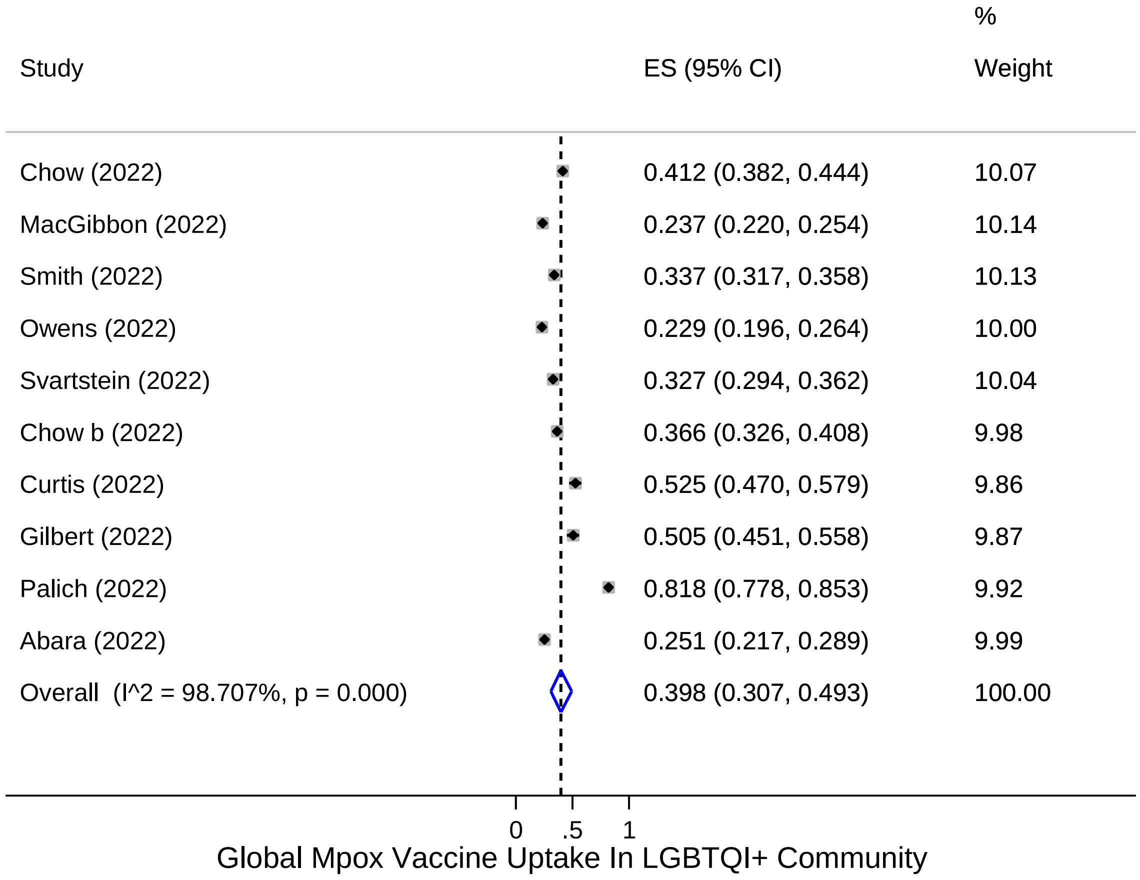

Supplementary Figure 54: Forest plots of the results of random-effects model meta-analysis of the prevalence (%) of mpox vaccine uptake among the LGBTQI+ community globally (n = 10 studies) pooled using inverse variance weights. Heterogeneity (I^2) = 98.71%. Each black-colored solid square represents the effect size of each characteristic, while the ends of the adjoining horizontal lines represent lower (left) and upper (right) confidence intervals. The blue-colored hollow diamond at the bottom denotes the overall estimated effect size and the 95% CI. All statistics were based on a two-sided t-test. ES, Effect Size.

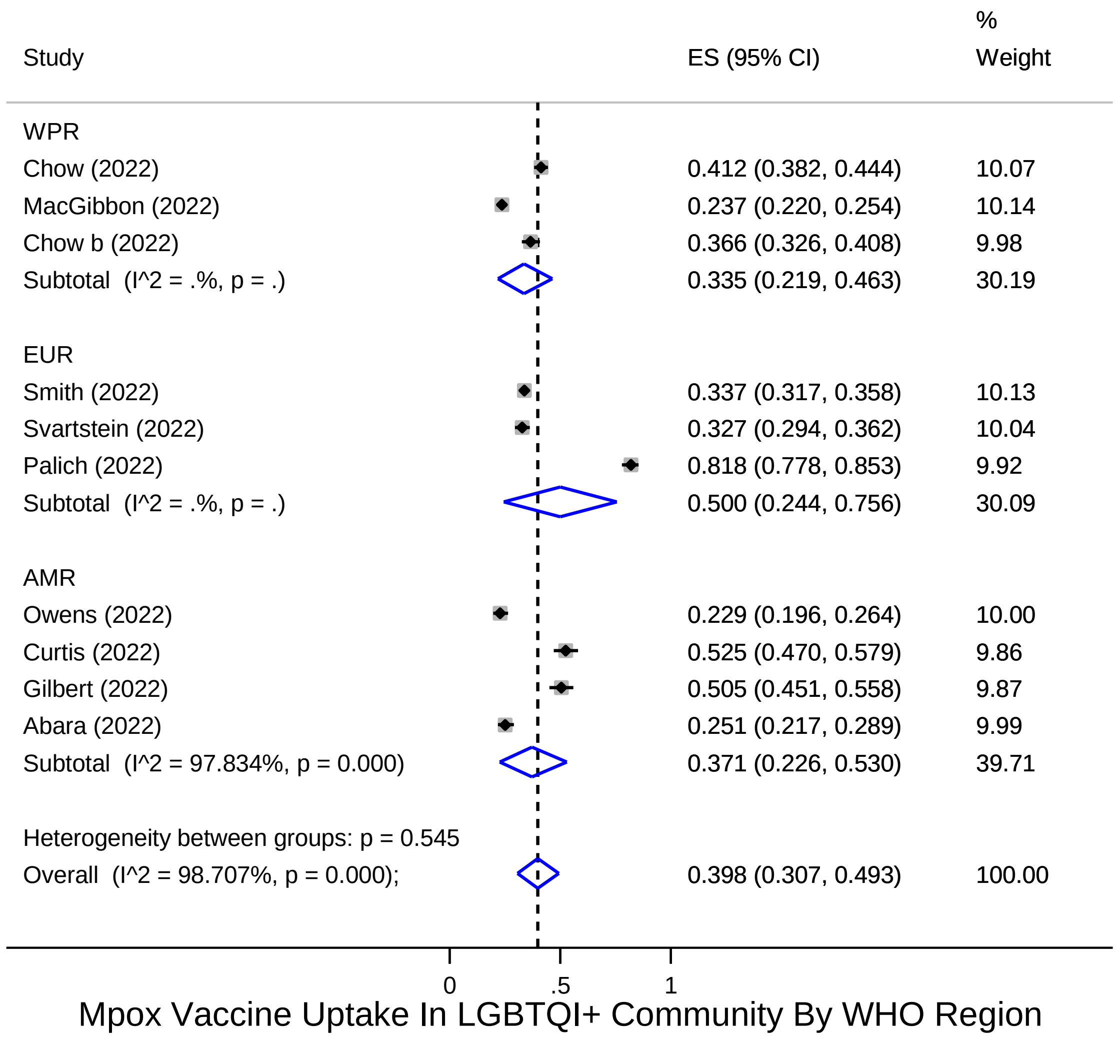

Supplementary Figure 55: Forest plots of the results of random-effects model meta-analysis of the prevalence (%) of mpox vaccine uptake according to WHO region (AMR, Region of the Americas [n = 4 studies]; EMR, EUR, European Region [n = 3 studies]; WPR, Western Pacific Region [n = 3 studies]) pooled using inverse variance weights. Heterogeneity (I^2) = AMR (97.83%), EUR (.%), WPR (.%). Each black-colored solid square represents the effect size of each characteristic, while the ends of the adjoining horizontal lines represent lower (left) and upper (right) confidence intervals. The blue-colored hollow diamond at the bottom denotes the overall estimated effect size and the 95% CI. All statistics were based on a two-sided t-test. ES, Effect Size.

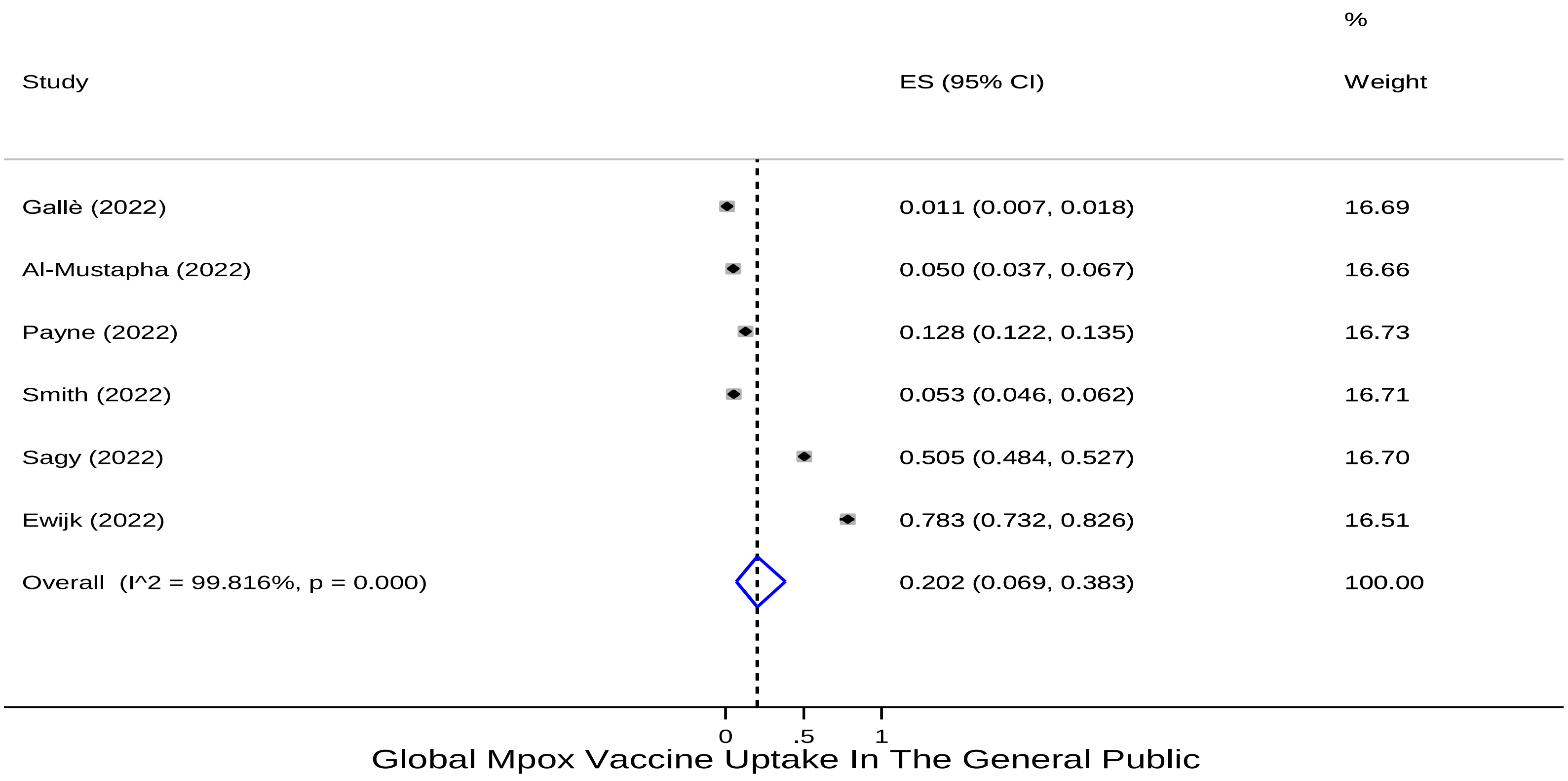

Supplementary Figure 56: Forest plots of the results of random-effects model meta-analysis of the prevalence (%) of intention to mpox vaccine uptake among the general public globally (n = 6 studies) pooled using inverse variance weights. Heterogeneity (I^2) = 99.82%. Each black-colored solid square represents the effect size of each characteristic, while the ends of the adjoining horizontal lines represent lower (left) and upper (right) confidence intervals. The blue-colored hollow diamond at the bottom denotes the overall estimated effect size and the 95% CI. All statistics were based on a two-sided t-test. ES, Effect Size.

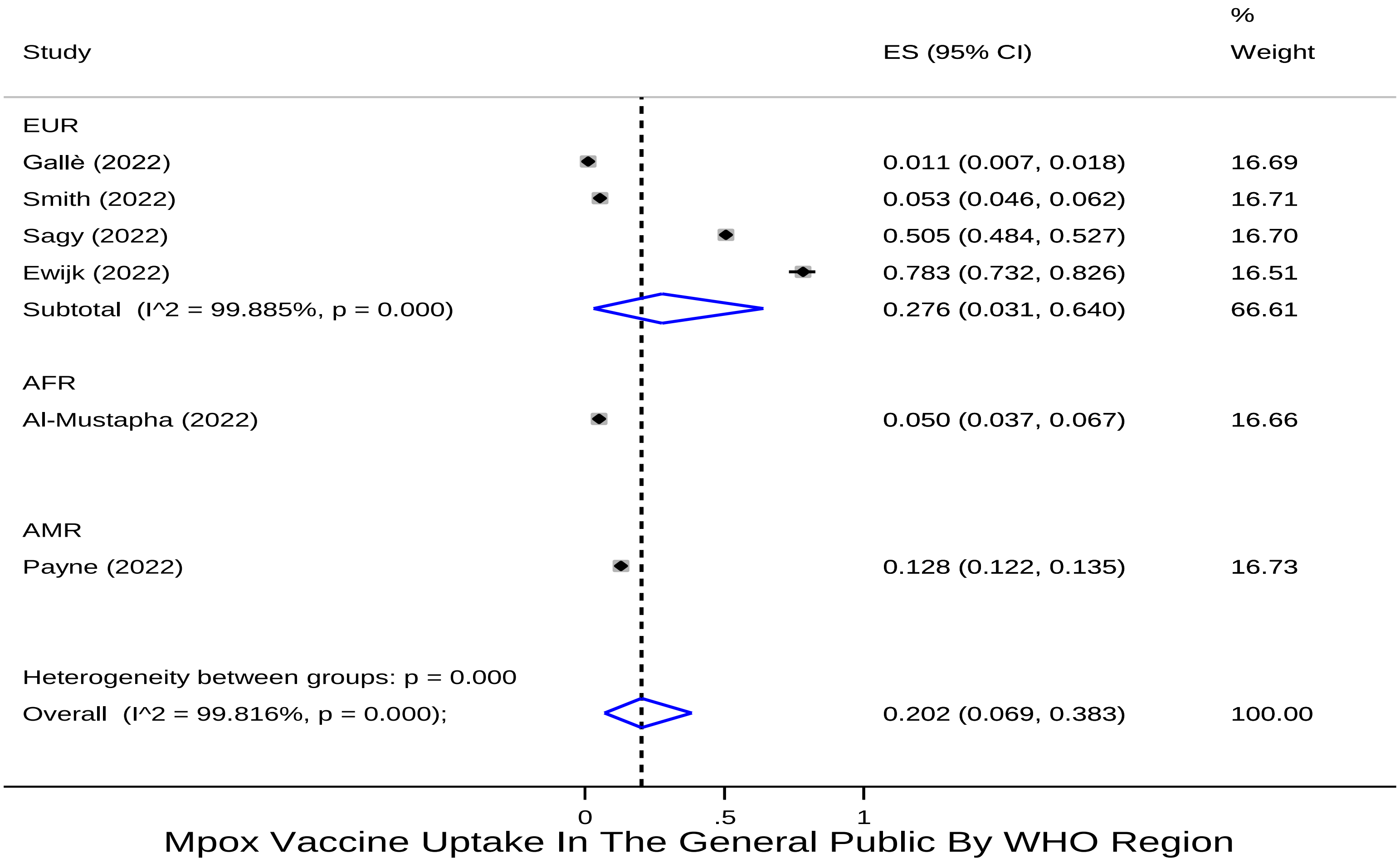

Supplementary Figure 57: Forest plots of the results of random-effects model meta-analysis of the prevalence (%) mpox vaccine uptake among the general public according to WHO region (AFR, Africa [n = 1 study]; AMR, Region of the Americas [n = 2 studies]; EMR, Eastern Mediterranean Region [n = 4 studies]; EUR, European Region [n = 3 studies]; SEAR South East Asia Region [n = 2 studies]; WPR, Western Pacific Region [n = 2 studies]; MRS, Multiregional Studies [n = 1 studies]) pooled using inverse variance weights. Heterogeneity (I^2) = AFR (.%), AMR (.%), EMR (91.67.%), EUR (98.62%), SEAR (.%), WPR (.%), and MRS (.%). Each black-colored solid square represents the effect size of each characteristic, while the ends of the adjoining horizontal lines represent lower (left) and upper (right) confidence intervals. The blue-colored hollow diamond at the bottom denotes the overall estimated effect size and the 95% CI. All statistics were based on a two-sided t-test. ES, Effect Size.

Results of sensitivity analysis for all outcomes (Supplementary Figures 58 – 72)

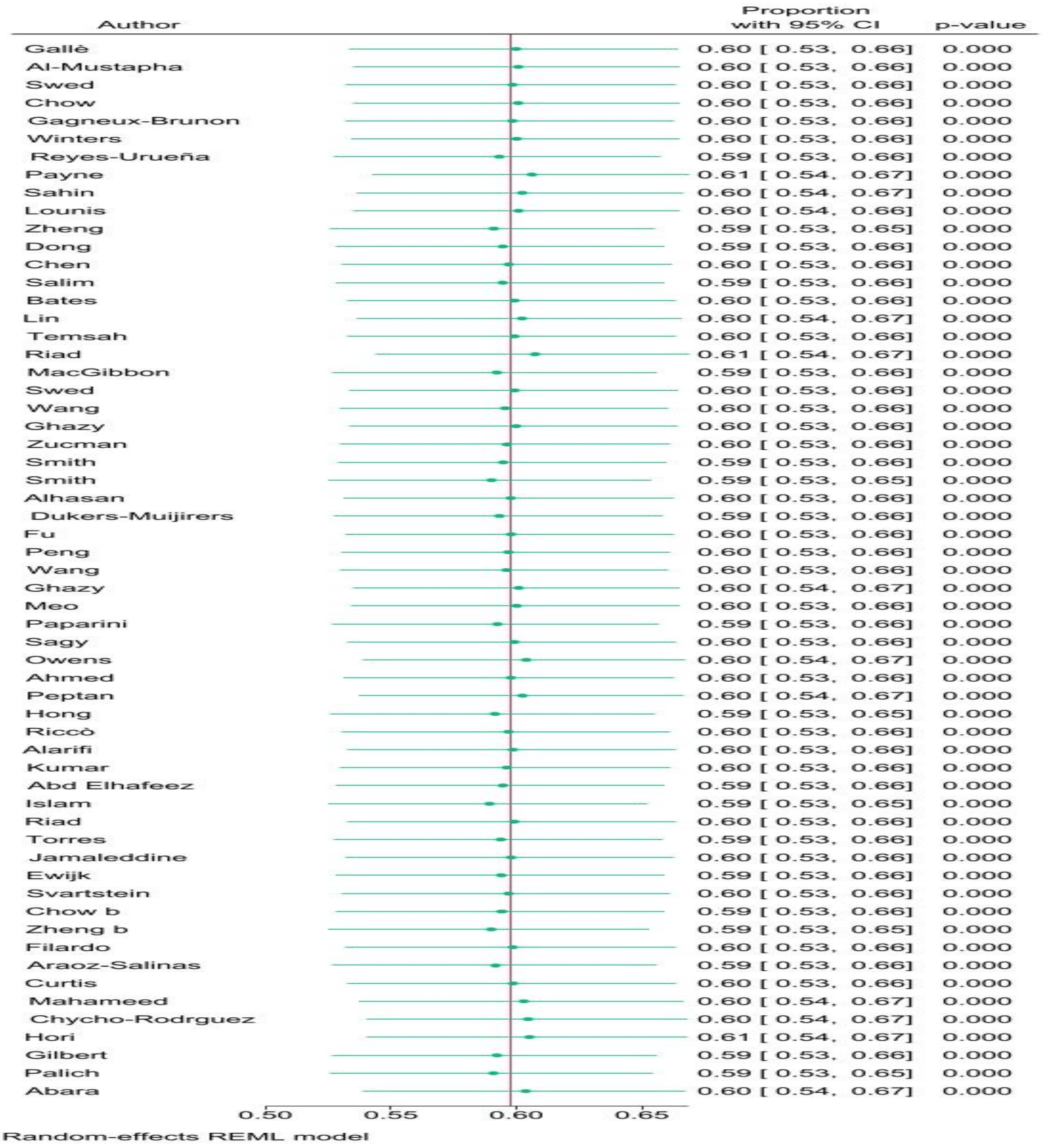

Supplementary Figure 58: Forest plots of the results of leave-one-out sensitivity analysis of the overall prevalence (%) of mpox vaccine acceptance globally (n = 59 studies) pooled using inverse variance weights. Results were based on random-effects meta-analysis. Each green-colored solid square represents the effect size of each characteristic, while the ends of the adjoining horizontal lines represent lower (left) and upper (right) confidence intervals. All statistics were based on a two-sided t-test. REML, Restricted Maximum Likelihood.

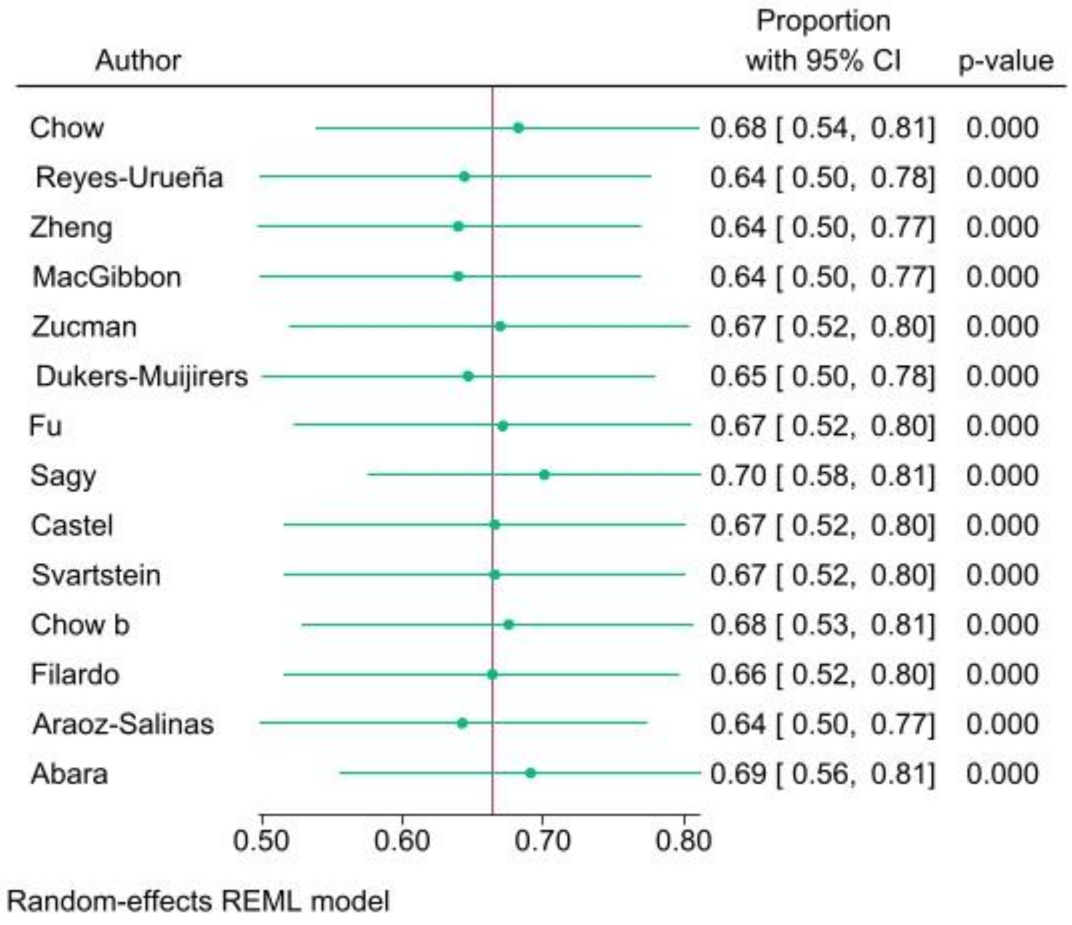

Supplementary Figure 59: Forest plots of the results of leave-one-out sensitivity analysis of the prevalence (%) of mpox vaccine acceptance among PLHIV (n = 14 studies) pooled using inverse variance weights. Results were based on random-effects meta-analysis. Each green-colored solid square represents the effect size of each study characteristic, while the ends of the adjoining horizontal lines represent lower (left) and upper (right) confidence intervals. All statistics were based on a two-sided t-test. REML, Restricted Maximum Likelihood.

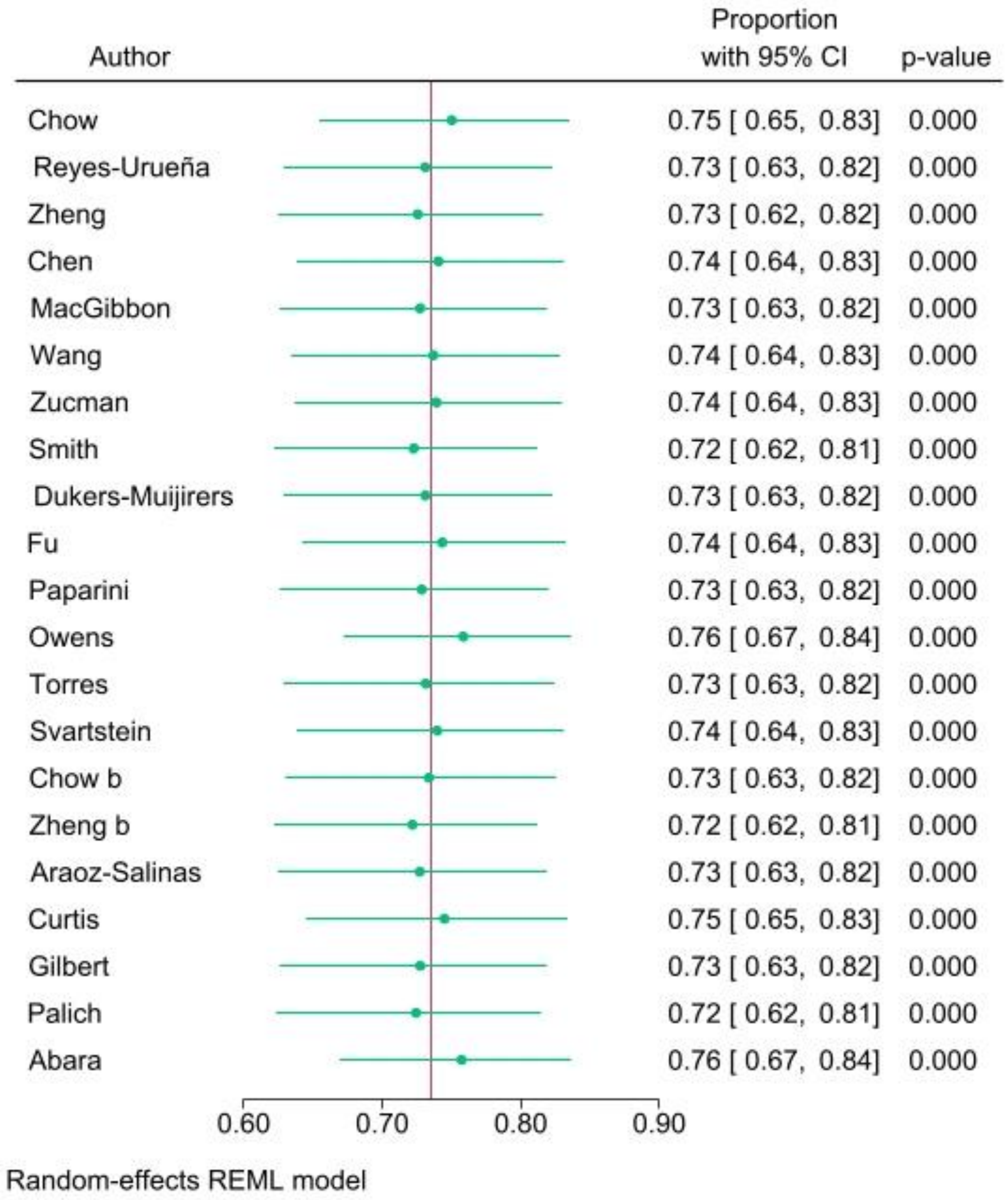

Supplementary Figure 60: Forest plots of the results of leave-one-out sensitivity analysis of the prevalence (%) of mpox vaccine acceptance among the LGBTQI+ community (n = 21 studies) pooled using inverse variance weights. Results were based on random-effects meta-analysis. Each green-colored solid square represents the effect size of each study characteristic, while the ends of the adjoining horizontal lines represent lower (left) and upper (right) confidence intervals. All statistics were based on a two-sided t-test. REML, Restricted Maximum Likelihood.

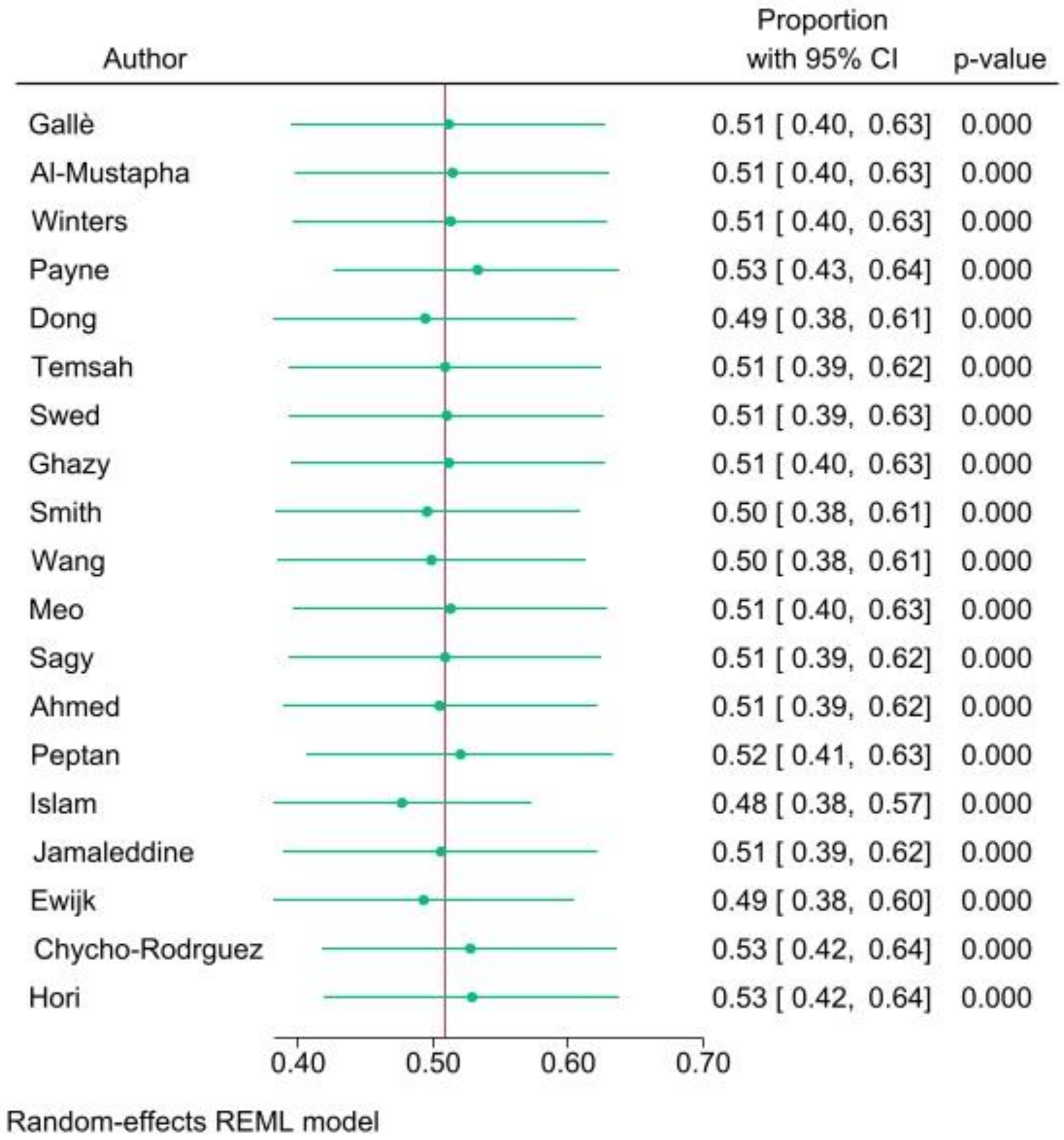

Supplementary Figure 61: Forest plots of the results of leave-one-out sensitivity analysis of the prevalence (%) of mpox vaccine acceptance among the general public (n = 19 studies) pooled using inverse variance weights. Results were based on random-effects meta-analysis. Each green-colored solid square represents the effect size of each study characteristic, while the ends of the adjoining horizontal lines represent lower (left) and upper (right) confidence intervals. All statistics were based on a two-sided t-test. REML, Restricted Maximum Likelihood.

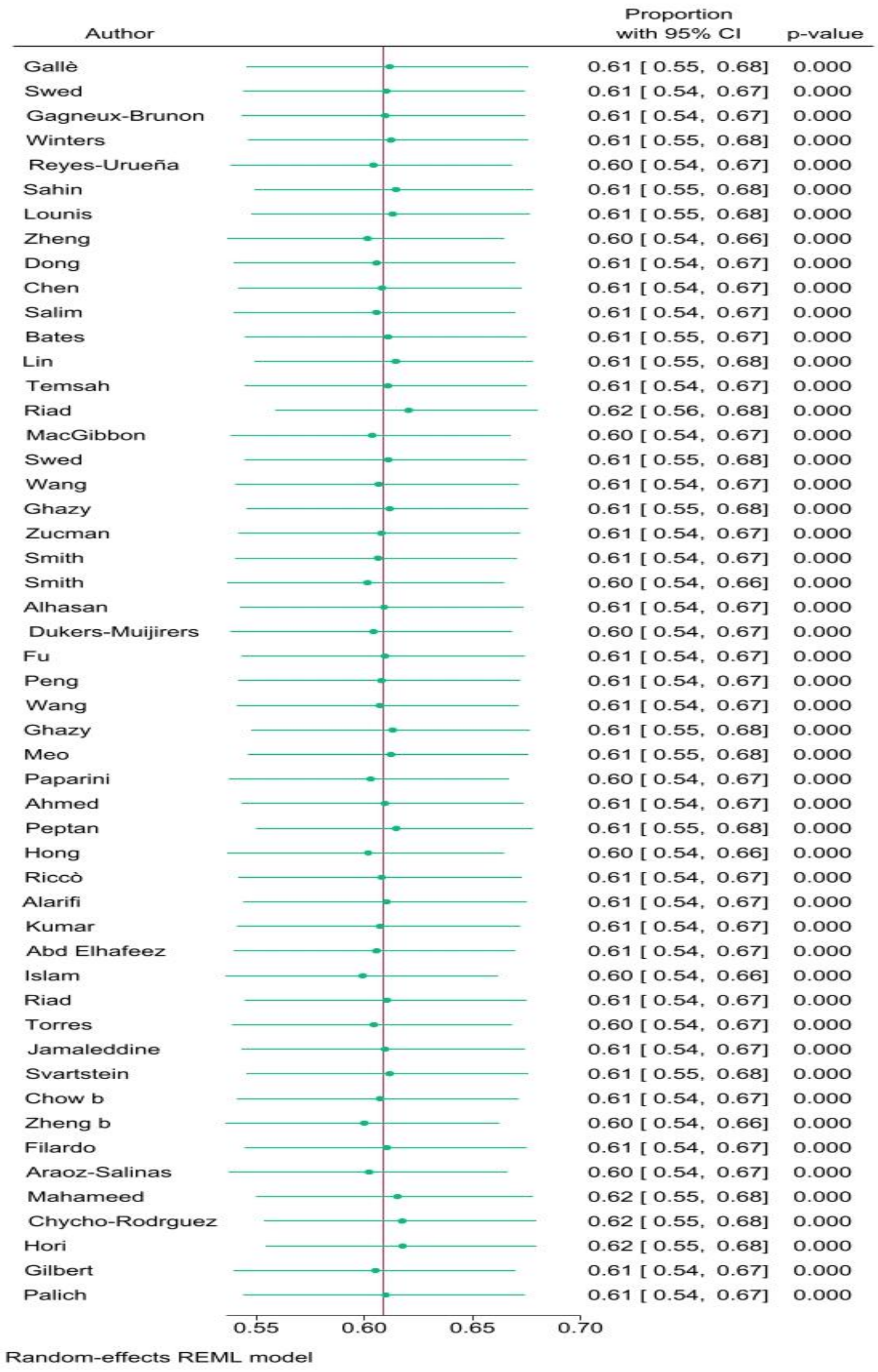

Supplementary Figure 62: Forest plots of the results of leave-one-out sensitivity analysis of the overall prevalence (%) of mpox vaccine intention globally (n = 51 studies) pooled using inverse variance weights. Results were based on random-effects meta-analysis. Each green-colored solid square represents the effect size of each study characteristic, while the ends of the adjoining horizontal

lines represent lower (left) and upper (right) confidence intervals. All statistics were based on a two-sided t-test. REML, Restricted Maximum Likelihood.

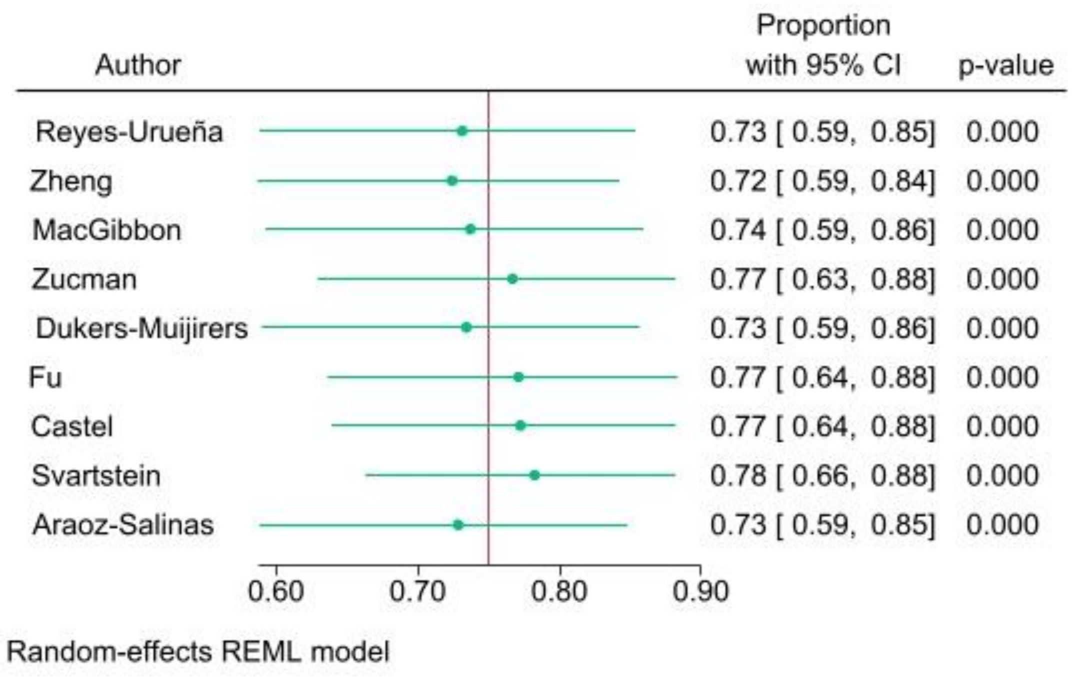

Supplementary Figure 63: Forest plots of the results of leave-one-out sensitivity analysis of the prevalence (%) of mpox vaccine intention among PLHIV (n = 9 studies) pooled using inverse variance weights. Results were based on random-effects meta-analysis. Each green-colored solid square represents the effect size of each study characteristic, while the ends of the adjoining horizontal lines represent lower (left) and upper (right) confidence intervals. All statistics were based on a two-sided t-test. REML, Restricted Maximum Likelihood.

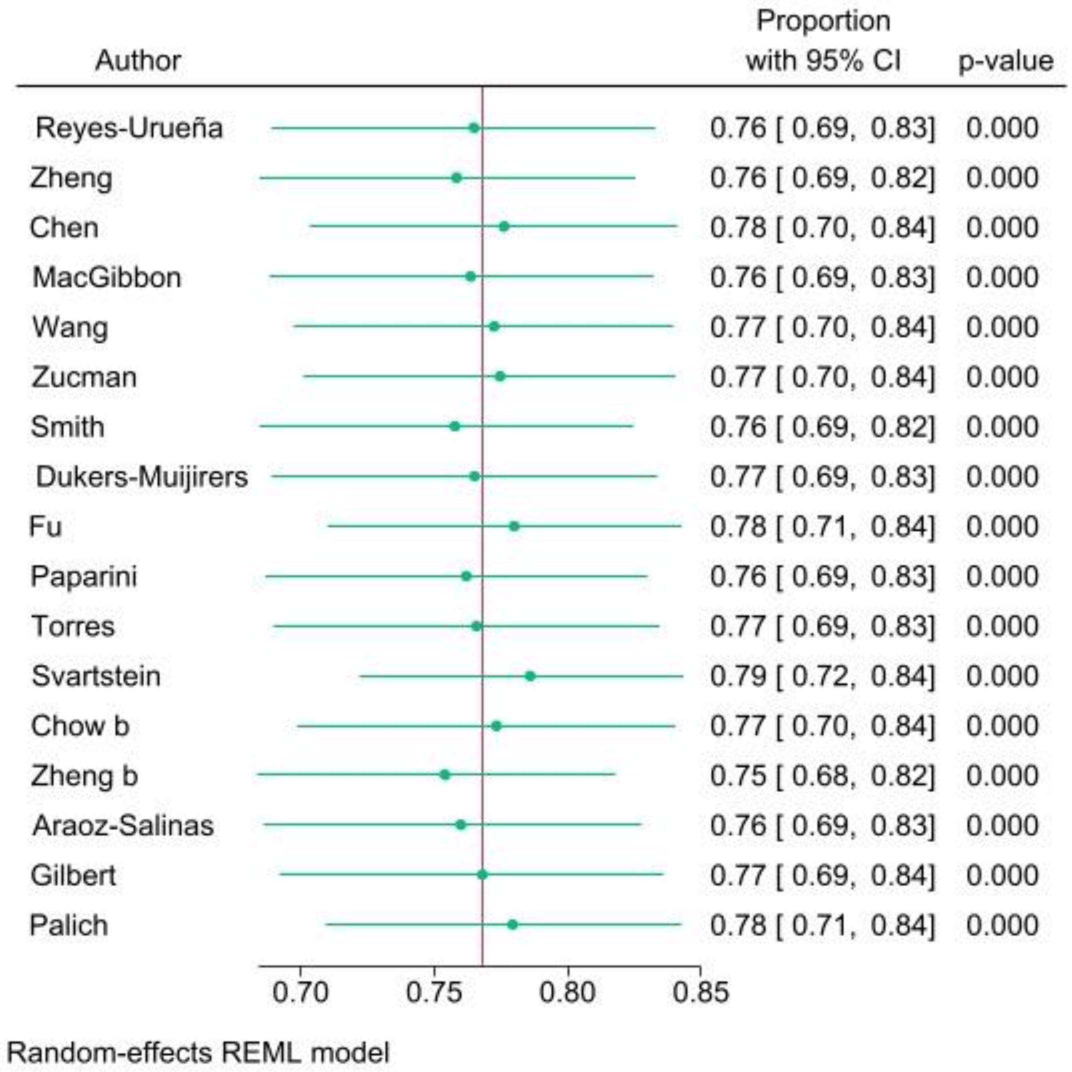

Supplementary Figure 64: Forest plots of the results of leave-one-out sensitivity analysis of the prevalence (%) of mpox vaccine intention among the LGBTQI+ community (n = 17 studies) pooled using inverse variance weights. Results were based on random-effects meta-analysis. Each green-colored solid square represents the effect size of each study characteristic, while the ends of the adjoining horizontal lines represent lower (left) and upper (right) confidence intervals. All statistics were based on a two-sided t-test. REML, Restricted Maximum Likelihood.

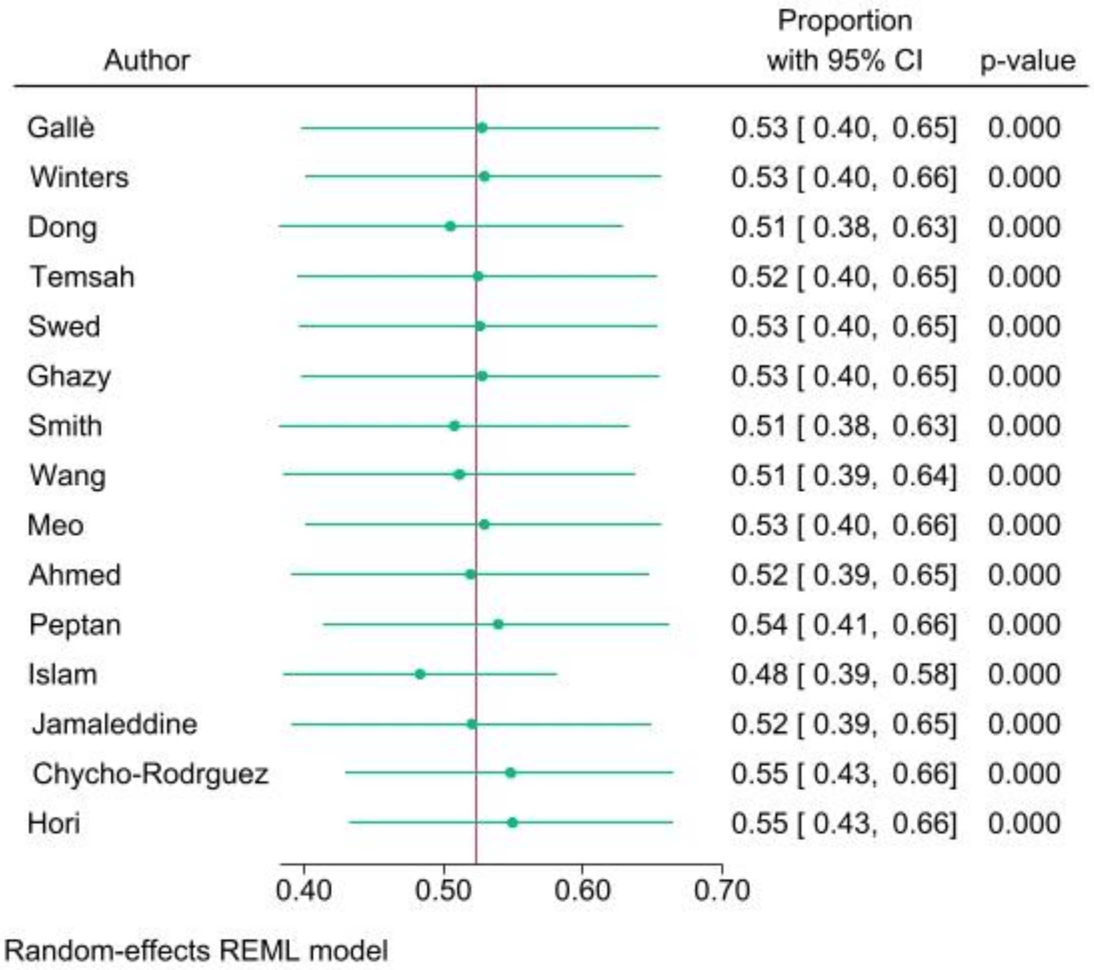

Supplementary Figure 65: Forest plots of the results of leave-one-out sensitivity analysis of the prevalence (%) of mpox vaccine intention among healthcare workers (n = 15 studies) pooled using inverse variance weights. Results were based on random-effects meta-analysis. Each green-colored solid square represents the effect size of each study characteristic, while the ends of the adjoining horizontal lines represent lower (left) and upper (right) confidence intervals. All statistics were based on a two-sided t-test. REML, Restricted Maximum Likelihood.

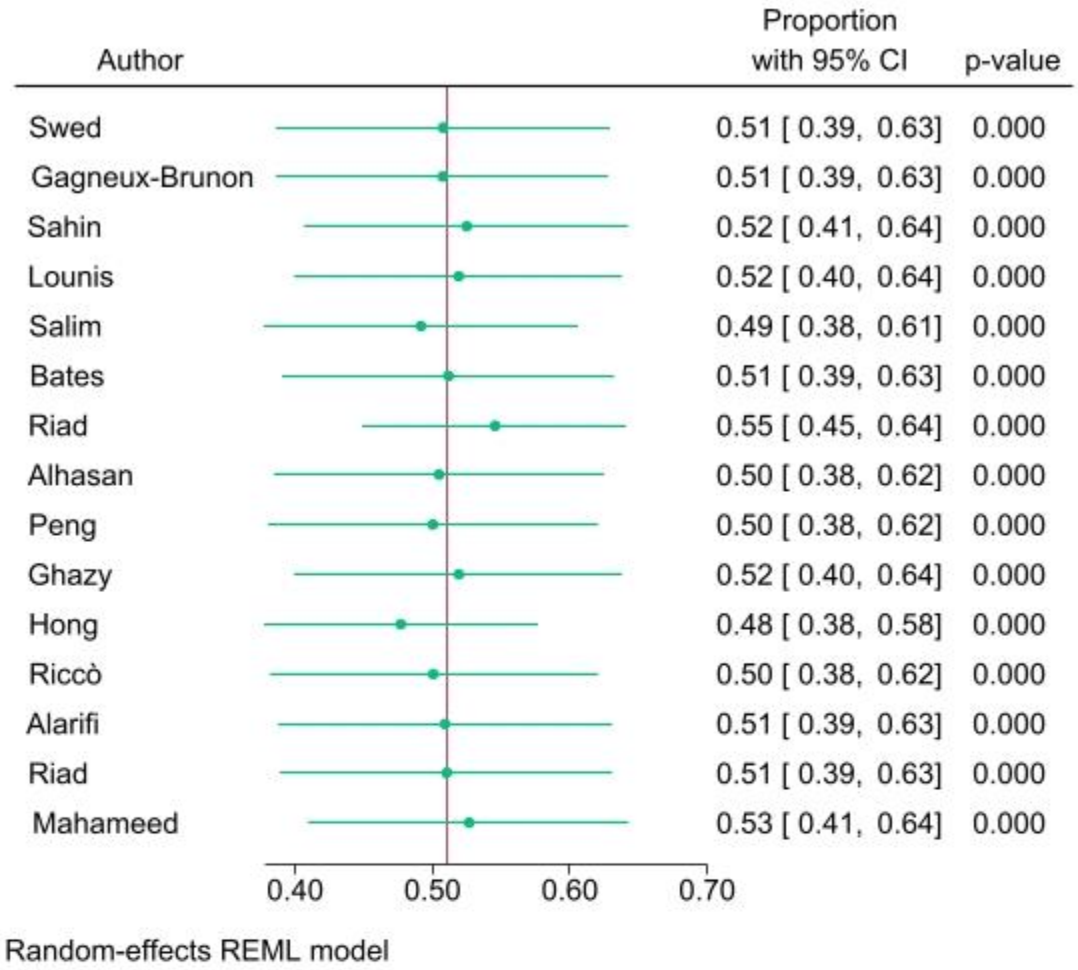

Supplementary Figure 66: Forest plots of the results of leave-one-out sensitivity analysis of the prevalence (%) of mpox vaccine intention among the general public (n = 19 studies) pooled using inverse variance weights. Results were based on random-effects meta-analysis. Each green-colored solid square represents the effect size of each study characteristic, while the ends of the adjoining horizontal lines represent lower (left) and upper (right) confidence intervals. All statistics were based on a two-sided t-test. REML, Restricted Maximum Likelihood.

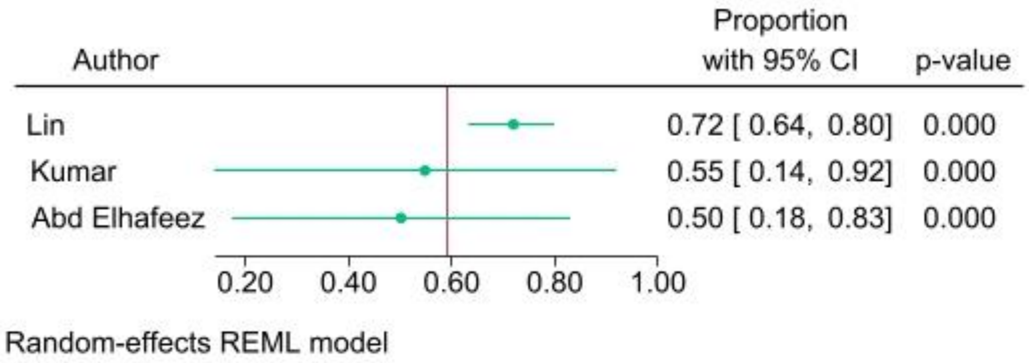

Supplementary Figure 67: Forest plots of the results of leave-one-out sensitivity analysis of the prevalence (%) of mpox vaccine intention among university students (n = 3 studies) pooled using inverse variance weights. Results were based on random-effects

meta-analysis. Each green-colored solid square represents the effect size of each study characteristic, while the ends of the adjoining horizontal lines represent lower (left) and upper (right) confidence intervals. All statistics were based on a two-sided t-test. REML, Restricted Maximum Likelihood.

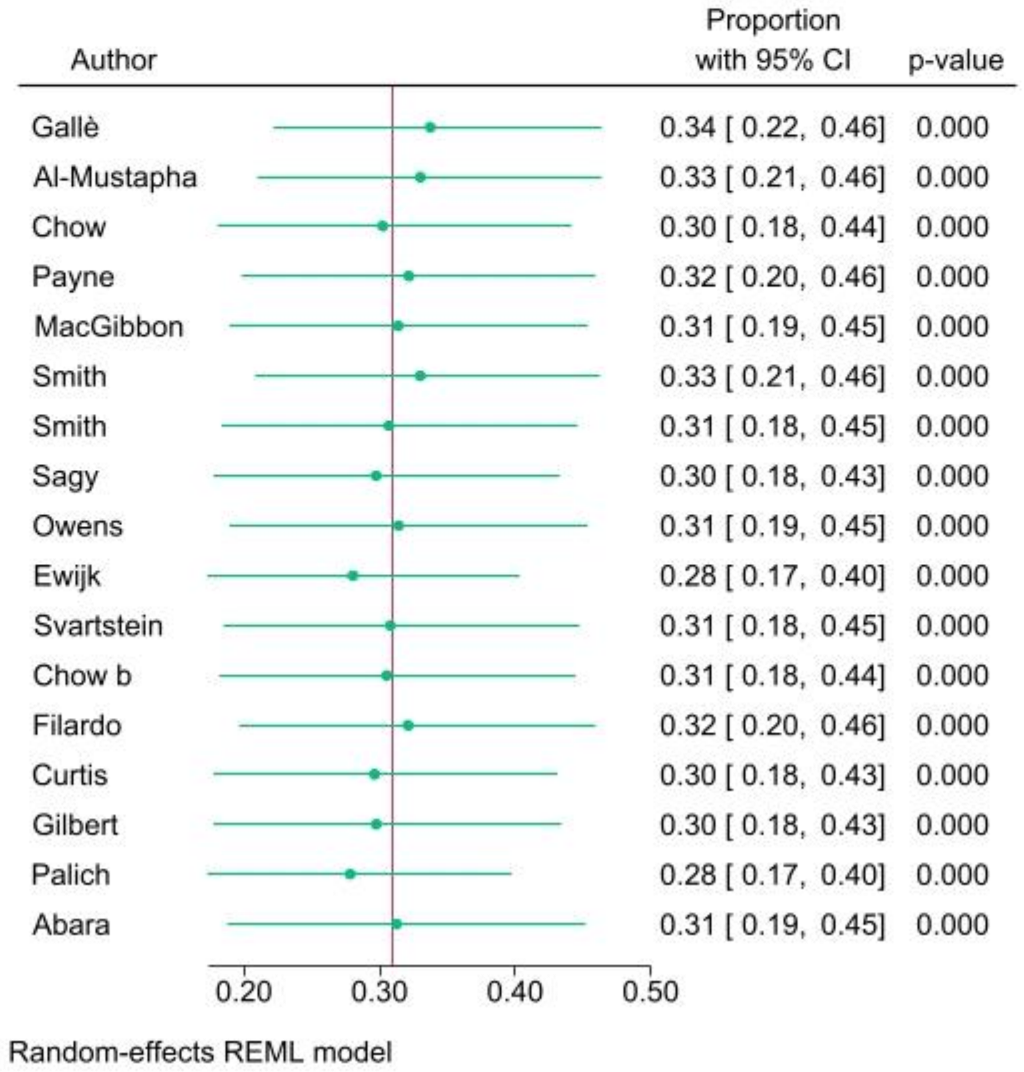

Supplementary Figure 68: Forest plots of the results of leave-one-out sensitivity analysis of the overall prevalence (%) of mpox vaccine uptake globally (n = 17 studies) pooled using inverse variance weights. Results were based on random-effects meta-analysis. Each green-colored solid square represents the effect size of each study characteristic, while the ends of the adjoining horizontal lines represent lower (left) and upper (right) confidence intervals. All statistics were based on a two-sided t-test. REML, Restricted Maximum Likelihood.

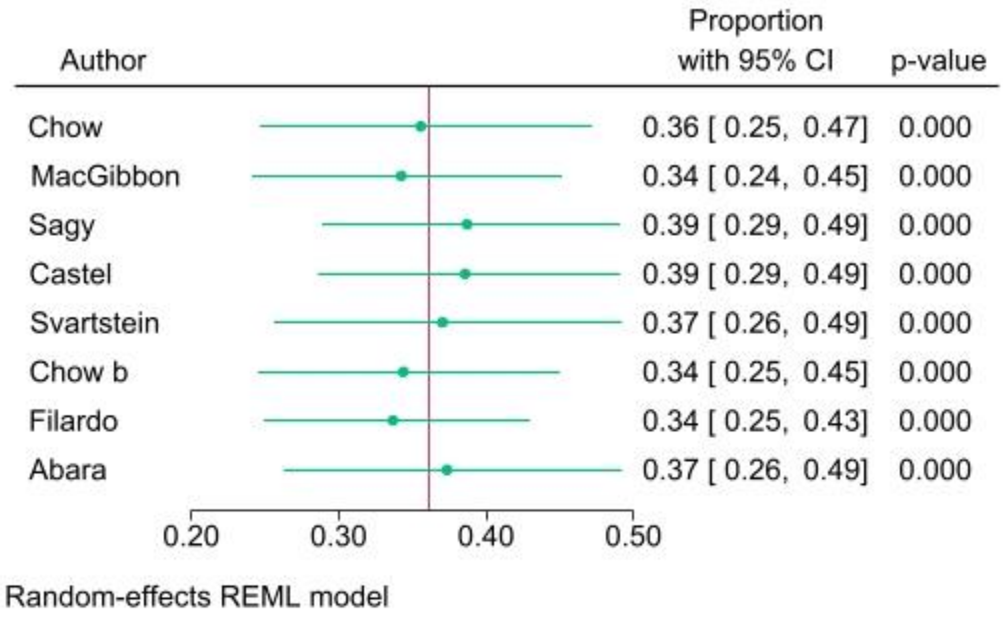

Supplementary Figure 69: Forest plots of the results of leave-one-out sensitivity analysis of the overall prevalence (%) of mpox vaccine uptake among PLHIV (n = 8 studies) pooled using inverse variance weights. Results were based on random-effects meta-analysis. Each green-colored solid square represents the effect size of each study characteristic, while the ends of the adjoining horizontal lines represent lower (left) and upper (right) confidence intervals. All statistics were based on a two-sided t-test. REML, Restricted Maximum Likelihood.

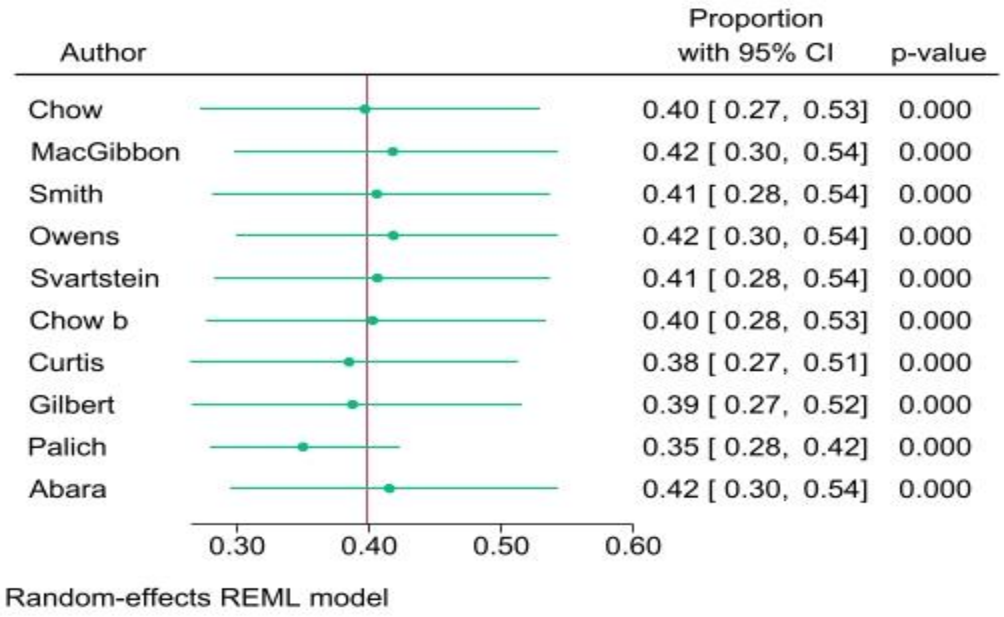

Supplementary Figure 70: Forest plots of the results of leave-one-out sensitivity analysis of the overall prevalence (%) of mpox vaccine uptake among the LGBTQI+ community (n = 10 studies) pooled using inverse variance weights. Results were based on random-effects meta-analysis. Each green-colored solid square represents the effect size of each study characteristic, while the ends of the adjoining horizontal lines represent lower (left) and upper (right) confidence intervals. All statistics were based on a two-sided t-test. REML, Restricted Maximum Likelihood.

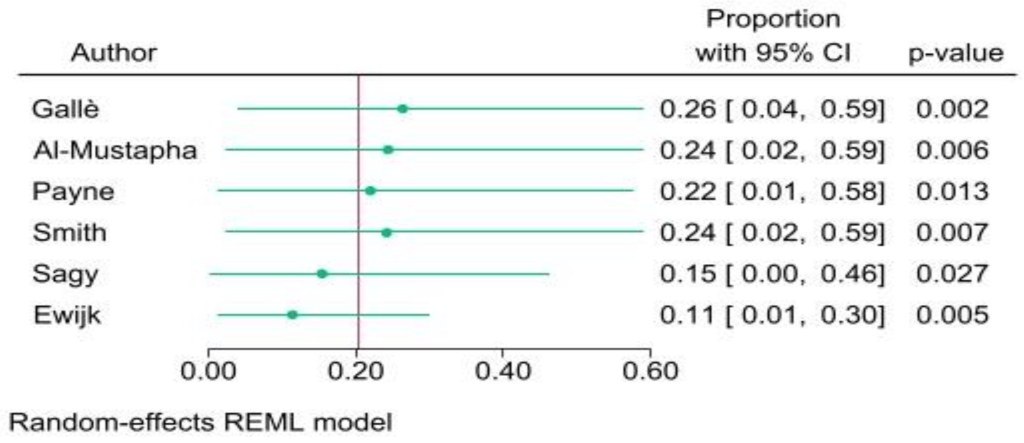

Supplementary Figure 71: Forest plots of the results of leave-one-out sensitivity analysis of the overall prevalence (%) of mpox vaccine uptake among the general public (n = 6 studies) pooled using inverse variance weights. Results were based on random-effects meta-analysis. Each green-colored solid square represents the effect size of each study characteristic, while the ends of the adjoining horizontal lines represent lower (left) and upper (right) confidence intervals. All statistics were based on a two-sided t-test. REML, Restricted Maximum Likelihood.

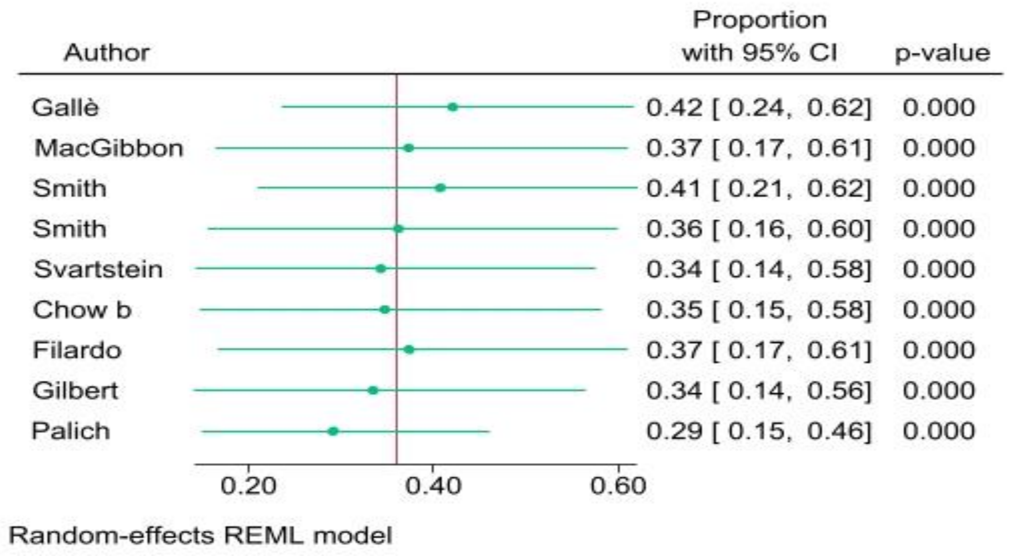

Supplementary Figure 72: Forest plots of the results of leave-one-out sensitivity analysis of the overall prevalence (%) of mpox vaccine uptake among the accepting group globally (n = 9 studies) pooled using inverse variance weights. Results were based on random-effects meta-analysis. Each green-colored solid square represents the effect size of each study characteristic, while the ends of the adjoining horizontal lines represent lower (left) and upper (right) confidence intervals. All statistics were based on a two-sided t-test. REML, Restricted Maximum Likelihood.

Supplementary References (Arranged serially as contained within the main manuscript file)

1. Gallè F, Bianco L, Da Molin G, et al. "Monkeypox: What Do You Know about That?" Italian Adults' Awareness of a New Epidemic. *Pathogens*. 2022;11(11):1285. doi:10.3390/pathogens11111285
2. Al-Mustapha AI, Ogundijo OA, Sikiru NA, et al. A cross-sectional survey of public knowledge of the monkeypox disease in Nigeria. *BMC Public Health*. 2023;23(1):591. doi:10.1186/s12889-023-15398-0
3. Swed S, Alibrahim H, Bohsas H, et al. A multinational cross-sectional study on the awareness and concerns of healthcare providers toward monkeypox and the promotion of the monkeypox vaccination. *Front Public Health*. 2023;11. Accessed November 3, 2023. https://www.frontiersin.org/articles/10.3389/fpubh.2023.1153136
4. Chow EPF, Chen MY, Bradshaw CS, Towns JM, Fairley CK. Accessing first doses of mpox vaccine made available in Victoria, Australia. *Lancet Reg Health – West Pac*. 2023;31. doi:10.1016/j.lanwpc.2023.100712
5. Gagneux-Brunon A, Dauby N, Launay O, Botelho-Nevers E. Attitudes towards monkeypox vaccination among healthcare workers in France and Belgium: an element of complacency? *J Hosp Infect*. 2022;130:144-145. doi:10.1016/j.jhin.2022.09.010
6. Winters M, Malik AA, Omer SB. Attitudes towards Monkeypox vaccination and predictors of vaccination intentions among the US general public. *PLOS ONE*. 2022;17(12):e0278622. doi:10.1371/journal.pone.0278622
7. Reyes-Urueña J, D'Ambrosio A, Croci R, et al. High monkeypox vaccine acceptance among male users of smartphone-based online gay-dating apps in Europe, 30 July to 12 August 2022. *Eurosurveillance*. 2022;27(42):2200757. doi:10.2807/1560-7917.ES.2022.27.42.2200757
8. Payne AB. Reduced Risk for Mpox After Receipt of 1 or 2 Doses of JYNNEOS Vaccine Compared with Risk Among Unvaccinated Persons — 43 U.S. Jurisdictions, July 31–October 1, 2022. *MMWR Morb Mortal Wkly Rep*. 2022;71. doi:10.15585/mmwr.mm7149a5
9. Sahin TK, Erul E, Aksun MS, Sonmezer MC, Unal S, Akova M. Knowledge and Attitudes of Turkish Physicians towards Human Monkeypox Disease and Related Vaccination: A Cross-Sectional Study. *Vaccines*. 2023;11(1):19. doi:10.3390/vaccines11010019
10. Lounis M, Bencherit D, Abdelhadi S. Knowledge and awareness of Algerian healthcare workers about human monkeypox and their attitude toward its vaccination: An online cross-sectional survey. *Vacunas*. Published online February 23, 2023. doi:10.1016/j.vacun.2022.11.003
11. Zheng M, Qin C, Qian X, et al. Knowledge and vaccination acceptance toward the human monkeypox among men who have sex with men in Chin. *Front Public Health*. 2022;10. Accessed November 22, 2022. https://europepmc.org/articles/PMC9640956
12. Dong C, Yu Z, Zhao Y, Ma X. Knowledge and vaccination intention of monkeypox in China's general population: A cross-sectional online survey. *Travel Med Infect Dis*. Published online December 18, 2022:102533. doi:10.1016/j.tmaid.2022.102533
13. Chen Y, Li Y, Fu L, et al. Knowledge of Human Mpox (Monkeypox) and Attitude towards Mpox Vaccination among Male Sex Workers in China: A Cross-Sectional Study. *Vaccines*. 2023;11(2):285. doi:10.3390/vaccines11020285
14. Salim NA, Septadina IS, Permata M, Hudari H. KNOWLEDGE, ATTITUDE, AND PERCEPTION OF ANTICIPATING 2022 GLOBAL HUMAN MONKEYPOX INFECTION AMONG INTERNAL MEDICINE RESIDENTS AT PALEMBANG INDONESIA: AN ONLINE SURVEY. *J Kedokt Dan Kesehat Publ Ilm Fak Kedokt Univ Sriwij*. 2022;9(3):253-262. doi:10.32539/JKK.V9I3.18799
15. Bates BR, Grijalva MJ. Knowledge, attitudes, and practices towards monkeypox during the 2022 outbreak: An online cross-sectional survey among clinicians in Ohio, USA. *J Infect Public Health*. Published online November 11, 2022. doi:10.1016/j.jiph.2022.11.004
16. Lin GSS, Tan WW, Chan DZK, Ooi KS, Hashim H. Monkeypox awareness, knowledge, and attitude among undergraduate preclinical and clinical students at a Malaysian dental school: An emerging outbreak during the COVID-19 era. *Asian Pac J Trop Med*. 2022;15(10):461. doi:10.4103/1995-7645.359787
17. Temsah MH, Aljamaan F, Alenezi S, et al. Monkeypox caused less worry than COVID-19 among the general population during the first month of the WHO Monkeypox alert: Experience from Saudi Arabia. *Travel Med Infect Dis*. 2022;49:102426. doi:10.1016/j.tmaid.2022.102426
18. Riad A, Drobov A, Rozmarinová J, et al. Monkeypox Knowledge and Vaccine Hesitancy of Czech Healthcare Workers: A Health Belief Model (HBM)-Based Study. *Vaccines*. 2022;10(12):2022. doi:10.3390/vaccines10122022
19. MacGibbon J, Cornelisse VJ, Smith AKJ, et al. Mpox (monkeypox) knowledge, concern, willingness to change behaviour, and seek vaccination: results of a national cross-sectional survey. *Sex Health*. 2023;20(5):403-410. doi:10.1071/SH23047
20. Swed S, Bohsas H, Alibrahim H, et al. Monkeypox Post-COVID-19: Knowledge, Worrying, and Vaccine Adoption in the Arabic General Population. *Vaccines*. 2023;11(4):759. doi:10.3390/vaccines11040759
21. Wang H, Paulo KJI d'Abreu de, Gültzow T, Zimmermann HML, Jonas KJ. Monkeypox self-diagnosis abilities, determinants of vaccination and self-isolation intention after diagnosis among MSM, the Netherlands, July 2022. *Eurosurveillance*. 2022;27(33):2200603. doi:10.2807/1560-7917.ES.2022.27.33.2200603

22. Ghazy RM, Yazbek S, Gebreal A, et al. Monkeypox Vaccine Acceptance among Ghanaians: A Call for Action. *Vaccines*. 2023;11(2):240. doi:10.3390/vaccines11020240
23. Zucman D, Fourn E, Touche P, Majerholc C, Vallée A. Monkeypox Vaccine Hesitancy in French Men Having Sex with Men with PrEP or Living with HIV in France. *Vaccines*. 2022;10(10):1629. doi:10.3390/vaccines10101629
24. Smith LE, Potts HW, Brainard J, et al. Did mpox knowledge, attitudes and beliefs affect intended behaviour in the general population and men who are gay, bisexual and who have sex with men? An online cross-sectional survey in the UK. *BMJ Open*. 2023;13(10):e070882. doi:10.1136/bmjopen-2022-070882
25. Alhasan K, Sallam M, Aljamaan F, et al. Mpox Perceptions and Vaccine Advocacy among the Healthcare Workers of Solid Organ Transplant Centers: A Multicenter, Cross-Sectional Survey in Saudi Arabia. *Healthcare*. 2023;11(4):603. doi:10.3390/healthcare11040603
26. Dukers-Muijrers NHTM, Evers Y, Widdershoven V, et al. Mpox vaccination willingness, determinants, and communication needs in gay, bisexual, and other men who have sex with men, in the context of limited vaccine availability in the Netherlands (Dutch Mpox-survey). *Front Public Health*. 2023;10:1058807. doi:10.3389/fpubh.2022.1058807
27. Fu L, Sun Y, Li Y, et al. Perception of and Vaccine Readiness towards Mpox among Men Who Have Sex with Men Living with HIV in China: A Cross-Sectional Study. *Vaccines*. 2023;11(3):528. doi:10.3390/vaccines11030528
28. Peng X, Wang B, Li Y, et al. Perceptions and worries about monkeypox, and attitudes towards monkeypox vaccination among medical workers in China: A cross-sectional survey. *J Infect Public Health*. 2023;16(3):346-353. doi:10.1016/j.jiph.2023.01.010
29. Wang B, Peng X, Li Y, et al. Perceptions, precautions, and vaccine acceptance related to monkeypox in the public in China: A cross-sectional survey. *J Infect Public Health*. 2023;16(2):163-170. doi:10.1016/j.jiph.2022.12.010
30. Ghazy RM, Okeh DU, Sallam M, et al. Psychological Antecedents of Healthcare Workers towards Monkeypox Vaccination in Nigeria. *Vaccines*. 2022;10(12):2151. doi:10.3390/vaccines10122151
31. Meo SA, Al-Khlaiwi T, Aljofan ZF, Alanazi AI, Meo AS. Public Perceptions of the Emerging Human Monkeypox Disease and Vaccination in Riyadh, Saudi Arabia: A Cross-Sectional Study. *Vaccines*. 2022;10(9):1534. doi:10.3390/vaccines10091534
32. Paparini S, Whitacre R, Smuk M, et al. Public understanding, awareness, and response to monkeypox virus outbreak: A cross-sectional survey of the most affected communities in the United Kingdom during the 2022 public health emergency. Published online August 25, 2022:2022.08.25.22279207. doi:10.1101/2022.08.25.22279207
33. Sagy YW, Zucker R, Hammerman A, et al. Real-world effectiveness of a single dose of mpox vaccine in males. *Nat Med*. Published online January 31, 2023:1-1. doi:10.1038/s41591-023-02229-3
34. Owens C, Hubach RD. Rural-urban differences in monkeypox behaviors and attitudes among men who have sex with men in the United States. *J Rural Health*. n/a(n/a). doi:10.1111/jrh.12726
35. Ahmed SK, Abdulqadir SO, Omar RM, et al. Knowledge, Attitude and Worry in the Kurdistan Region of Iraq during the Mpox (Monkeypox) Outbreak in 2022: An Online Cross-Sectional Study. *Vaccines*. 2023;11(3):610. doi:10.3390/vaccines11030610
36. Peptan C, Băleanu VD, Mărcău FC. Study on the Vaccination of the Population of Romania against Monkeypox in Terms of Medical Security. *Vaccines*. 2022;10(11):1834. doi:10.3390/vaccines10111834
37. Hong J, Pan B, Jiang HJ, et al. The willingness of Chinese healthcare workers to receive monkeypox vaccine and its independent predictors: A cross-sectional survey. *J Med Virol*. Published online November 11, 2022:e28294. doi:10.1002/jmv.28294
38. Riccò M, Ferraro P, Camisa V, et al. When a Neglected Tropical Disease Goes Global: Knowledge, Attitudes and Practices of Italian Physicians towards Monkeypox, Preliminary Results. *Trop Med Infect Dis*. 2022;7(7):135. doi:10.3390/tropicalmed7070135
39. Alarifi AM, Alshahrani NZ, Sah R. Are Saudi Healthcare Workers Willing to Receive the Monkeypox Virus Vaccine? Evidence from a Descriptive-Baseline Survey. *Trop Med Infect Dis*. 2023;8(8):396. doi:10.3390/tropicalmed8080396
40. Kumar N, Ahmed F, Raza MS, et al. Monkeypox Cross-Sectional Survey of Knowledge, Attitudes, Practices, and Willingness to Vaccinate among University Students in Pakistan. *Vaccines*. 2023;11(1):97. doi:10.3390/vaccines11010097
41. Abd ElHafeez S, Gebreal A, Khalil MA, et al. Assessing disparities in medical students' knowledge and attitude about monkeypox: a cross-sectional study of 27 countries across three continents. *Front Public Health*. 2023;11:1192542. doi:10.3389/fpubh.2023.1192542
42. Islam MR, Haque MA, Ahamed B, et al. Assessment of vaccine perception and vaccination intention of Mpox infection among the adult males in Bangladesh: A cross-sectional study findings. *PLOS ONE*. 2023;18(6):e0286322. doi:10.1371/journal.pone.0286322
43. Riad A, Rybakova N, Dubatouka N, et al. Belarusian Healthcare Professionals' Views on Monkeypox and Vaccine Hesitancy. *Vaccines*. 2023;11(8):1368. doi:10.3390/vaccines11081368
44. Salih T. Demographic Disparities in Mpox Vaccination Series Completion, by Route of Vaccine Administration — California, August 9, 2022–March 31, 2023. *MMWR Morb Mortal Wkly Rep*. 2023;72. doi:10.15585/mmwr.mm7230a4
45. Torres TS, Silva MST, Coutinho C, et al. Evaluation of Mpox Knowledge, Stigma, and Willingness to Vaccinate for Mpox: Cross-Sectional Web-Based Survey Among Sexual and Gender Minorities. *JMIR Public Health Surveill*. 2023;9(1):e46489.

doi:10.2196/46489

46. JAMALEDDINE Y, EL EZZ AA, MAHMOUD M, et al. Knowledge and attitude towards monkeypox among the Lebanese population and their attitude towards vaccination. *J Prev Med Hyg*. 2023;64(1):E13-E26. doi:10.15167/2421-4248/jpmh2023.64.1.2903
47. van Ewijk CE, Smit C, Bavalia R, et al. Acceptance and timeliness of post-exposure vaccination against mpox in high-risk contacts, Amsterdam, the Netherlands, May–July 2022. *Vaccine*. 2023;41(47):6952-6959. doi:10.1016/j.vaccine.2023.10.013
48. Castel AD, Andersen E, Monroe A, et al. Mpox Awareness, Risk Reduction, and Vaccine Acceptance among Pwh in Washington, Dc. *Top Antivir Med*. Published online 2023:401-402.
49. Svartstein ASW, Knudsen AD, Heidari SL, et al. Mpox Incidence and Vaccine Uptake in Men Who Have Sex with Men and Are Living with HIV in Denmark. *Vaccines*. 2023;11(7):1167. doi:10.3390/vaccines11071167
50. Chow EPF, Samra RS, Bradshaw CS, et al. Mpox knowledge, vaccination and intention to reduce sexual risk practices among men who have sex with men and transgender people in response to the 2022 mpox outbreak: a cross-sectional study in Victoria, Australia. *Sex Health*. Published online July 10, 2023. doi:10.1071/SH23075
51. Zheng M, Du M, Yang G, et al. Mpox Vaccination Hesitancy and Its Associated Factors among Men Who Have Sex with Men in China: A National Observational Study. *Vaccines*. 2023;11(9):1432. doi:10.3390/vaccines11091432
52. Filardo TD, Prasad N, Waddell CJ, et al. Mpox vaccine acceptability among people experiencing homelessness in San Francisco — October–November 2022. *Vaccine*. 2023;41(39):5673-5677. doi:10.1016/j.vaccine.2023.07.068
53. Araoz-Salinas JM, Ortiz-Saavedra B, Ponce-Rosas L, et al. Perceptions and Intention to Get Vaccinated against Mpox among the LGBTIQ+ Community during the 2022 Outbreak: A Cross-Sectional Study in Peru. *Vaccines*. 2023;11(5):1008. doi:10.3390/vaccines11051008
54. Curtis MG, Davoudpour S, Rodriguez-Ortiz AE, et al. Predictors of Mpox vaccine uptake among sexual and gender minority young adults living in Illinois: Unvaccinated vs. double vs. single dose vaccine recipients. *Vaccine*. 2023;41(27):4002-4008. doi:10.1016/j.vaccine.2023.05.043
55. Mahameed H, Al-Mahzoum K, AlRaie LA, et al. Previous Vaccination History and Psychological Factors as Significant Predictors of Willingness to Receive Mpox Vaccination and a Favorable Attitude towards Compulsory Vaccination. *Vaccines*. 2023;11(5):897. doi:10.3390/vaccines11050897
56. Caycho-Rodríguez T, Tomás JM, Vilca LW, et al. Relationship Between Fear of Monkeypox and Intention to be Vaccinated Against Monkeypox in a Peruvian Sample. The Mediating Role of Conspiracy Beliefs About Monkeypox. *Eval Health Prof*. Published online May 29, 2023:01632787231180195. doi:10.1177/01632787231180195
57. Hori D, Kaneda Y, Ozaki A, Tabuchi T. Sexual orientation was associated with intention to be vaccinated with a smallpox vaccine against mpox: A cross-sectional preliminary survey in Japan. *Vaccine*. 2023;41(27):3954-3959. doi:10.1016/j.vaccine.2023.05.050
58. Gilbert M, Ablona A, Chang HJ, et al. Uptake of Mpox vaccination among transgender people and gay, bisexual and other men who have sex with men among sexually-transmitted infection clinic clients in Vancouver, British Columbia. *Vaccine*. 2023;41(15):2485-2494. doi:10.1016/j.vaccine.2023.02.075
59. Zucker R, Wolff-Sagy Y, Ramot N, et al. Examining the Patterns of Mpox Vaccine Uptake in a Vulnerable Population. *Sex Transm Dis*.:10.1097/OLQ.0000000000001839. doi:10.1097/OLQ.0000000000001839
60. Palich R, Jedrzejewski T, Schneider L, et al. High uptake of vaccination against mpox in men who have sex with men (MSM) on HIV pre-exposure prophylaxis (PrEP) in Paris, France. *Sex Transm Infect*. Published online July 28, 2023. doi:10.1136/sextrans-2023-055885
61. Abara WE, Sullivan P, Carpino T, et al. Characteristics of mpox vaccine recipients among a sample of men who have sex with men with presumed exposure to mpox. *Sex Transm Dis*. 2023;50(7):458-461. doi:10.1097/OLQ.0000000000001800
