# Supplementary material for: Global prevalence and correlates of mpox vaccine acceptance and uptake: a systematic review and meta-analysis
Source: Commun Med (Lond). 2024 Jul 9;4:136. doi: 10.1038/s43856-024-00564-1 (PMC11231226; doi:10.1038/s43856-024-00564-1)
Supplement: Supplementary file 3 — Description of Additional Supplementary Files [file 43856_2024_564_MOESM3_ESM.pdf]

Description of Additional Supplementary Files

File name: Supplementary Data 1

File Description: The literature search strategy used across all databases

File name: Supplementary Data 2

File Description: Results of the consensus critical appraisal of the included cross-sectional studies using the Newcastle-Ottawa scale for cross-sectional studies

File name: Supplementary Data 3

File Description: Summary of the included studies

File name: Supplementary Data 4

File Description: Prevalence rates of mpox vaccine acceptance, intention, and uptake across populations as derived from meta-analysis
