# Supplementary material for: Global prevalence and correlates of mpox vaccine acceptance and uptake: a systematic review and meta-analysis
Source: Commun Med (Lond). 2024 Jul 9;4:136. doi: 10.1038/s43856-024-00564-1 (PMC11231226; doi:10.1038/s43856-024-00564-1)
Supplement: Supplementary file 8 — Reporting Summary [file 43856_2024_564_MOESM8_ESM.pdf]

nature portfolio

Corresponding author(s): Sahabi Kabir Sulaiman

Last updated by author(s): Dec 31, 2023

Reporting Summary

Nature Portfolio wishes to improve the reproducibility of the work that we publish. This form provides structure for consistency and transparency in reporting. For further information on Nature Portfolio policies, see our Editorial Policies and the Editorial Policy Checklist.

Statistics

For all statistical analyses, confirm that the following items are present in the figure legend, table legend, main text, or Methods section.

n/a	Confirmed	
☐	☒	The exact sample size (*n*) for each experimental group/condition, given as a discrete number and unit of measurement
☐	☒	A statement on whether measurements were taken from distinct samples or whether the same sample was measured repeatedly
☐	☒	The statistical test(s) used AND whether they are one- or two-sided<br>*Only common tests should be described solely by name; describe more complex techniques in the Methods section.*
☐	☒	A description of all covariates tested
☐	☒	A description of any assumptions or corrections, such as tests of normality and adjustment for multiple comparisons
☐	☒	A full description of the statistical parameters including central tendency (e.g. means) or other basic estimates (e.g. regression coefficient) AND variation (e.g. standard deviation) or associated estimates of uncertainty (e.g. confidence intervals)
☐	☒	For null hypothesis testing, the test statistic (e.g. *F*, *t*, *r*) with confidence intervals, effect sizes, degrees of freedom and *P* value noted<br>*Give P values as exact values whenever suitable.*
☒	☐	For Bayesian analysis, information on the choice of priors and Markov chain Monte Carlo settings
☒	☐	For hierarchical and complex designs, identification of the appropriate level for tests and full reporting of outcomes
☐	☒	Estimates of effect sizes (e.g. Cohen's *d*, Pearson's *r*), indicating how they were calculated

Our web collection on statistics for biologists contains articles on many of the points above.

Software and code

Policy information about availability of computer code

Data collection	The Zotero software (version 6.0.15) , Rayyan QRCI and Microsoft Excel were used for data extraction.
Data analysis	Stata Version 15IC (StataCorp, College Station, Texas USA)

For manuscripts utilizing custom algorithms or software that are central to the research but not yet described in published literature, software must be made available to editors and reviewers. We strongly encourage code deposition in a community repository (e.g. GitHub). See the Nature Portfolio guidelines for submitting code & software for further information.

Data

Policy information about availability of data

All manuscripts must include a data availability statement. This statement should provide the following information, where applicable:

- Accession codes, unique identifiers, or web links for publicly available datasets
- A description of any restrictions on data availability
- For clinical datasets or third party data, please ensure that the statement adheres to our policy

All the data supporting the findings of this work can be accessed via the Open Science Framework at https://doi.org/10.17605/OSF.IO/FS5QH

Research involving human participants, their data, or biological material

Policy information about studies with human participants or human data. See also policy information about sex, gender (identity/presentation), and sexual orientation and race, ethnicity and racism.

Reporting on sex and gender	Self-reported gender was considered herein, and reported as male or female.
Reporting on race, ethnicity, or other socially relevant groupings	Not applicable.
Population characteristics	Any study reporting on the outcomes of interest in any population group was considered eligible and included.
Recruitment	Existing peer-reviewed publication findings were used.
Ethics oversight	International Prospective Register for Systematic reviews (PROSPERO)

Note that full information on the approval of the study protocol must also be provided in the manuscript.

Field-specific reporting

Please select the one below that is the best fit for your research. If you are not sure, read the appropriate sections before making your selection.

☐ Life sciences ☒ Behavioural & social sciences ☐ Ecological, evolutionary & environmental sciences

For a reference copy of the document with all sections, see nature.com/documents/nr-reporting-summary-flat.pdf

Behavioural & social sciences study design

All studies must disclose on these points even when the disclosure is negative.

Study description	This was a systematic review and meta-analyses of peer-reviewed studies reporting on mpox vaccine acceptance, intention and/or uptake in any population.
Research sample	LGBTQI+ (lesbian, gay, bisexual, transgender, queer, and intersex) community, healthcare workers, general public, Adult people living with HIV based on reports from original studies globally
Sampling strategy	No limitation to individual study sampling strategy was applied. Hence, all eligible studies were included regardless of the sampling method employed.
Data collection	The Joanna Briggs Institute data extraction form was used by two authors (SKS and MSM) to extract data from all included studies.
Timing	Only studies published between May 2022 to 25th November 2023 were included.
Data exclusions	Only peer-reviewed articles published in the English language were included
Non-participation	Not applicable
Randomization	Not applicable

Reporting for specific materials, systems and methods

We require information from authors about some types of materials, experimental systems and methods used in many studies. Here, indicate whether each material, system or method listed is relevant to your study. If you are not sure if a list item applies to your research, read the appropriate section before selecting a response.

Materials & experimental systems

n/a	Involved in the study
☒	☐ Antibodies
☒	☐ Eukaryotic cell lines
☒	☐ Palaeontology and archaeology
☒	☐ Animals and other organisms
☒	☐ Clinical data
☒	☐ Dual use research of concern
☒	☐ Plants

Methods

n/a	Involved in the study
☒	☐ ChIP-seq
☒	☐ Flow cytometry
☒	☐ MRI-based neuroimaging

Plants

Seed stocks	Not applicable.
Novel plant genotypes	Not applicable.
Authentication	Not applicable.
